# The *Legionella pneumophila* genome evolved to accommodate multiple regulatory mechanisms controlled by the CsrA-system

**DOI:** 10.1371/journal.pgen.1006629

**Published:** 2017-02-17

**Authors:** Tobias Sahr, Christophe Rusniok, Francis Impens, Giulia Oliva, Odile Sismeiro, Jean-Yves Coppée, Carmen Buchrieser

**Affiliations:** 1 Institut Pasteur, Biologie des Bactéries Intracellulaires, Paris France; 2 CNRS UMR 3525, Paris, France; 3 Institut Pasteur, Unité des Interactions Bactéries-Cellules, Inserm U604, INRA Unité sous-contrat, Paris, France; 4 VIB-UGent Center for Medical Biotechnology, Ghent University, Ghent, Belgium; 5 Institut Pasteur, Transcriptome and EpiGenome, BioMics, Center for Innovation and Technological Research, Paris, France; University of Würzburg, GERMANY

## Abstract

The carbon storage regulator protein CsrA regulates cellular processes post-transcriptionally by binding to target-RNAs altering translation efficiency and/or their stability. Here we identified and analyzed the direct targets of CsrA in the human pathogen *Legionella pneumophila*. Genome wide transcriptome, proteome and RNA co-immunoprecipitation followed by deep sequencing of a wild type and a *csrA* mutant strain identified 479 RNAs with potential CsrA interaction sites located in the untranslated and/or coding regions of mRNAs or of known non-coding sRNAs. Further analyses revealed that CsrA exhibits a dual regulatory role in virulence as it affects the expression of the regulators FleQ, LqsR, LetE and RpoS but it also directly regulates the timely expression of over 40 Dot/Icm substrates. CsrA controls its own expression and the stringent response through a regulatory feedback loop as evidenced by its binding to RelA-mRNA and links it to quorum sensing and motility. CsrA is a central player in the carbon, amino acid, fatty acid metabolism and energy transfer and directly affects the biosynthesis of cofactors, vitamins and secondary metabolites. We describe the first *L*. *pneumophila* riboswitch, a thiamine pyrophosphate riboswitch whose regulatory impact is fine-tuned by CsrA, and identified a unique regulatory mode of CsrA, the active stabilization of RNA anti-terminator conformations inside a coding sequence preventing Rho-dependent termination of the *gap* operon through transcriptional polarity effects. This allows *L*. *pneumophila* to regulate the pentose phosphate pathway and the glycolysis combined or individually although they share genes in a single operon. Thus the *L*. *pneumophila* genome has evolved to acclimate at least five different modes of regulation by CsrA giving it a truly unique position in its life cycle.

## Author summary

The RNA binding protein CsrA is the master regulator of the bi-phasic life cycle of *Legionella pneumophila* governing virulence expression in this intracellular pathogen. Here, we have used deep sequencing of RNA enriched by co-immunoprecipitation with epitope-tagged CsrA to identify CsrA-associated transcripts at the genome level. We found 479 mRNAs or non-coding RNAs to be targets of CsrA. Among those major regulators including FleQ, the regulator of flagella expression, LqsR, the regulator of quorum sensing and RpoS implicated in stress response were identified. The expression of over 40 type IV secreted effector proteins important for intracellular survival and virulence are under the control of CsrA. Combined with transcriptomics, whole shotgun proteomics of a wild type and a CsrA mutant strain and functional analyses of several CsrA-targeted RNAs we identified the first riboswitch in *L*. *pneumophila*, a thiamine pyrophosphate riboswitch, and discovered a new mode of regulation by CsrA that allows *L*. *pneumophila* to regulate the pentose phosphate pathway and the glycolysis combined or individually although they share genes in a single operon. Our results further underline the indispensable role of CsrA in the life cycle of *L*. *pneumophila* and provide new insights into its regulatory roles and mechanisms.

## Introduction

The Gram negative, environmental bacterium *Legionella pneumophila* is proliferating in aquatic environments where it parasitizes in fresh water protozoa [1–3]. When contaminated water is aerosolized, mainly within man-made devices and installations, *L*. *pneumophila* can gain access to the human lung and cause a severe pneumonia called Legionnaires’ disease [4]. The capacity of this environmental bacterium to cause disease in humans evolved from the interaction with aquatic amoebae, as the same strategies used for persistence in protozoa also allow this pathogen also to replicate within alveolar macrophages [5, 6]. In amoeba as well as in human macrophages the *L*. *pneumophila* life cycle consists of two distinct stages, a replicative form that proliferates when nutrients are available and a transmissive or virulent form that is able to escape from the spent host when nutrients are exhausted and to infect a new host cell [7, 8]. In the transmissive form traits like virulence, motility and resistance against several stress factors are induced, whereas these are typically repressed during replication [8, 9].

A key regulator of the switch between replicative and transmissive *L*. *pneumophila* is the RNA-binding protein CsrA [10, 11]. CsrA is a global, posttranscriptional regulator of gene expression in many bacteria where it plays important roles in regulating motility, virulence and metabolism [12]. To fulfill its regulatory role, CsrA binds to the 5’ untranslated region (5’ UTR) or in the start region of the coding sequence of the mRNA of its target genes. CsrA modulates translation, and alters mRNA turnover and/or transcript elongation [12, 13]. The current model of the *L*. *pneumophila* life cycle regulation is that starvation of amino acids and altered fatty acid biosynthesis lead to the production of (p)ppGpp and subsequently the activation of the two-component system (TCS) LetA/LetS and the alternative sigma factor RpoS [14, 15]. Both promote the transcription of the small non-coding RNAs RsmX, RsmY and RsmZ, which in turn bind and sequester CsrA leading to the expression of transmissive and repression of replicative traits [16–18]. The *letA/S*-mutants instead are non-motile, less pigmented, sodium stress resistant, but oxidative stress sensitive and also show a reduced infectivity for *A*. *castellanii* [16–18]. Analyses of a strain overexpressing CsrA or a conditional *csrA*-mutant revealed that CsrA represses typical post exponential (PE), transmissive phenotypes of *L*. *pneumophila* such as cell shape shortening, pigmentation, motility, sodium sensitivity and cytotoxicity [10, 11, 19]. Additionally, the quorum sensing regulatory system LqsTS/LqsR and the TCS PmrB/PmrA regulate CsrA activity [16, 20–23]. In *Legionella*, PmrBA was shown to regulate the expression of several Dot/Icm effector proteins and positively regulates the transcription of *csrA* [16, 24].

Different studies have reported indirect evidence linking *L*. *pneumophila* CsrA and virulence by identifying putative CsrA binding motifs in the mRNAs of secreted Dot/Icm effectors, or analyzing CsrA overexpressing strains [10, 11, 16, 25]. However, the direct targets of CsrA and whether these are regulated by the classical regulatory mechanism described for CsrA or not, are not known. By using transcriptomics, proteomics, RNA-Immunoprecipitation followed by deep sequencing (RIPseq), together with biochemical, phenotypical and molecular analyses we identified the *L*. *pneumophila* CsrA targets genome wide and discovered a new mode of action of CsrA that allows to regulate genes comprised in the same operon, independently.

## Results

### A *L*. *pneumophila csrA*-mutant exhibits a transmissive phenotype already during exponential growth and is strongly attenuated in intracellular replication

To study the regulatory consequences of CsrA in detail, we constructed a mutant *csrA*^*-*^ by inserting an apramycin-resistance cassette after the amino acid Tyr48 of the *lpp0845* gene encoding the major CsrA in *L*. *pneumophila* Paris (**S1A Fig**). CsrA is essential for *L*. *pneumophila*, but such a truncated CsrA variant has a strongly reduced expression of CsrA (**S1B Fig**), similarly to what was reported for *Escherichia coli* [26, 27], possibly by immediate degradation of a miss folded protein due to the distorted C-terminal helical structure. Furthermore, it has been reported that mutations leading to a dislocation of the alpha-helix are totally devoid of biological activity [28], thus allowing to study the RNA targets of CsrA. Indeed, when analyzing flagelllin expression, which is a hallmark of the PE-phase in *L*. *pneumophila* it was expressed already in E-phase in the *csrA*^*-*^ strain indicating that CsrA mediated repression in E phase was released (**S1C Fig**). Phenotypic analyses of the mutant showed that in contrast to the wt strain the *csrA*^*-*^ strain was motile already in E phase, as judged by microscopic observation of actively moving bacteria. Furthermore it showed a significantly higher pigment production and sodium sensitivity, a phenotype that reflects activity of the Dot/Icm T4SS system (**S1D and S1E Fig**). Furthermore, the *csrA*^*-*^ strain was more resistant to oxidative stress (**S1F Fig**) and showed increased tolerance against moderate acidification (pH 4.8) as compared to the wt (**S1G Fig**). Thus, a *csrA* mutant shows clear evidence of a transmissive/virulent phenotype already during exponential growth (replication).

**Fig 1:**
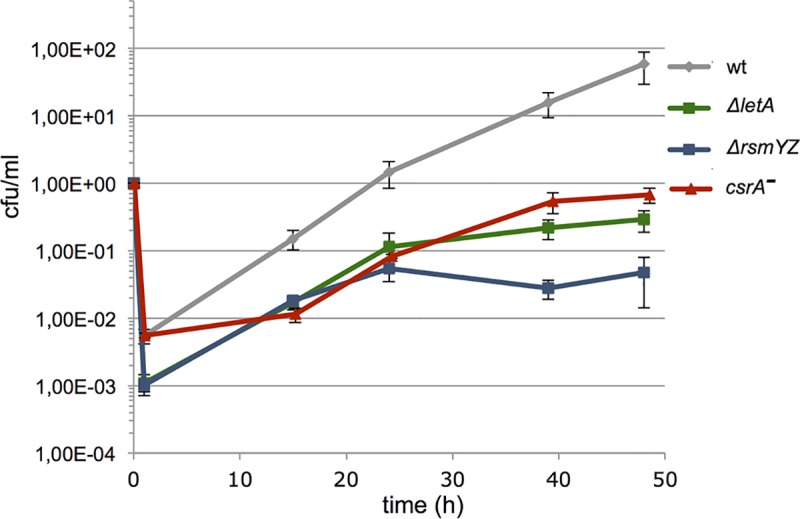
The *L*. *pneumophila csrA*^*-*^ mutant shows a defect in intracellular growth. The *L*. *pneumophila csrA*^*-*^ mutant has a defect in replication in *Acanthamoeba castellanii*. Grey, wt *L*. *pneumophila* strain Paris; green, Δ*letA*-mutant; Blue, Δ*rsmYZ* double mutant; Red, *csrA*^*—*^mutant. Infections were performed at 37°C and the number of intracellular, viable bacteria was determined by the standard plate count assay. Each time point represents the mean +/- SD of three biological replicates.

To evaluate the impact of CsrA on intracellular replication we compared its growth within *Acanthamoeba castellanii* to that of the wt strain, a Δ*letA* and a Δ*rsmY/Z* double mutant [17]. As shown in **Fig 1**, mutation of the *csrA* locus has a drastic effect on the overall replication capacity (100x less than wt) comparable to what is observed for the RsmYZ and LetA mutants. However, the Δ*letA* and Δ*rsmYZ* strains exhibit both a strong defect in the entry and the initial stages of infection (1h) but once established in the host cell, they show similar replication efficiency as the wt strain during the first infection cycle (between 1h and 24h), but are not replicating anymore during the second infection cycle (>24h). In contrast, the entry of the *csrA*^*-*^ and the wt strain is identical, but then replication is significantly diminished in absence of CsrA, in particular during the first infection cycle up to 15h post infection and at the end of the second infection cycle (**Fig 1**). Similar results were observed for a conditional *csrA*^*-*^strain when infecting bone-marrow-derived macrophages [11]. Thus the Δ*letA* and Δ*rsmYZ* strains seem to be halted in the non-virulent, replicative phase and are not able to efficiently infect amoeba [17, 29] whereas the *csrA*^*-*^ strain seems to be forced into a premature transmissive phenotype with a strong deficit in the reconversion from the virulent into the replicative stage.

### Genome-wide differential proteomics and transcriptomics reveal that CsrA impacts flagella biosynthesis, central carbon flux, stress response and virulence

**Fig 2:**
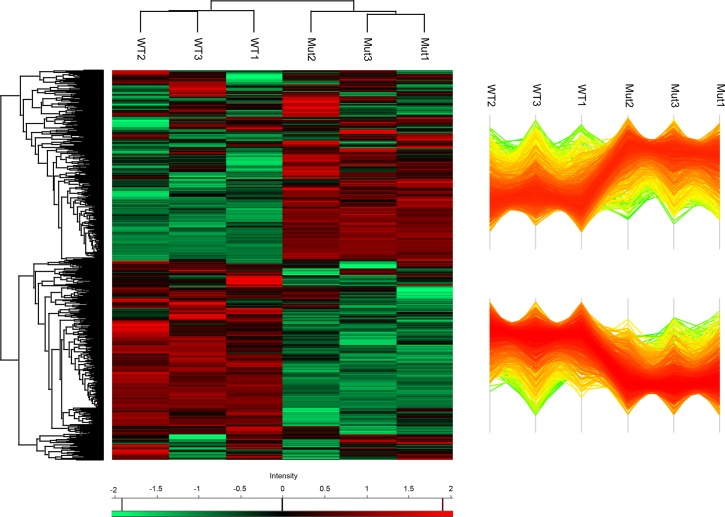
Half of the *L*. *pneumophila* proteins are differentially expressed upon CsrA deletion. Protein intensities in the wt and *csrA*^*-*^ strains (three biological replicates, *csrA*^*-*^ = Mut1-3; wt = WT1-3) were measured by differential shotgun proteomics and visualized in a heat map (left) and a profile plot (right) after non-supervised hierarchical clustering. Every row represents a quantified protein (n = 1448) for which the normalized (LFQ) intensity in each biological replicate is color indicated in the columns.

To assess the influence of CsrA on gene expression, we compared the transcriptome and the proteome profile of the wt and the *csrA*^*-*^ strain during exponential growth phase using whole genome microarrays [9] and mass spectrometry-based shotgun proteomics (LC-MS/MS). Transcriptome analyses revealed that 431 genes showed different RNA levels due to the loss of CsrA, of which 236 were significantly upregulated and 195 significantly downregulated in the *csrA*^*-*^ strain as compared to the wt strain, when a 1.5 fold change in gene expression was taken as cut-off (**S1A Table**). In contrast, at post-exponential growth only 6 genes showed a significant different transcript level between the wt and *csrA*^-^ strain (**S1B Table**). Interestingly, among them are *csrA* (upregulated) and the three small ncRNAs *rsmX*, *rsmY* and *rsmZ*, (downregulated), giving a first indication that CsrA may be involved in controlling its expression. The proteome analysis by LC MS/MS of the mutant and the wt in E phase identified 1662 proteins in total of which 1448 could be quantified. Cluster analysis showed that expression of 1353 out of the 1448 proteins was affected by the mutation of CsrA with about half of the identified proteins up- and half downregulated (**Fig 2**). About 15% of the proteome (216 proteins) was strongly affected with 131 proteins significantly up- and 85 significantly downregulated in the *csrA* mutant (**S2 Table**).

One of the most striking differences between the wt and the *csrA*^*-*^ strain is the timing of flagella expression as demonstrated by flagelline expression already in E phase (**S1C Fig**). Furthermore the loss of CsrA leads to motility of the bacteria already in E phase as judged by microscopy. Indeed, 32 genes of the flagellar biosynthesis gene cluster were significantly up-regulated in the *csrA*^*-*^ strain already in E phase. Among those are FlaA (*lpp1294*), the sigma factor FliA (*lpp1746*) and the response regulator FleR (*lpp1726*) (**S1 Table**). FlaA is the most abundant structural protein of the *L*. *pneumophila* flagellum, whereas FliA and FleR, together with RpoN (*lpp0542*) and FleQ (*lpp0915*), are the master regulators for flagellar assembly [30]. In line with these results, the major regulator of flagella biosynthesis FleQ together with other flagella proteins was also significantly up-regulated in our proteomic data (**S2 Table**). Thus our results confirm that CsrA impacts flagella biosynthesis negatively during replication and shows that CsrA-mediated repression must be relieved to activate motility.

Similar to *E*. *coli* and other bacteria where CsrA was studied in more detail, our microarray and proteomic data showed that CsrA regulates the central carbon flux in *L*. *pneumophila*. CsrA positively impacts the pyruvate/2-oxoglutarate dehydrogenase complex (*lpp1515*-*lpp1517*) and proteins that participate in the Entner-Dudoroff (*lpp0483*-*lpp0487*) pathway, an alternative pathway to catabolize glucose to pyruvate. Additionally, the transcript of the glycolysis protein triosephosphate isomerase (*lpp2838*) is down-regulated in *csrA*^*-*^ strain. Furthermore, the ribose-5-phosphate isomerase A (*lpp0108)* of the pentose phosphate pathway, the pyruvate dehydrogenase complex (*lpp1461*) and the 2-oxoglutarate dehydrogenase (*lpp0597*) of the TCA cycle were down-regulated at the protein level in absence of CsrA (**S1 and S2 Tables**).

In late stages of the infectious cycle *L*. *pneumophila* contains poly-β-hydroxybutyrate (PHB) granules [31, 32]. PHB is an important carbon and energy storage polyester produced under nutrient-limited conditions in numerous microorganisms [33]. The regulation of this pathway in *L*. *pneumophila* is not known. We found that two genes that are part of the poly-3-hydroxybutyrate biosynthesis pathway, the acetoacetyl-CoA reductase (*lpp0621*) and the 3-hydroxybutyrate dehydrogenase (*lpp2264*, *bdhA*) are induced on transcript and protein level in the absence of CsrA. Expression of *bdhA* was shown to be dependent on RpoS [34]. As RpoS is slightly up regulated in the *csrA*^*-*^ additional, indirect influence of RpoS cannot be excluded. Thus, CsrA seems to play a vital role in regulating the carbon metabolism in particular for the decision whether pyruvate is metabolized for energy and metabolite production or if it is transferred into its storage compound.

Finally, a prominent feature of a *csrA*^-^ mutant is its reduced intracellular growth **(Fig 1).** The reason for this phenotype is clearly reflected in the transcriptome and proteome data, as at least 40 substrates of the Dot/Icm secretion system are differentially expressed between the wt and the *csrA*^*-*^ strain (**S1 and S2 Tables**). Among them the Sid family effectors SidE, SidJ, SidK, SdbC, SdeD, of which *e*.*g*. the SidE family proteins have recently be shown to ubiquitinate multiple Rab small GTPases associated with the endoplasmic reticulum [35, 36]. Furthermore, the expression of the effectors RavA, RavH, MavH, MavT, MavQ, LepA, LepB, VipE, PieF and YlfB is influenced by CsrA. For some of these (*e*.*g*. RavH, MavT, MavQ, YlfB) it has been shown previously that their expression is under the direct control of the LetA/Rsm/CsrA regulatory cascade and that the corresponding transcripts might contain CsrA-regulatory elements mainly in the 5'UTR/RBS regions suggesting a regulation by CsrA [16, 25]. Eighteen of the Dot/Icm substrates differentially expressed in the transcriptome were also differentially expressed in the proteome (**S1 and S2 Tables**). An example is the eukaryotic-like sphingosine-1-phosphate lyase (*Lp*SPL, Lpp2128) that targets the sphingolipid metabolism of the host cell to restrain autophagy [37]. Taken together, genome wide transcriptome and proteome analyses showed that CsrA activity has a major impact on motility and the central carbon metabolism. Furthermore an important role of CsrA in virulence and stress response was clearly seen by the differential expression of many Dot/Icm secreted proteins and the intracellular replication defect of a the *csrA*^*-*^ strain.

### RIPseq captures genome-wide target-RNAs directly regulated by CsrA

Our proteome and transcriptome data show that CsrA influences the expression of many major regulatory proteins like FleQ and FleR, the alternative sigma factor RpoS (*lpp1247*), the nucleoid-associated proteins Fis2 (*lpp1324*), Fis3 (*lpp1707*) and HU-beta (*lpp1826*) or the transmission trait enhancer protein LetE. As it is well known, transcriptome and proteome data overlap only partially and they do not allow distinguishing between direct or indirect regulations. Thus we analysed the direct interaction between CsrA and its target-RNAs by co-immunoprecipitation experiments followed by massive sequencing (RIPseq). Five independent RIPseq libraries obtained with epitope-tagged CsrA protein were created and deep sequencing of CsrA-bound transcripts using an Illumina platform was performed. We identified in total 479 RNAs localized in the untranslated (UTR) and/or coding regions of mRNAs or of known non-coding sRNAs of *L*. *pneumophila* (**S3 Table**). To identify CsrA targets, we used a script developed by Dugar and colleagues [38], that calculates in sliding windows the coverage enrichment of the co-IP versus the control. For the comparison, the coverage files were normalized according to the number of mapped base pairs of each sample (control and co-IP). A peak was defined as an at least five times sequence enrichment in the co-IP as compared to the control IP. The values for the enrichment of each CsrA target are recorded in **S3 Table**. Calculation of the enrichment of A(N)GGA motifs in the CsrA target peaks defined in the co-IP as compared to those found in the control IP revealed a 1.24 to 2.94 enrichment of GGA motifs in the peaks of the co-IP **(S4 Table**). Epitope tagged CsrA was expressed about 2 times more than the native copy (**S2 Fig**) thus few RNAs that were bound at low affinity, but are not true targets might have been included. When comparing results obtained from the protein and transcriptome data the correlation factor was R^2^ = 0.314, a factor comparable with data from other bacteria where a correlation of 0.20–0.47 was reported [39]. The RIPseq, transcriptome and proteome data combined showed a concordance of 32%. **S5 Table** reports the 51 targets that were identified in all three approaches. **Table 1**summarizes the distribution of the proteins encoded by these CsrA targeted RNAs according to their functional category and **Table 2**shows all targets that are discussed here in detail. As suggested from the RIPseq, proteome and transcriptome data combined, CsrA affects all major metabolic pathways like the carbon, amino acid and fatty acid metabolism as well as energy transfer, transport and uptake of nutrients or response. Furthermore, the mRNA of many proteins related to the biosynthesis of cofactors, vitamins and secondary metabolites including thiamine, pyridoxal, inositol phosphate, S-adenosylmethionine or riboflavin are directly interacting with CsrA (**Table 1**and **S3 Table**). Secondly, CsrA seems to have a major influence on translational and transcriptional processes as well as DNA replication and repair as judged from its interaction with numerous RNAs of proteins of these functional groups. Thirdly, CsrA directly binds to RNAs of proteins implicated in virulence, stress response and adaptation to environmental changes (**Table 1**and **S3 Table**).

**Table 1:** Summary of RNA targets of CsrA identified by using Co-immunoprecipitation with Flag-tag antibodies and subsequent deep sequencing (RIPseq).

	Pathways/function	Number of target RNAs
1	Carbohydrate-Metabolism and Energy	38
2	Aminoacid-Metabolism, other aminoacids	28
3	Nucleotide Metabolism	17
4	Fatty acid/Lipid-Metabolism, Butanate/Propanoate	34
5	Transcription, RNA Stability; Translation	46
6	Regulation	16
7	Cell Envelope, Cell Division, Motility	27
8	Protein Secretion/Trafficking, Protein Fate	41
9	Cofactors and Vitamins, Secondary Metabolite	25
10	Transport, Uptake	21
11	DNA Replication, Recombination and Repair	17
12	Virulence Factors	48
13	Stress Response, Defence; Xenobiotica	25
14	Unknown, Hypothetical Proteins; Others	91
15	Small RNAs	5

**Table 2:** Summary of the genes influenced by CsrA and discussed in detail.

Gene	Description	RIPseq	Transc.	Prot.
**Regulation of motility**				
*lpp0915*	FleQ, Transcriptional regulator	14.5	/	2.66
*lpp1726*	FleR, Response regulator	49.18	2.27	/
*lpp1294*	FlaA, Flagellin	/	2.88	14.2
**Regulation of quorum sensing**				
*lpp2788*	LqsR, Response regulator	16.47	/	/
**Regulation of virulence *via* global regulators and Dot/Icm effectors**				
*lpp1247*	RpoS, RNA polymerase sigma factor	62.5	1.80	/
*lpp1255*	PmrA, TCS response regulator	25.68	/	/
*lpp0606*	Fis1, Global DNA-binding transcriptional regulator	51.2	/	/
*lpp1324*	Fis2, Global DNA-binding transcriptional regulator	50.83	3.42	4.52
*lpp1707*	Fis3, Global DNA-binding transcriptional regulator	15.42	0.35	0.47
*lpp1826*	HU-beta, DNA-binding protein	5.52	1.78	2.84
*lpp1413*	RelA, GTP pyrophosphokinase	25.75	/	0.62
*lpp1002*	LidA, Dot/Icm effector protein	25.23	1.88	/
*lpp1033*	Ectonucleoside triphosphate diphosphohydrolase	10.61	/	/
*lpp2128*	*Lp*Spl, Eukaryotic-like sphingosine-1-phosphate lyase	21.69	0.29	0.45
*lpp2246*	YlfA, Dot/Icm effector protein	27.46	2.11	/
*lpp0982*	MavT, Substrate of the Dot/Icm secretion system	27.90	1.53	2.68
*lpp3047*	MavQ, Substrate of the Dot/Icm secretion system	19.77	1.54	1.67
**Regulation of pyruvate metabolism**				
*lpp0153*	Gap, Glyceraldehyde 3-phosphate dehydrogenase	37.40	/	/
**Regulation of energy/metabolite production**				
*lpp2264*	3-hydroxybutyrate dehydrogenase	14.34	1.93	3.16
*lpp0620*	Acetoacetyl-CoA reductase	9.94	/	3.52
*lpp0621*	Acetoacetyl-CoA reductase	/	/	2.76
*lpp2038*	PhbC, Polyhydroxyalkanoate synthase	74.39	/	/
*lpp1461*	Pdh, Pyruvate dehydrogenase complex	46.09	/	0.65
*lpp0483*	Glucose-6-phosphate 1-dehydrogenase	12.91	0.55	/
*lpp0485*	6-phosphogluconate dehydratase	23.40	0.47	/
*lpp0728*	Acetoacetate decarboxylase	113.00	3.84	2.60
*lpp0108*	Ribose-5-phosphate isomerase A	18.08	/	0.62
*lpp1516*	Pyruvate/2-oxoglutarate dehydrogenase complex	18.92	0.62	/
*lpp2838*	Tpi, Triosephosphate isomerase	9.46	0.48	/
*lpp2931*	RNA pyrophosphohydrolase	69.62	/	0.53
*lpp0597*	SucA, 2 -oxoglutarate dehydrogenase E1 subunit	38.36	/	0.65
*lpp0986*	Alanine dehydrogenase	49.88	0.63	/
*lpp0535*	Fba, fructose-bisphosphate aldolase	42.9	/	/
*lpp2020*	Eno, Enolase	12.3	/	/
**Regulation of thiamine biosynthesis**				
*lpp1522*	NMT1/THI5-like protein (TPP riboswitch)	27.77	/	/
**Regulation of iron homeostasis**				
*lpp0438*	Fur, Ferric uptake regulation protein	16.04	/	/
*lpp0236*	PvcA, Pyoverdine biosynthesis protein	8.76	/	/
*lpp0252*	KatG, Catalase/peroxidase	19.68	/	/
*lpp0288*	Heme oxygenase	36.00	0.54	/
*lpp2018*	Zinc/iron transport protein	23.82	/	/
*lpp2164*	Hbp, Heme-binding protein	11.06	0.24	/
*lpp0651*	Fe-S cluster assembly SUF, transcriptional regulator	5.55	/	/
*lpp1898*	4FE-4S binding protein	19.44	/	/
*lpp0854*	L-serine dehydratase, iron-sulfur-dependent	23.86	/	/

Trans; transcriptome analyses wt vs *csrA*^*-*^ strain, Prot, proteome analyses wt vs *csrA*^*-*^ strain, Numbers indicate the fold change (cut off 1.5x for proteome and transcriptome experiments, 5 times enrichment of the peaks in the co-Ip vs control IP for the RIPseq analyses).

### CsrA regulates motility by directly targeting the FleQ and FleR mRNAs

**Fig 3:**
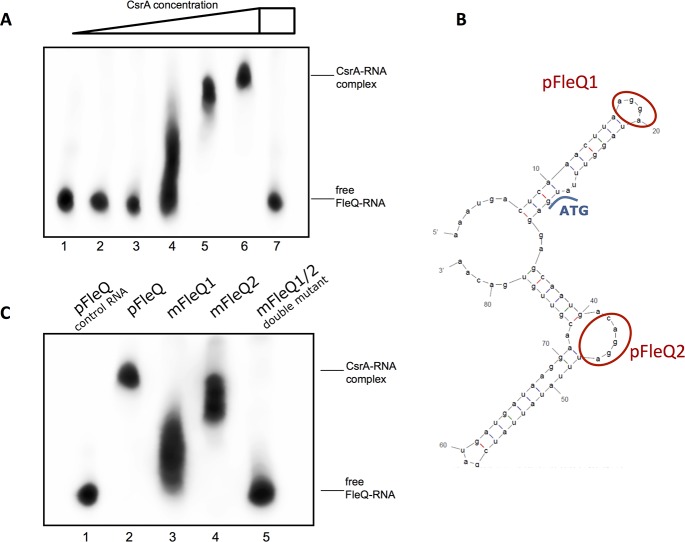
CsrA interacts directly with *fleQ*-mRNA *in vitro*. **A**) Electromobility shift assays (EMSA) with 200nM of biotinylated ***fleQ*-mRNA** combined with varying concentrations of purified CsrA-His were undertaken in a 6% Native Tris-PAGE gel. Lane 1: no CsrA, lane 2: 0.2 μM CsrA, lane 3: 0.5 μM CsrA, lane 4: 1.0 μM CsrA, lane 5: 2.0 μM CsrA, lane 6: 5.0 μM CsrA, lane 7: 5.0 μM CsrA + 2.0 μM unlabeled RsmZ. **B**) Mfold secondary structure prediction of the ***fleQ*-mRNA** fragment used for the EMSA. Red, two potential CsrA-binding sites, which are mutated in C. Blue, transcriptional start codon. **C**) EMSA with recombinant CsrA and 200 nM of non-mutated RNA (pFleQ) or mutated in the indicated regions (mFleQ). AGGA motifs were replaced by an AAAA sequence using PCR mutagenesis. Lane 1: no CsrA + non-mutated ***fleQ*-mRNA**, lane 2: 5.0 μM CsrA + non-mutated ***fleQ*-mRNA**, lane 3: 5.0 μM CsrA + m***fleQ*-mRNA** mutated in region 1, lane 4: 5.0 μM CsrA + m***fleQ*-mRNA** mutated in region 2, lane 5: 5.0 μM CsrA + m***fleQ*-mRNA** mutated in both region 1 and 2.

RIPseq analyses identified two RNAs directly bound to CsrA, one located in the 5’UTR of the mRNA coding for the transcriptional regulator FleQ and the other one in that coding for the two-component response regulator FleR. Both regulators are indispensible for flagella biosynthesis in *L*. *pneumophila* as the deletion of one or the other led to the down regulation of all flagellar genes and consequently to a complete loss of motility [30]. Indeed, EMSA assays confirmed the interaction of the *fleQ* mRNA with CsrA *in vitro*, and the addition of an excess of unlabeled RsmZ as control, abolished this interaction (**Fig 3A**). To identify the binding sites of CsrA we predicted the RNA structure with the Mfold program [40]. This revealed two A(N)GGA-binding motives in the *fleQ* mRNA that are present in loops of (**Fig 3B**). To analyze whether these were indeed the CsrA binding sites, we mutated the FleQ1 AAGGA-loop motif to AAAAA and the FleQ2 AGGA-loop motif to AAAA (**Fig 3B**). The mutation of the FleQ2 motif had only little consequences whereas mutation of FleQ1 led to a partial loss of the interaction. However, mutation of both sites completely abolished binding of CsrA (**Fig 3C**). The existence of two binding sites might reflect an independent binding of one or two CsrA proteins to the FleQ-mRNA with different affinities or could also indicate a serial interaction of the homodimeric CsrA with both loops initiating at FleQ1. The motif is overlapping with the ribosomal binding site (RBS) of the *fleQ* gene, indicating that CsrA has a negative effect on FleQ translation by preventing ribosome binding, which is the most predominant operational mode of CsrA [12, 13]. This result was further substantiated by the result of BlaM reporter assays where the potential CsrA-binding region identified by RIPseq was fused upstream of the BlaM gene (**S3A Fig**). Indeed, in absence of CsrA or mutations of the CsrA-binding motifs resulted in a higher ß-lactamase activity due to higher expression of the BlaM protein (**S3A Fig**). Furthermore, this results are in agreement with the phenotypic observation that flagella biosynthesis is dependent on CsrA and our proteomic data, in which FleQ as well as FleR are significantly up-regulated in the *csrA*^-^ strain (**S2 Table**). Thus, the regulatory function of CsrA seems to be exerted upstream the regulatory cascade for flagellar biosynthesis by directly preventing the efficient translation of FleQ and FleR during replication.

### CsrA regulates quorum sensing by directly binding to the LqsR mRNA

**Fig 4:**
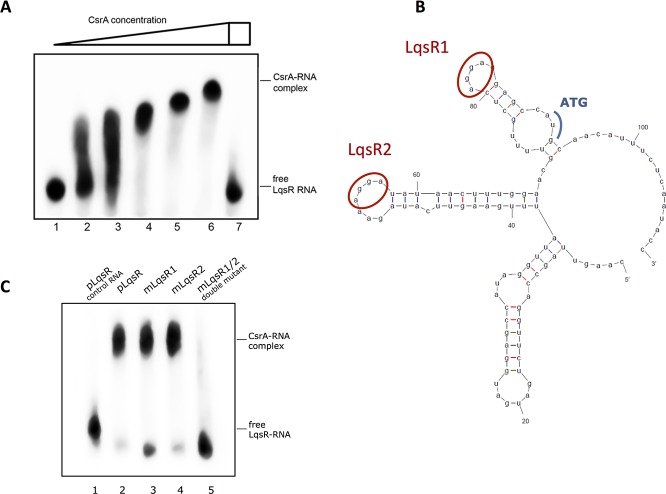
CsrA interacts directly with *lqsR* mRNA *in vitro*. **A)** Electromobility shift assay (EMSA) with 200nM of biotinylated *lqsR* mRNA combined with varying concentrations of purified CsrA-His *lqsR* mRNA and recombinant CsrA in 6% Native Tris-PAGE. Lane 1: no CsrA, lane 2: 0.2 μM CsrA, lane 3: 0.5 μM CsrA, lane 4: 1.0 μM CsrA, lane 5: 2.0 μM CsrA, lane 6: 5.0 μM CsrA, lane 7: 5.0 μM CsrA + 2.0 μM unlabled RsmZ. **B)** Mfold secondary structure prediction of the *lqsR* mRNA fragment used for the EMSA. Red, two potential CsrA-binding sites, which are mutated in C. Blue, transcriptional start codon. **C**) EMSA with recombinant CsrA and 200 nM of non-mutated RNA (pLqsR) or mutated in the indicated regions (mLqsR). AGGA motifs were replaced by an AAAA sequence using PCR mutagenesis. Lane 1: no CsrA + non-mutated *lqsR* mRNA, lane 2: 5.0 μM CsrA + non-mutated *lqsR* mRNA, lane 2: 5.0 μM CsrA + m*lqsR* mRNA mutated in region 1, lane 3: 5.0 μM CsrA + m*lqsR* mRNA mutated in region 2, lane 4: 5.0 μM CsrA + m*lqsR* mRNA mutated in both region 1 and 2.

*L*. *pneumophila* produces a quorum sensing autoinducer molecule, 3-hydroxypentadecane-4-one (LAI-1), that is synthesized by LqsA and sensed by the two sensor kinases LqsS and LqsT, which subsequently phosphorylate the response regulator LqsR [20, 41, 42]. Expression of LqsR was shown to be dependent on RpoS and LetA [22]. LqsR-knock out mutants are defective in the formation of the *Legionella* containing vacuole (LCV) and in the replication in amoeba and macrophages [23]. Here we show that LqsR is linked to the LetA/Rsm-regulatory cascade through the binding of CsrA to the RBS region of LqsR (**Fig 4A)**. A clear band shift in presence of increasing concentrations of purified CsrA was observed whereas adding a surplus of unlabeled RsmZ outcompeted the interaction between CsrA and the biotin-labeled *lqsR*-mRNA (**Fig 4A)**. Mfold analyses predicted two binding loops containing an A(N)GGA regulatory motif. Mutating one of these motives had nearly no influence on CsrA-binding whereas the mutation of both led to a complete loss of the interaction of CsrA with the 5’UTR region of the LqsR mRNA (**Fig 4B**). This could be the consequence of independent binding of CsrA to one or the other motif, or analogous to FleQ, might indicate a cooperative binding to both loops that would act equivalently in this case. Yet, both binding sites together might not be necessary to ensure efficient CsrA interaction, but may increase the affinity of CsrA to the LqsR mRNA. We also undertook BlaM reporter assays that showed that the mutation of the CsrA-binding loci led to elevated BlaM expression (**S3B Fig**) hence further supporting our model in which CsrA negatively regulates the LqsR expression most likely on the translational level. Thus the two major regulators CsrA and LqsR act complementary, with CsrA governing the transition to transmissive phase whereas LqsR facilitates the switch from transmissive phase back to replicative phase. This direct connection between CsrA and LqsR uncovers the missing link between the stringent response pathway and the response to local population density via quorum sensing.

### CsrA regulates the switch to virulent *Legionella* by targeting global regulators, Dot/Icm effectors and autoregulation of the stringent response

**Fig 5:**
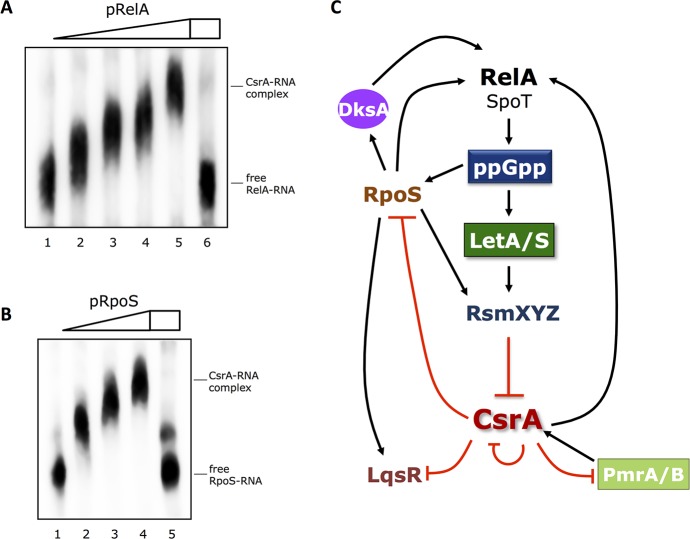
CsrA feedback regulation and autoregulation of the stringent response. **A**) Electromobility shift assay (EMSA) with 200nM of biotinylated RNA demonstrating the interaction of purified CsrA and *relA* mRNA *in vitro*. 200nM of biotinylated *relA* mRNA and increasing concentrations of recombinant CsrA in 6% Native Tris-PAGE were used. Lane 1: no CsrA, lane 2: 0.5 μM CsrA, lane 3: 1.0 μM CsrA lane 4: 2.0 μM CsrA, lane 5: 5.0 μM CsrA, lane 6: 5.0 μM CsrA + 2.0 μM unlabeled RsmZ. **B**) EMSA with 200nM of biotinylated RNA demonstrating the interaction of purified CsrA and *rpoS* mRNA *in vitro*. 200nM of biotinylated *rpoS* mRNA and increasing concentrations of recombinant CsrA in 6% Native Tris-PAGE were used. Lane 1: no CsrA, lane 2: 1.0 μM CsrA, lane 3: 2.0 μM CsrA, lane 4: 5.0 μM CsrA, lane 5: 5.0 μM CsrA + 2.0 μM unlabeled RsmZ. **C**) Model of the stringent response and quorum sensing network in *L*. *pneumophila* and the role of CsrA on their regulation. During the transmissive phase, amino acid and fatty acid starvation triggers the GTP pyrophosphokinase RelA and the ppGpp synthetase/hydrolase SpoT to produce the alarmone (p)ppGpp. Amongst others, the (p)ppGpp production results in a higher transcription rate of the small ncRNAs RsmX, RsmY and RsmZ which dissociate the RNA-binding protein CsrA from its target-RNAs. This leads to an activation of RpoS, LqsR and PmrA expression (positive feedback) that were formerly repressed by CsrA and an inhibition of RelA (negative feedback). Predicted negative effects in the regulatory cascade are represented by red lines, positive effects by black arrows.

Strikingly, in our RIPseq analyses we identified RelA (Lpp1413), RpoS (Lpp1247), PmrA (Lpp1255) and LqsR (Lpp2788) as targets of CsrA. These four regulators have been shown to be major players in the switch from replicative to transmissive/virulent *L*. *pneumophila* [15, 16, 23, 24]. Indeed, *in vitro* interaction assays with CsrA and the mRNAs of LqsR, RpoS and RelA, respectively confirmed the interaction of these mRNAs with CsrA (**Fig 5A and 5B)**. The putative CsrA-binding sites for these regulators were all located in the translation initiation region suggesting a negative regulation of the translation, a model that is in line with the results of the BlaM reporter assays for LqsR and RpoS (**S3B and S3C Fig**). Surprisingly we identified in our proteomic analyses RelA down-regulated in the *csrA*^-^ mutant (**S2 Table**), a finding that was also supported by the results of the BlaM reporter assay (**S3D Fig**). Thus although, the CsrA-binding site is overlapping the RBS/start codon of RelA, we cannot exclude that the presence of CsrA might have also an auxiliary effect on RelA translation. However, due to the complexity of the network and the fact that CsrA directly regulates the expression of a high number of major regulators in *Legionella*, like LetE (**S4 Fig**), the three Fis proteins, HU-beta or RpoH (**Table 2**and **S3 Table**), also secondary regulatory effects are captured by our proteomic data. Additionally, we discovered that CsrA might bind also to its own mRNA. To test whether CsrA might also bind to the mRNA of Hfq, another major RNA binding protein of *L*. *pneumophila* that was not identified in our RIPseq data but whose mRNA contains also GGA motifs, we tested *in vitro* binding by EMSA assays. In accordance with our RIPseq results, no interaction was found, indicating that CsrA is not influencing the Hfq regulation regulatory pathways (**S5 Fig**). Taken together, our data suggest that several feedback loops coordinate the different signals derived from the stringent response and from quorum sensing and an auto-regulation to fine-tune CsrA activity itself exists in *L*. *pneumophila* **(Fig 5C).**

Moreover, CsrA regulates the switch to virulent *L*. *pneumophila* not only by targeting the major regulators, but also by directly targeting secreted effector proteins. In total, 41 of the known Dot/Icm effectors are directly targeted by CsrA according to our RIPseq analyses of which 32 are also found to be differentially expressed in the transcriptome and/or proteome approach (**S1, S2 and S3 Tables**). To substantiate this finding, we tested the interaction of CsrA with the mRNAs of three of them by EMSA analyses (**S6 Fig**). This confirmed that the eukaryotic ectonucleoside triphosphate diphosphohydrolase (ecto-NTPDase) (Lpp1033) required for optimal intracellular replication [43], LidA, (Lpp1002) implicated in Rab1 sequestration and development of the LCV [44], and YlfA (Lpp2264) that inhibits endosomal trafficking [45, 46], are all directly targeted as purified CsrA showed clear binding *in vitro* to the corresponding YlfA-, LidA- and NTPase-RNAs (**S6 Fig**). Interestingly, as judged from the combined approach of total protein mass spectrometry analyses and RIPseq experiments, the regulatory effect of CsrA is not only repressive. For example the Dot/Icm secreted eukaryotic-like sphingosine-1-phosphate lyase *Lp*Spl [37] and others are downregulated in the *csrA*^*-*^ strain in our proteome and transcriptome dataset (**S1 and S2 Tables**), indicating that the effect of CsrA allows a highly coordinated life cycle regulation in a spatial and timely manner. Taken together, CsrA regulates virulence formation *via* two routes, first by regulating several major regulatory proteins and secondly by directly interacting with the transcript of over 40 Dot/Icm-secreted effector proteins.

### CsrA regulates the pyruvate metabolism by preventing Rho-dependent termination and inducing transcriptional polarity

**Fig 6:**
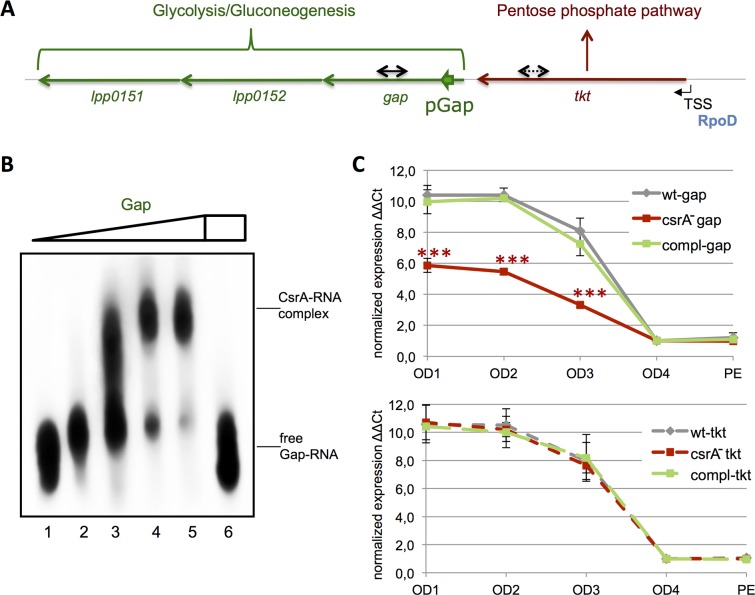
Glyceraldehyde 3-phosphate (Gap) and transketolase (Tkt) transcription is regulated differently by CsrA. **A**) Schematic organization of the PPP/Glycolysis-operon in *L*. *pneumophila* Paris. TSS indicates the transcriptional start site under the control of an RpoD-dependent promoter. Green, bold arrows show the CsrA-binding region and black arrows highlight the region where qRT-PCR was conducted. **B**) EMSA with 200nM of biotinylated RNA demonstrating the interaction of purified CsrA and *gap* mRNA: Lane 1: no CsrA, lane 2: 0.5 μM CsrA, lane 3: 1.0 μM CsrA, lane 4: 2.0 μM CsrA, lane 5: 5.0 μM CsrA, lane 6: 5.0 μM CsrA + 2.0 μM unlabled RsmZ. Right side, run-off transcript produced under optimal *in vitro* transcription conditions performed with the MEGAshortscript Kit (ambion) to show transcript length compared to the low range ssRNA ladder (NEB). **C**) qRT-PCR results of the *gap* and the *tkt* transcripts at different growth stages (OD) between wt and *csrA*^-^ show lower expression levels of the *gap* gene in E-phase (OD1-3) in absence of CsrA whereas *tkt* is not affected. No differences are noticed during transition (OD3) and PE-phase. Complementation of the *csrA*^-^ strain restored the wt transcript levels

Another CsrA-binding site was located within the operon *lpp0151-lpp0154*, encoding the transketolase (Lpp0154, Tkt), the glyceraldeyd 3-phosphate dehydrogenase (Lpp0153, Gap), the phosphoglycerate kinase (Lpp0152*)* and the pyruvate kinase (Lpp0151) one of the rate determining enzymes of the glycolysis (**Fig 6A**). The transcriptional start site (TSS) of this operon lies upstream of *lpp0154* as determined by transcriptional start site mapping using dRNAseq [18]. Even though CsrA-binding was seen at several distinct regions inside the operon, the most significant site was located at the 5’ region of the *gap*-mRNA (**Fig 6A** and **S3 Table**). We confirmed binding of CsrA to this site by EMSA assays with *in vitro* transcribed mRNA comprising this region and purified CsrA (**Fig 6B**). Interestingly, in contrast to the targets discussed above, the CsrA-binding site A(N)GGA was not associated with the ribosome-binding region suggesting a regulatory mechanism different from translation hindrance.

**Fig 7:**
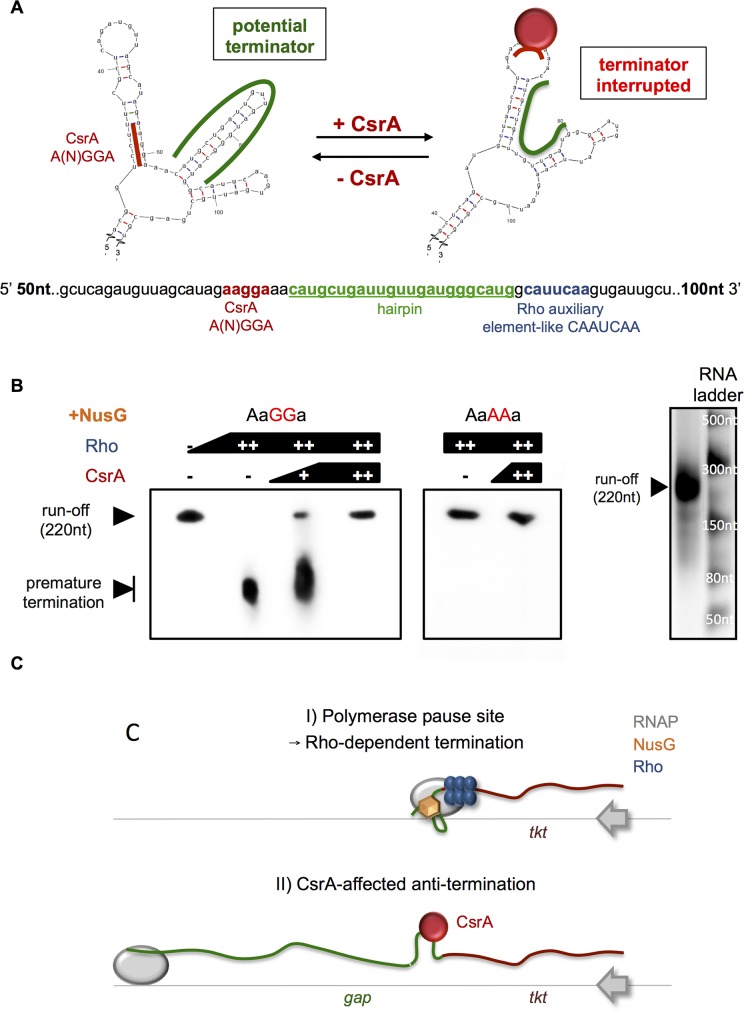
CsrA acts as a positive regulator for Glyceraldehyde 3-phosphate (Gap) by preventing premature transcriptional termination of the PPP/Glycolysis-operon. **A**) RNA secondary structure Mfold-prediction of the CsrA-binding region inside the *gap* gene reveals two major conformations: the left one contains a potential hairpin-terminator while the A(N)GGA-motif is covered in a double-strand region with low affinity to CsrA. The right one, shows the A(N)GGA-motif located in an open loop with high CsrA-interaction affinity and the hairpin structure is disrupted. Below, the nucleic acid sequence is shown that was used for Mfold modeling and the transcription termination assays. Red, CsrA-binding site A(N)GGA; green, the potential transcription terminator hairpin; blue, the putative auxiliary element of Rho-dependent termination. **B**) Left panel: *In vitro* transcription termination assay in presence of 1 μM of purified NusG-protein and varying concentration of Rho- and CsrA-protein (+ 0.5 μM, ++ 1μM; Lane 1: no Rho, no CsrA, lane 2: 1 μM Rho, no CsrA, lane 3: 1 μM Rho, 0.5 μM CsrA, lane 4: 1 μM Rho, 1 μM CsrA, lane 5: 1 μM Rho, no CsrA, lane 6: 1 μM Rho, 1 μM CsrA). A representative 10% urea-PAGE gel shows the formation of the truncated transcript from the Rho-dependent termination without CsrA and the full-length transcript with CsrA. Right panel: *In vitro* transcribed of the run-off fragment and the marker showing the size of the fragment. **C**) Regulatory model of the transcription of the PPP/Glycolysis operon. In absence of CsrA, Rho-dependent termination within the operon is responsible for polarity effects downstream of the transcriptional block. This leads to reduced transcript-levels of the *gap* gene whereas the *tkt* gene is not affected. When CsrA binds to the RNA, an anti-terminator structure is favored preventing that the elongation complex stalls at the hairpin structure. As a consequence, only the presence of CsrA ensures the efficient transcription of the glycolysis/gluconeogenesis genes of the operon.

To get insight how CsrA affects this operon, we performed qRT-PCR and compared the levels of transcription upstream (at the end of the *tkt* gene) and downstream (inside the *gap* gene) of the CsrA-binding region. As shown in **Fig 6C**, the transcript level of *tkt* and *gap* were similar in the wild type strain during the growth in broth, but showed significant differences in the *csrA*^*-*^ mutant, a phenotype that was complemented when reintroducing *csrA*. Interestingly, the relative amount of the Tkt-transcript was identical in the wt and the mutant whereas the transcription of *gap* was significantly lower in absence of CsrA. Mfold analysis of the secondary structure of the RNA region containing the CsrA binding site predicted two energetically favored conformations: a) the A(N)GGA motif was buried in a double-stranded sequence followed by a strong hairpin and b) the CsrA regulatory motif was present in a loop and the subsequent hairpin was destabilized (**Fig 7A**). Furthermore, adjacent to the hairpin we identified a short sequence closely related to an auxiliary element assisting in Rho-dependent termination of transcription [47]. To test our hypothesis that this region might be involved in preliminary transcription termination of the *gap* operon, we performed *in vitro* transcriptional assays in presence of NusG, an additional factor known to facilitate recognition of the termination signal [47, 48]. The full-length transcript appeared in absence of the Rho factor whereas premature termination was observed when purified Rho was added to the reaction (**Fig 7B**). Strikingly, this effect could be gradually reverted by adding increasing concentrations of CsrA (**Fig 7B**). Northern blot analyses of the wt, *csrA*^*-*^ and the complemented *csrA*^*-*^ mutant showed indeed that in the *csrA*^-^ strain more *tkt* transcript is present and less transcript of the complete operon as compared to the wt and the complemented *csrA*^*-*^ mutant strain (**S7 Fig**). Mutation of the CsrA-binding motif abolished that effect, but differently as expected. The introduction of AAAA replacing the AGGA-motif led to complete transcription run-off even in presence of Rho and independent of CsrA. This is probably due to the fact that the mutation energetically disfavors the development of the hairpin by preventing the auxiliary double-stranded region upstream of it. Thus, it stabilizes the conformation in which the termination structure is disrupted 'mimicking' permanent CsrA-binding. However, a double mutated template comprising the AAAA motif together with its complementary part of the dsRNA region (**S8A Fig**) to UUUU resulted in a truncated transcription fragment independent of CsrA (**S8B Fig)**. Surprisingly, due to this double mutation we observe a premature termination already in absence of Rho and CsrA that was not detected under wt condition (Fig 7B). Possibly, the extended nucleotide mutations introduced provoke the stabilization of conformational changes in the RNA structure that leads to a transcriptional interruption even independent of the Rho protein. However, in presence of Rho the termination process is strongly enhanced compared to the absence of Rho. Furthermore, EMSA assays with this RNA showed that the double mutation led to a complete loss of interaction with the CsrA protein (**S8C Fig)**.

It was shown that CsrA is able to remodel the RNA secondary structure in the leader sequence of the *pgaA* gene of *E*. *coli* to promote Rho-dependent transcription termination [48], but here we demonstrate for the first time that CsrA may participate in the negative regulation of transcriptional termination events in bacteria. Our data suggest that CsrA is actively stabilizing RNA anti-terminator conformations inside a coding sequence rather than preventing a premature stop of transcription. This new model of regulation as proposed in **Fig 7C** would lead to an efficient expression of the glycolysis part of the *tkt/gap*-operon only in presence of CsrA. In this model, CsrA-dependent polar transcriptional effects enable *L*. *pneumophila* to regulate the pentose phosphate pathway (transketolase) and the glycolysis combined (when CsrA is present) or individually (when CsrA is absent) according to the needs of the cell even though both pathways share genes in a single operon.

### CsrA is a central player of metabolic switching from energy/metabolite production to storage

Among the targets identified by RIPseq, additional enzymes of the glycolysis (*fba*, *tpi*, *eno*), the gluconeogenesis (*pps*, *ppc*) the pentose phosphate (*rpiA*, *prsA*) and Entner-Doudoroff pathway (*zwf*-operon) or enzymes for the supply of ATP and reducing equivalents through the pyruvate dehydrogenase complex (*pdh*) and the TCA cycle (e.g. *acnA*, *icd*, *sdh/suc*, *sfcA*) were present (**Table 2 and S3 Table**). Thus, results from our combined datasets indicate that CsrA is a critical regulator of the carbon flux from the nutrient source—in *L*. *pneumophila* mainly amino acids like serine and threonine, but also glucose [49, 50]—to obtain energy *via* oxidative phosphorylation. CsrA seems to play a pivotal role in the production of metabolic intermediates and cell components including the interconversion of amino acids or the biosynthesis of nucleotides and fatty acids/lipids essential for efficient replication and cell proliferation (**Table 1 and S3 Table**). Metabolite flux analyses are under way to further substantiate our results obtained from the RIPseq, transcriptome and proteome data.

In contrast, CsrA is hindering the synthesis of storage molecules and short-chain fatty acids according to our microarray and proteomic data. In absence of a functional glycogen biosynthesis pathway, these molecules, in particular the poly-3-hydroxybutanoate (PHB), are used for carbon and energy storage during nutrient starvation [51, 52]. Several enzymes of these pathways were identified in our RIPseq analyses, including the 3-hydroxybutyrate dehydrogenase (Lpp2264), the acetoactetyl-CoA reductase (Lpp0620) and the polyhydroxyalkanoate synthase (Lpp2038 PhbC) (**S3 Table**). For PhbC, the CsrA-binding region was located in the 5’UTR overlapping the RBS and the start codon assuming negative regulation of translation initiation (**S9A Fig**). The EMSA assay undertaken with the *in vitro* transcribed *phbC* region, confirmed interaction of CsrA with the predicted region (**S9B Fig**).

To support our findings, we analyzed the PHB concentration of the wild type and *csrA*^*-*^ strain during different growth stages. Bacteria were treated with BODIPY493/503, a molecule known to stain nonpolar lipids, a method for quantification of cellular PHB in yeast and bacteria [53]. The fluorescence of the different *L*. *pneumophila* strains was measured by flow cytometry at exponential, post-exponential and stationary phase and the percentage of PHB positive cells was determined. We observed lower amounts of cells containing the storage polymer in replicating cells (E phase) and increasing numbers during the transition to transmissive phase. During stationary (S-) phase, the PHB amount dropped again in the wild type most likely due to its utilization for maintenance of vital physiological function (**S9C and S9D Fig**). In contrast to the wt, no changes during the life cycle were observed in absence of CsrA. In particular during E and S phases, the quantity of PHB positive cells was significantly higher in the mutant compared to wt. Therefore, we assume that CsrA affects the blocking of the production of the storage component PHB during replication and its utilization during S phase as suggested by the missing decline in cellular fluorescence. However, four different polyhydroxyalkanoate synthases, but no PHB hydrolyzing poly(3-hydroxybutyrate) depolymerase were identified in the *L*. *pneumophila* genome. Thus, catabolic and anabolic pathways are possibly executed by the same enzymes in a bidirectional manner.

### CsrA affects thiamine biosynthesis through modulation of the TPP riboswitch conformation

Directly related to the energetic status of the cell is the biosynthesis of co-factors and vitamins that are indispensible to ensure an unobstructed flow of metabolites. Several of their biosynthesis pathways are directly affected by CsrA (**Table 1 and S3 Table**). One example is the regulation of thiamine synthesis in *L*. *pneumophila*. The thiamine derivate, thiamine pyrophosphate TPP, acts as a cofactor in the catabolism of sugars and amino acids and is closely linked to the CH-metabolism through the TPP-depending enzymes, like pyruvate dehydrogenase, 2-oxoglutarate dehydrogenase, transketolase and branched-chain 2-oxo acid dehydrogenase [54].

**Fig 8:**
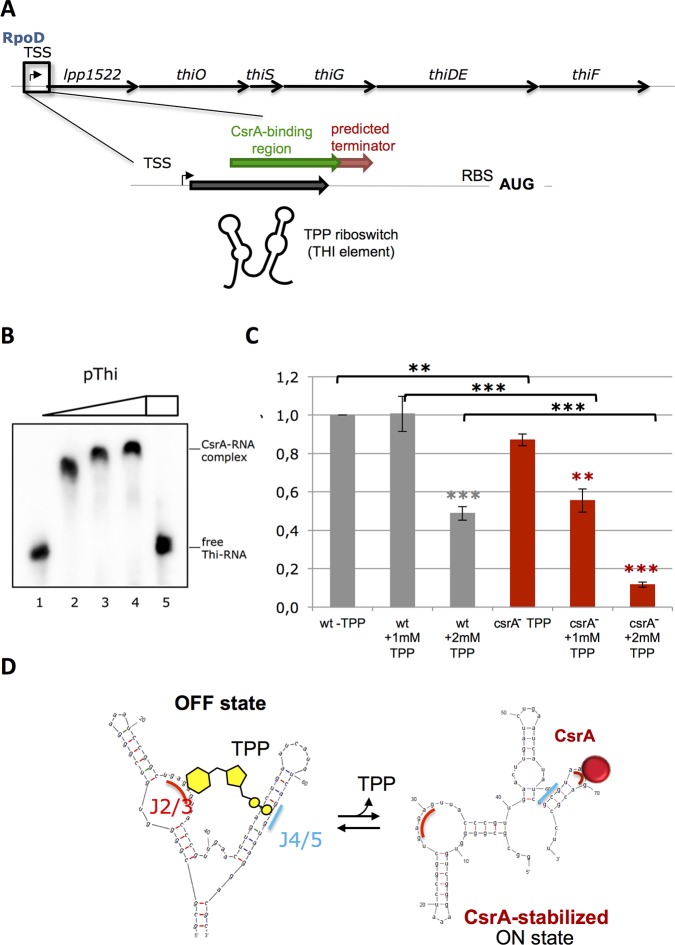
CsrA modulates the expression of a thiamine pyrophosphate (TPP) riboswitch element. **A**) Schematic representation of the *thi*-operon in *L*. *pneumophila* including the transcriptional start site (TSS), the CsrA-binding region, the thi element of the predicted TPP riboswitch and a predicted transcription termination site upstream of the start codon (AUG). The CsrA-binding site is overlapping the *thi* element of the TPP riboswitch. This organization suggests that CsrA is implicated in the fine-tuning of the expression of the downstream *thi*-operon most probably due to conformational changes in the secondary RNA structure. **B**) EMSA with 200nM of biotinylated thi-element (TPP) RNA and purified CsrA: Lane 1: no CsrA, lane 2: 1.0 μM CsrA, lane 3: 2.0 μM CsrA, lane 4: 5.0 μM CsrA, lane 5: 5.0 μM CsrA + 2.0 μM unlabeled RsmZ. **C)** Beta-lactamase (BlaM) assay in minimal medium grown *Legionella* without, with 1 mM and with 2 mM of TPP. BlaM activity in 10μg total protein of wt and *csrA*^*-*^ strain containing the 5'UTR of the *thi*-operon in a pXDC61 plasmid was measured. Each value represents the mean +/- SD of three independent experiments. BlaM activity is significantly decreased in the mutant at the different conditions indicating a positive effect of CsrA on the *thi*-operon expression in *L*. *pneumophila*. **D**) Model of the TPP riboswitch modulated by CsrA. Mfold prediction of the secondary structure of the 5'UTR *thi*-region: When TPP is bound, the OFF state of the riboswitch is favored in which the expression of the operon is inhibited (most likely due to premature termination at the predicted termination site). The presence of CsrA in contrast might stabilize the ON state where the structure of the thi-element is dispersed, hence, higher amounts of TPP would be necessary to shift the element back to the OFF state leading to the down-regulation of the *thi*-genes expression.

We identified a CsrA-binding region upstream the *thi*-operon of *L*. *pneumophila* (*lpp1522-1527*) and a structure with similarity to a THI-box sequence of the TPP riboswitch in the 5'UTR region (**Fig 8A**) that was also identified by RibEx analysis [55]. Additionally, a putative Rho-independent terminator is located directly downstream of the TPP riboswitch **(Fig 8A**). EMSA assays with the *in vitro* transcribed 5'UTR RNA region and purified CsrA showed interaction as a function of CsrA concentration (**Fig 8B**). To study the functionality of the regulatory region, we established a reporter assay by fusing the TPP-riboswitch sequence to the β-Lactamase (BlaM) encoding gene. *L*. *pneumophila* wt *csrA*-mutant containing this plasmid were grown in a minimal medium [56] with and without defined concentrations of thiamine pyrophosphate. In fact, we observed a dependency of BlaM activity on TPP (**Fig 8C**) and that in absence of additional TPP, BlaM activity was significantly reduced in the mutant compared to the wt. This effect was even more pronounced in presence of 1 mM and 2 mM of TPP indicating that indeed CsrA is beneficial for the expression of the *thi*-operon. A similar situation has been described for the *E*. *coli* riboswitch responding to the molybdenum cofactor, as CsrA is able to activate the expression of the corresponding *moaA* genes by binding to the mRNA-leader sequence [57]. To strengthen our observations, we mutated the conserved region of the predicted TPP riboswitch that is known to be indispensible for binding the thiamine moiety of the TPP (**S10A Fig**). Indeed, this mutation led to an uncoupling of the BlaM activity from the extracellular TPP concentration (**S10B Fig**). Whereas mutation of the CsrA-binding (**S10A Fig**) site resulted in a lower BlaM activity already at low TPP concentrations compared to the non-mutated reporter assay (**S10B Fig**) similar to what was observed in the *csrA*^-^ strain.

Our results suggest a model where a concerted response between TPP and CsrA regulates the production of thiamine in *L*. *pneumophila*. As shown in **Fig 8D** two predominant conformational riboswitch-structures are predicted by Mfold. One is the known tandem-loop conformation of the riboswitch, the OFF state, with high affinity to TPP and stabilized by its binding. In this state the operon expression is inhibited as indicated from qPCR analysis (**S11 Fig**), probably by transcriptional termination due to the presence of a terminator sequence in the 5' UTR leader sequence of the *thi*-operon. In the alternative conformation, the predicted ON state, the typical secondary structure of the TPP-riboswitch is unfolded and an alternative, third loop is formed containing the CsrA-regulatory motif A(N)GGA. We postulate that this structure has less affinity to TPP and diminishes the premature termination of the *thi*-leader sequence. Additionally, interaction with CsrA to the newly-formed A(N)GGA-loop is able to stabilize this open structure and shifts the reaction balance to the right even in presence of moderate concentrations of TPP.

This thiamine dependent riboswitch is the first riboswitch identified in *L*. *pneumophila* and is another example for the diversified functionality of CsrA in the bacterial cell beyond translational interference. We assume that a CsrA-dependent fine-tuning mechanism of the TPP riboswitch ensures sufficient production of thiamine in the cell during high metabolic activity by altering the threshold of the TPP inhibitory feedback regulation.

### CsrA regulates iron homeostasis inter alia through stabilization of the *fur* mRNA

To deprive pathogens from the availability of iron, the infected host cells go into an “iron-withhold defense mode” leading to a cross-regulatory interaction between iron homeostasis and the immune response [58]. Therefore pathogens have developed many mechanisms to optimize iron acquisition from the host cell. Iron-uptake in *L*. *pneumophila* comprises iron-chelating siderophore production and ferrous iron uptake systems [59]. However excess of iron can be toxic for the cells because of the high reducing potential and the generation of reactive oxygen species. Therefore, a tight control of the intracellular iron concentration is inevitable. The uptake is coordinated in most bacteria by the ferric uptake regulator Fur and an iron-responsive regulatory sRNA [60]. Here we found that CsrA interacts with the transcript of the *fur* gene suggesting a growth phase-dependent control of iron acquisition. This is in agreement with the previous finding that iron starvation stimulates virulence formation, motility and stress resistance in *L*. *pneumophila* [61]. Interestingly, the observed CsrA-binding sites are located inside the CDS region (**S12A Fig**), but EMSA assays confirmed binding of CsrA to pFur2 under *in vitro* conditions, but not to pFur1, where another potential CsrA interaction region could be located according to the RIPseq analyses (**S12B Fig**). Thus our results suggest that a sole CsrA-binding site inside the Fur-coding sequence is present.

Growth phase-dependent analysis of the Fur transcript by RT-qPCR revealed increased transcription during E phase, which was slightly, but significantly reduced in the *csrA*^*-*^ background (**S12C Fig**). Therefore, CsrA seems to positively influence the *fur* transcript levels in metabolically active cells. To better understand how CsrA may function in this context we analyzed the stability of the Fur-mRNA in presence of rifampicin over time. This revealed a significant reduction of the half-life of the *fur*-mRNA in absence of CsrA *in vivo* and a higher RNA stability when over-expressing CsrA (**S13A Fig**). According to this observation, we predict a stabilizing effect of CsrA on the Fur transcript, similar to what was described in *E*. *coli* for the *glgC* or *flhDC* transcripts [62, 63]. The impact of CsrA on iron acquisition is also seen when growing the wt and the *csrA*^*-*^ strain under different iron conditions. Indeed, loss of functional CsrA led to a growth defect at very low (minimal medium without additional iron) and very high iron concentrations, similar but in a lesser extent as for a *fur*-knock out strain **(S13B Fig**). We further quantified the siderophore secretion capacity of the wt compared to the *csrA*^*-*^ strain using the CAS assay as previously described [61] after transfer from iron-rich to iron-starvation medium. This showed a clear correlation between CsrA and CAS activity suggesting a vital role of CsrA in siderophore production and/or secretion **(S13C Fig).** Furthermore, the RIPseq analysis identified numerous CsrA-RNA interactions with proteins that are directly linked to iron acquisition (pyoverdine biosynthesis protein PucA (Lpp0236), heme oxygenase (Lpp0288), the zinc/iron transporter (Lpp2018) or the heme-binding protein Hbp (Lpp2164) and iron using proteins, like the catalase/peroxidase KatG (Lpp0252), the Fe-S cluster assembly complex (Lpp0651-Lpp0658), the ferredoxin-like 4Fe-4S binding protein (Lpp1898) or the L-serine dehydratase (Lpp0854) (**S3 Table**). Thus, in *L*. *pneumophila* CsrA has a crucial impact on iron homeostasis highlighting yet another global function of CsrA.

## Discussion

The RNA-binding protein CsrA is the key regulator governing adaption of *L*. *pneumophila* to its hosts and therewith the transition between replicative bacteria to transmission competent bacteria. CsrA was first identified in *Escherichia coli* but has now been recognized in several bacterial species among those in many pathogens [12, 13, 27]. CsrA is known to post-transcriptionally control metabolism, motility and virulence by binding to mRNAs of its targets [12, 13]. Here we report that 479 direct CsrA targets exist in the *L*. *pneumophila* genome (**S3 Table**), which is in a similar range as the 467 targets reported for *Salmonella* Typhimurium [64] or the 154 targets reported for *Campylobacter jejuni* [38] when compared to the different genome sizes (3.4Mb for *L*. *pneumophila*, 4.Mb for *S*. Thyphimurium and 1.6Mb for *C*. *jejuni*). Furthermore, we describe a new way by which CsrA may regulate gene expression differently within one operon and demonstrate that CsrA governs the transition from replicating to virulent bacteria in multilayered and complex circuitries with several unique features of *L*. *pneumophila*.

We found that CsrA governs the expression of the virulent phenotype in a dual way. First by directly binding to the mRNAs of major virulence regulators like LqsR, PrmA, LetE and RelA and secondly by interfering with the expression of at least 41 Dot/Icm secreted proteins to assure their timely activity (**S3 Table**). The direct regulation of 26 Dot/Icm effectors by CsrA was previously predicted by searching for the CsrA binding motif A(N)GGA and by showing that their expression was under the control of the LetA/Rsm/CsrA regulatory cascade [25]. Indeed, for 14 of these 26 substrates binding to CsrA was confirmed by our RIPseq analyses. Essential for virulence and successful infection is also the access to iron (for review see [60]). In contrast, elevated iron concentrations can be toxic for the pathogen, thus iron acquisition, usage and storage have to be well coordinated and fine-tuned. Here we show that the maintenance of the iron homeostasis is tightly connected to CsrA as *fur* gene expression, encoding the main regulator of iron homeostasis, is under the direct influence of CsrA. It binds inside the coding sequence of *fur* potentially mediating therewith the stabilization of the mRNA (**S12 Fig)**. The increased transcript stability might be related to a reduced endonucleolytic accessibility in which the cleavage sites are occluded by CsrA. Indeed, the presence of a potential RNase E binding site (A/G)AUU(A/U) directly adjacent to the CsrA-binding motif may suggest RNase E degradation dependent on CsrA. In line with this model, a reduced growth rate at low iron levels and a lower siderophore secretion was detected in a *csrA*^*-*^ strain **(S13B and S13C Fig**). Moreover, the bacterial iron storage protein bacterioferritin (Lpp2460) is transcribed in an RpoS- and LqsR-dependent manner [23, 65] and both regulators are targets of CsrA. Thus CsrA participates in the control of iron homeostasis at the level of iron acquisition, usage and storage.

It was previously shown for *E*. *coli*, that autoregulatory loops regulate CsrA expression and activity [66]. Here we uncovered that CsrA of *L*. *pneumophila* also binds its own mRNA and in addition it directly interacts with the 5' leader/RBS region of its own transcriptional activator, the response regulator PmrA. These findings indicate the presence of autoregulatory circuits to control expression and activity of the Csr system in *L*. *pneumophila*. However the regulatory circuitry differs partly from *E*. *coli*, as we observed inhibition of translation of CsrA by self-binding similar to what is reported for *E*. *coli*, and besides a negative autoregulatory effect of CsrA on its own transcript level what is different to *E*. *coli*. Indeed the *L*. *pneumophila csrA* transcript is downregulated during transmissive/virulent phase [9]. Thus in *L*. *pneumophila* extensive autoregulatory circuitries regulate CsrA expression and translation, but in contrast to *E*. *coli* the transcript levels seem also to be regulated by the TCS PmrBA, as described previously [24] and not only by CsrA itself **(Fig 5)**. Most interestingly, we also observed interaction of CsrA with RelA mRNA indicating that a regulatory mechanism exists whereby CsrA directly affects the synthesis of the alarmone (p)ppGpp and hence the stringent response in *L*. *pneumophila* **(Fig 5).** Surprisingly, our proteomic data suggest that RelA expression is positively regulated by CsrA as a *csrA*^*-*^ strain exhibits lower amounts of RelA protein than the wt strain during exponential growth (**S2 Table**). Thus, a negative feedback regulation of the (p)ppGpp production in *L*. *pneumophila* seems to be present where the inactivation of CsrA by its antagonistic sRNAs weakens the stringent response. This regulatory circuitry is also different to the *E*. *coli* system where CsrA deficiency led to an increase in *relA* expression and elevated levels of (p)ppGpp [67]. The regulatory complexity is further increased as the transcription of *relA* is highly dependent on RpoS and DksA ([15]). Furthermore, CsrA directly interacts with the 5' leader/RBS region of its transcriptional activator, the response regulator PmrA. Thus, the stringent response in *L*. *pneumophila* is controlled by several positive and negative feedback loops including the simultaneous inhibition of RelA on transcript level *via* diminution of RpoS but post-transcriptional activation by CsrA (**Fig 5**). This multilayered regulation and regulatory redundancy might be necessary to increase its robustness and for fine-tuning of the Csr system.

The prominent role of CsrA is further underlined as we provide evidence that CsrA is the link between the stringent response and quorum sensing (QS) in *L*. *pneumophila*. It was shown for *Vibrio* that CsrA could affect the QS circuit by modulating the transcription of the response regulator LuxR or modulating the activity of LuxO indirectly via the sRNAs CsrB, C and D [68, 69]. In *L*. *pneumophila*, we show direct interaction of CsrA to the 5'UTR/RBS region of LqsR suggesting a negative regulation of translation (**Fig 4**). The link of the stringent response with quorum sensing through CsrA allows *L*. *pneumophila* a balanced regulation of the biphasic life cycle. Only the integration of both pathways together ensure the expression of virulence factors, stress adaptations or cell motility with optimal precision to the metabolic state of the cell. Thus, crucial environmental signals like amino acid starvation likely lead to the coordinated expression of stationary phase traits when in parallel the cell density has reached a critical level. This might also be a signal for the necessity for the bacterium to become motile to be able to escape the host cell and find a new host. Indeed, the dependence of motility on the LetA/Rsm/CsrA cascade was shown earlier and an impact of CsrA on the flagellar biosynthesis sigma factor FliA was predicted [10]. Furthermore the QS response *via* the Lqs system is known to mediate motility in *L*. *pneumophila* [21]. We show here, that flagella biosynthesis is regulated through direct binding of CsrA to FleQ and FleR that are controlling the transcription of class II and class III flagella genes, respectively (**Fig 3**). Proteomic data suggest that both are negatively regulated via CsrA-binding to the RBS/start condon region of the transcript. Thus, sequestering CsrA by RsmXYZ from the target-RNAs FleQ, FleR and LqsR results in concerted activation of the flagella biosynthesis and links QS and motility via CsrA. Taken together, CsrA is the central regulator that integrates and coordinates the varying extra- and intracellular stimuli and merges them into a global cell response. The interconnection of flagella biosynthesis, stringent response and QS allows simultaneous preparation of the entire bacterial community to complete the replicative life cycle and to enter into the virulent stage in a cooperative manner.

Similarly to what was described in other bacteria, CsrA of *L*. *pneumophila* is a major regulator of metabolism as numerous enzymes of various metabolic pathways are under the direct control of CsrA (**Table 1**and **S1–S3 Tables**). Indeed, CsrA may also act prominently at the interface of the bipartite metabolism observed in *L*. *pneumophila* [70] balancing the two distinct parts containing the Entner-Douderoff (ED) pathway/ Gluconeogenesis/Pentose Phosphate pathway (PPP) on one hand and the TCA cycle/Amino acid metabolism/Fatty acid metabolism on the other hand. By studying the regulation of the carbon metabolism by CsrA we discovered a new way by which CsrA may modulate transcription and regulates carbon flux. Indeed the particular location of a CsrA binding site within the coding sequence of the glyceraldehyde-3-phosphate dehydrogenase (*gap*) gene of the *tkt/gap*-operon was intriguing (**Fig 6**). Our qPCR analyses showed strong CsrA dependency for the transcription of *gap*, but not for the *tkt* transcript although they are organized in an operon. This effect was not related to changing RNA stability between the transcripts, but *in vitro* transcription assays in presence of Rho, NusG and CsrA disclosed that the premature transcriptional stop caused by Rho was completely absent when CsrA was present. These results led us to propose a model in which CsrA-binding stabilizes the alternative secondary conformation that cover the transcription termination sites. Hence, under optimal nutrient availability the genes of the operon are transcribed together as a unit. In contrast, under certain metabolic conditions, like stress or starvation, it can be advantageous to uncouple the expression of the PPP (transketolase) and the glycolysis to conserve energy and to prevent the synthesis of unused transcripts. Thus, transcription of the inhibitory sRNA and subsequent CsrA-sequestration unmasks the premature terminator promoting the transcriptional block of the downstream glycolytic genes without affecting the transcription of the *tkt* gene. Excitingly, in *L*. *pneumophila* CsrA is able to mediate transcriptional polarity effects by preventing rho-dependent termination, a regulatory mode that might be present also in other bacteria encoding CsrA.

In addition, CsrA affects the production of secondary metabolites and vitamins that are necessary for an undisturbed metabolic flow. Among those are thiamine pyrophosphate, which is essential for the functioning of central enzymes of the carbohydrate metabolism like the pyruvate dehydrogenase, the 2-oxoglutarate dehydrogenase, but also the transketolase. Interestingly, we detected a THI-box riboswitch structure in the 5'UTR leader sequence of the *thi*-operon, a widely used control element found in all kingdoms of life [55] that was overlapping with a CsrA-binding site. Our experiments showed that CsrA is necessary for the fine-tuning of the *thi*-operon expression most likely by modulating the secondary structure of the TPP riboswitch (**Fig 8**). During periods of high metabolic activity the amount of TPP used in the cell needs to be elevated compared to carbon starvation phases. Consequently, the TPP threshold value that leads to the transcriptional or translational blockage *via* the riboswitch must be adapted to the actual conditions. We postulate that the interaction of CsrA with the *thi*-leader sequence reduces its affinity to TPP. This consequently would lead to an increased expression of the genes necessary for the thiamine biosynthesis even in presence of considerable amounts of TPP.

**Fig 9:**
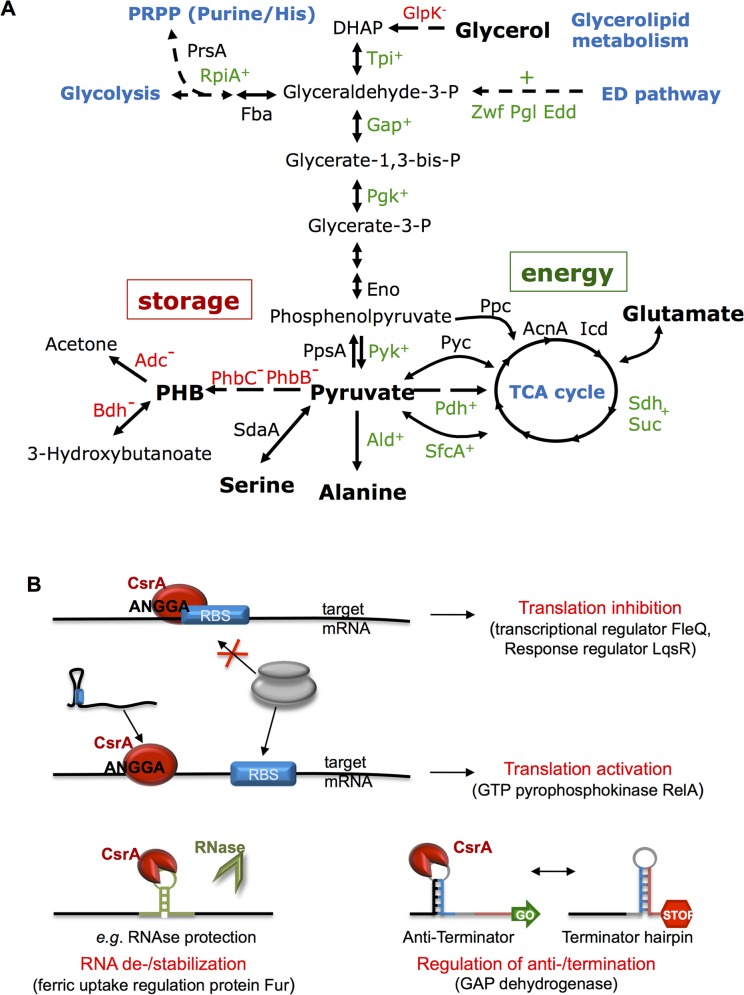
Model how *Legionella* CsrA influences the pyruvate metabolism and which different regulatory functions it exerts. **A)** Carbon flux into the energy production is favored by CsrA, whereas production of the storage molecule PHB is repressed. Additionally, amino acids and glucose, but not glycerol are the preferred carbon source in presence of CsrA. Proteins in red represent a negative effect of CsrA on the pathway, whereas in green, proteins are shown to be under positive control of CsrA. In black, CsrA interacts with the RNA, but no quantitive difference was observed under our condition. PrsA (Lpp0607), Ribose-phosphate pyrophosphokinase, RpiA (Lpp0108), Ribose-5-phosphate isomerase A, GlpD (Lpp1368), Glycerol-3-phosphate dehydrogenase, GlpK (Lpp1369), glycerol kinase, Tpi (Lpp2838), Triosephosphate isomerase, Zwf (Lpp0483), Glucose-6-phosphate 1-dehydrogenase, Pgl (Lpp0484), 6-Phosphogluconolactonase, Edd (Lpp0485), 6-Phosphogluconate dehydratase, Gap (Lpp0153), Glyceraldehyde 3-phosphate dehydrogenase, Pgk (Lpp0152), 3-Phosphoglycerate kinase, Eno (Lpp2020), Enolase, Pyk (Lpp0151), Pyruvate kinase, PpsA (Lpp0567), Phosphoenolpyruvate synthase, Pdh (Lpp1461), Pyruvate dehydrogenase complex, Pyc (Lpp0531), Pyruvate carboxyltransferase, Ppc (Lpp1572) Phosphoenolpyruvate carboxylase, SfcA (Lpp3043), NAD-specific malic enzyme, AcnA (LPP1659), Aconitate hydratase, Icd (Lpp0878), Isocitrate dehydrogenase, Sdh (Lpp0595), Succinate dehydrogenase, Suc (Lpp0597), 2-Oxoglutarate dehydrogenase, Ald (Lpp0986), Alanine dehydrogenase, SdaA (Lpp0854), Serine dehydratase, PhbB (Lpp0621), acetoacetyl-CoA reductase, PhbC (Lpp2038), Polyhydroxyalkanoate synthase, Adc (Lpp0728) Acetoacetate decarboxylase, Bdh (Lpp2264), 3-Hydroxybutyrate dehydrogenase. **B**) CsrA can act as a negative regulator of the translation initiation process by blocking the ribosome binding site of the RNA and hence, interfering with its ribosome interaction. Examples in *L*. *pneumophila* are the transcriptional regulator FleQ and the quorum sensing response regulator LqsR. Binding of CsrA leads to a conformational re-organization of the target-RNA. As a consequence, the RBS is better accessible for the ribosome yielding in a translational activation due to CsrA interaction. This mode of action might be relevant for the *relA* mRNA in *L*. *pneumophila*. CsrA interaction with the RNA can stabilize the target-RNA by blocking RNase-specific binding sites. Contrary, also a destabilization can be triggered by CsrA when its binding leads to conformational changes of the RNA that facilitate the attack of an RNase. In *Legionella*, we suggest that the *fur* mRNA is protected by CsrA against degradation by binding to an A(N)GGA motif overlapping a putative RNase E recognition site. Finally, CsrA can affect transcriptional elongation in a negative (promoting termination) or in a positive way (stabilizing an anti-terminator structure). The transcription of the *gap* gene in *L*. *pneumophila* is only guaranteed in presence of CsrA as binding of the protein prevents the Rho-dependent termination downstream of the *tkt* gene part of the PPP/Glycolysis operon.

Collectively, our data show that CsrA is linking the fine-tuned regulation of the stringent response, quorum sensing, metabolism and virulence and revealed that the decision of the cell between energy production via the TCA cycle and the synthesis of the carbon and energy storage molecule poly-3-hydroxybutyrate (PHB), is coordinated through the activity of CsrA (**Fig 9A).** Furthermore we discovered the first riboswitch in *Legionella* and found that *L*. *pneumophila* CsrA has evolved a mechanism by which it is able to regulate genes organized in the same operon differently, according to the needs of the cell (**Fig 9B**) adding thereby another example for the astonishing diversity of CsrA functions in bacterial cells.

## Materials and methods

### Strains, media, growth conditions and *A*. *castellanii* infection assay

*L*. *pneumophila* strain Paris was cultured in *N*-(2-acetamido)-2-aminoethanesulfonic acid (ACES)-buffered yeast extract broth or on ACES-buffered charcoal-yeast (BCYE) extract agar at 37°C. *A*. *castellanii* ATCC50739 was cultured in PYG 712 medium (2% proteose peptone, 0.1% yeast extract, 0.1 M glucose, 4 mM MgSO_4_, 0.4 M CaCl_2_, 0.1% sodium citrate dihydrate, 0.05 mM Fe(NH_4_)_2_(SO_4_)_2_ x 6H_2_O, 2.5 mM NaH_2_PO_3_, 2.5 mM K_2_HPO_3_) at 20°C. *A*. *castellanii* infection assays were conducted as previously described [9][46]. Intracellular multiplication was monitored using a 300μl sample, which was centrifuged (14500 rpm) and vortexed to break up amoeba. The number of colony forming units (CFU) was determined by plating on BCYE agar. Each infection was carried out in duplicates or triplicates.

### *csrA* mutant construction and plasmids

Mutant strains of *L*. *pneumophila* were constructed as described previously [71]. In brief, the gene of interest was inactivated by introduction of an apramycine resistance (apra^R^) cassette into the chromosomal gene by 3-steps PCR using the following primers: CsrA_F TTGCAATATAAGCTCAAGATAC and CsrA_Inv_R gctgatggagctgcacat gaaTAAATTTCTTCACGATGAACAG, CsrA_Inv_F gagcggatcggggattgtcttAAAGAAT CTGATGATTCGGAAC and CsrA_R ATTGTTGATAACAAAAGTATCC. To amplify the apramycine cassette the primers Apra_F TTCATGTGCAGCTCCATCAGC and Apra_R AAGACAATCCCCGATCCGCTC were used. The final product was cloned into the pGEM-T easy vector (Promega). For the beta-lactamase reporter assay, the predicted TPP riboswitch region was amplified (TPP_F GAATTCGGCGCGGGGTGTCGGGAAATC, TPP_R GAATTCAAAAGGGAACCATGCCTTAAAAAGG) and cloned into the pXDC61 upstream of the *blaM* gene using the EcorI restriction side.

### Pigmentation assay, stress responses, PHB quantification, iron deficiency, CAS assay and TPP riboswitch beta-lactamase assay

#### Pigmentation

For quantifying pigment accumulation, 1 ml samples—obtained from a 5 day old broth culture grown at 37°C—were centrifuged at 16,000 × g for 10 min and supernatants measured at OD_550nm_.

#### Western blot

Total soluble protein was extracted by sonication and subsequent centrifugation. The protein amount of the supernatant was quantified by Bradford and 1μg of total protein was separated by SDS-PAGE, blotted on PVDF membranes and analyzed using anti-FlaA, CsrA and Flag antibodies as described before.

#### Stress assays

Sodium sensitivity was tested by plating serial dilutions of exponentially or post-exponentially grown bacteria on BYE agar containing or lacking 100 mM NaCl. CFUs were counted and percentage of sodium sensitive bacteria was calculated. For oxidative stress tests, *L*. *pneumophila* wt and *csrA*^*-*^ mutants were grown in BYE medium until exponential phase (OD2.5). Subsequently, the cells were pelleted and washed twice in PBS and resuspended in PBS at an optical density of OD 1.0. A final concentration of 5mM paraquat (Sigma-Aldrich) was added and the cultures were incubated for 2h at 37°C. At time point 0h, 0.5h, 1h and 2h samples were taken and serial dilutions were plated on BYCE plates to estimate bacterial survival. To analyze resistance to moderate acidic stress of wt and mutant strains, cultures were grown until OD 2.5, washed and diluted to OD 0.1 in BYE medium at pH ranging from 6.3 to 7.2. The bacteria were grown for 20h at 37°C under shaking and the OD was measured.

### PHB quantification

BODIPY 493/503 (Molecular Probes) was solubilized in DMSO at a concentration of 100μg/ml. Bacterial cultures of wt and *csrA*^*-*^ mutant were grown in BYE and 500μl were centrifuged for 3 min at 5000g at different ODs. Pellets were resuspended in 35% ethanol, adjusted to OD 0.1 and incubated for 20 min at room temperature (RT). After centrifugation, the pellet was resuspended in 990μl PBS and 10μl BODIPY stock solution and incubated for 5min, RT. The cells were pelleted and washed once with 1ml PBS before resuspended in 100μl PBS. Fluorescence was analyzed with a MACSQuant flow cytometer (Miltenyi Biotec).

### Iron deficiency and CAS assay

For the iron deficiency assays, *L*. *pneumophila* wt and *csrA* mutant strains were grown in Minimal Medium at an initial OD600 of 0.1 containing 0 (+/- DFX), 25, 50, 100, 250, 500 and 1000μM of additional iron-pyrophosphate. After 24h of growth at 37°C, 170rpm, absorption of the cultures were measured at OD600. To quantify the siderophore secretion wt and *csrA*^*-*^ mutants were grown in BCYE medium until E phase. The cells were pelleted and washed twice with Minimal medium without additional iron and resuspended at an OD of 0.2. At time points 0, 2h, 4h, 6h, 8h and 24h, cells were centrifuged and 150μl of the supernatant were mixed with 30μl of CAS solution (60.5mg Chromeazurol S in 50m H_2_O, 2.7mg FeCl_3_*6H_2_0 in 10mM HCl, 73mg HDTMA in 40ml H_2_O were mixed and autoclaved) and the OD655 was measured after 30 min of incubation at RT.

### Beta-lactamase assay

Strains containing the TPP-pXDC61 plasmid were grown in Minimal Medium containing 0, 1, 2mM thiamine pyrophosphate at 37°C, 200rpm without IPTG until reaching early exponential phase. Cells were harvested, resuspended in PBS and sonicated. Protein concentration was quantified by Bradford. 10μg of total protein in 100μl PBS were mixed with 50μl Nitrocefin (0.5mg/ml in PBS + 5% DMSO) and the enzyme kinetic was followed with a spectrophotometer at 486nm. BlaM activity was calculated from the initial slope.

### CsrA co-immunoprecipitation and RIPseq

For CsrA expression in *L*. *pneumophila* Paris (containing a double Flag-Tag at the C-terminal end or without Flag-Tag) full-length cDNAs encoding CsrA (*lpp0845*) were amplified by PCR using primer CsrA-F tctagaATGTTGATTTTGACTCGGCGTATAG and CsrA-R ctgcagTTATACTGCTTGTTCCGAATCATC or CsrA-Flag-R ctgcagTTACTTATCGTCA TCGTCCTTGTAGTCCTTATCGTCATCGTCCTTGTAGTCTACTGCTTGTTCCGAATCATC, respectively, and cloned into pGEM-T easy vector (Promega). The fragment was verified by sequencing, cut with XbaI/PstI and ligated into the pBC KS vector under the control of the Mip (*lpp0855*) promoter region of *L*. *pneumophila*. Competent *L*. *pneumophila* bacteria were transformed by electroporation and positive colonies were selected on 10μg/ml chloramphenicol and sequenced. *L*. *pneumophila* expressing CsrA+2xFlagTag and *L*. *pneumophila* expressing CsrA without Flag-Tag as negative control were grown in broth until exponential growth (OD 2). Cells were cross linked with formaldehyde (final concentration 1,1%) over night at 4°C on a rotating platform, then formaldehyde was quenched by adding 125 mM glycine and pellets were rinsed twice with PBS. Pellets were resuspended in lysis buffer (50mM HEPES-KOH pH7.5, 150mM NaCl, 1mM EDTA, 1% Triton X-100, 0.1% Na-deoxycholate, protease inhibitor), sonicated and total protein concentrations were adjusted to 1mg/ml. The total protein of both samples, CsrA+2xFlagTag and negative control, was cleared separately by BSA-blocked Dynabeads protein G (Invitrogen) and subsequently incubated with Dynabeads protein G coupled to Anti-Flag antibodies (Sigma) over night at 4°C on a rotating platform. Samples and negative control were washed twice with Lysis buffer containing 350mM NaCl and 5 times with wash buffer (10mM Tris-HCl pH 7.5, 250 mM NaCl, 0.5% NP-40, 0.5% Na-deoxycholate, 1mM EDTA). Subsequently beads were washed with TE buffer, resuspended in elution buffer (50mM Tris-HCl pH 8.0, 1mM EDTA, 1% SDS) and incubated at 65°C for 30 min. Cross-linking was reversed and DNA and protein were digested. The RNA was metal-catalyzed heat fragmented to a size around 100-200nt using the RNA fragmentation kit. The RIPseq library IP1 and IP2 were constructed as described previously [72, 73]. For IP7, 8, and 9 the RNA of the independent samples was purified and further processed according to the TruSeq stranded mRNA sample preparation guide of Illumina. The two parallel processed samples were ligated with adaptor 6 (positive CsrA+2xFlagTag library) and adaptor 12 (negative control, minus FlagTag), respectively, the quantity was determined using a Qubit 2.0 (Invitrogen) and the quality was checked using a Bioanalyzer. High quality libraries were sequenced using an Illumina HiSeq platform. This experiment was done in 5 replicates.

### Analysis of deep sequencing data

The reads in FASTQ format generated by Illumina sequencing were filtered using FastXclipper from the FastX toolkit (http://hannonlab.cshl.edu/fastx_toolkit/) and Tagdust [67] for adapter removal. Trimming of reads was performed using Sickle (https://github.com/najoshi/sickle). To assure high sequence quality, we used a cutoff phred score of 20. After trimming, all the sequence reads shorter than 18nt were eliminated. Reads were aligned to the *L*. *pneumophila* strain Paris chromosome sequence (NCBI Acc.-No: NC_006368.1) using Bowtie 2 software [68]. Only the uniquely mapped reads are kept, so, a read cannot contribute for the coverage value at different positions. Duplicate reads were removed from mapping results (with samtools rmdup) and BAM files were built using the Samtools software [69].

### RIPseq data and enrichment of CsrA target analyses

For sample and control, coverage files (wiggle files) were generated for the forward and the reverse strand and normalized according to the number of mapped base pairs. For each couple sample/control, a scale factor was applied to scale the small sample up to the bigger sample. To define CsrA-bound RNA regions, enrichment regions (peaks) were detected using a python script “sliding_window_peak_calling_script.py” previously described [38] and available at Zenodo (https://zenodo.org/record/49292). Peak detection was performed separately for the forward and reverse strand of each replicon, according to the author description and with a minimum required fold change (FC) of 5 for the enrichment. To identify CsrA targets, overlaps between gene annotations and enrichment regions were performed with bedtools suite [74]. Depending on the library preparation, we used peaks with sequence reads in the sense in which the genes are described for IP-1 and IP-2 and peaks with reads in the reverse sense as the genes are transcribed for IP-7, IP-8 and IP-9. Overlaps with less than 10 nts were discarded. BAM files were imported into Artemis [75] to manually validate the identified CsrA targets.

### Data availability

The sequence reads after adapter removal as well as coverage files of the RIP-seq libraries have been deposited in the NCBI Gene Expression Omnibus (GEO) [76] under the accession number GSE94068.

### CsrA overexpression and purification

Full-length cDNAs encoding CsrA (*lpp0845*) were amplified by PCR using primer CsrA-F ccatggTGATTTTGACTCGGCGTATAG and CsrA-R aagcttTACTGCTTGTTCCGAATCA TCAG. Fragments were cloned in frame into the expression vector pET-28a (Novagen), which adds a hexa-histidine tag to the C-terminus of the protein; positive clones in *E*. *coli* DH5α were selected on 50μg/ml kanamycine and sequences were verified. *E*. *coli* BL21 (Invitrogen) cells were used for protein overexpression. The cells were grown in LB medium, containing, 50 μg ml^-1^ kanamycine, at 20°C. Protein production was induced by adding 0.5 mM IPTG at A_600_ ~0.5, and cells were harvested in late exponential phase by centrifugation at 4°C. Cells from 1 liter culture were resuspended in 1 ml buffer A– 100 mM Tris/HCl (pH 7.5), 150 mM NaCl, 100mM KCl, 5 mM MgCl_2_, 2 mM DTT, 5% (v/v) glycerol and a cocktail of protease inhibitors (Sigma) at the concentration recommended by the manufacturer. Cells were disrupted by sonication, centrifuged (18,000 × *g*, 30 min, 4°C) and the supernatant was applied to a Ni-NTA affinity column (GE Healthcare) equilibrated with buffer A including 10 mM imidazole. The column was washed with the same buffer containing 100 mM imidazole then the enzyme was eluted with 500 mM imidazole in buffer A. Fractions were dialyzed against buffer A and concentrated by centrifugation (Microcon, 3 kDa cut-off, Millipore) to a final concentration of 0.5 mg protein/ml. Proteins were quantified according to Bradford using BSA as standard. Aliquots of 20μl were frozen in liquid N_2_ and kept at -80°C until further use. The quality of the purification was determined after SDS-PAGE analysis (4% stacking gel and 12% running gel) and staining with Brilliant Blue G—Colloidal Concentrate (Sigma).

### *In vitro* RNA transcription and Electrophoretic Mobility Shift Assay (EMSA)

The region corresponding to target RNAs selected according to the RIPseq experiments was amplified from bacterial DNA adding a T7 promoter at the 5' end. PCR products were used in MEGAshortscript T7 Kit (Ambion) to produce *in vitro* RNA (**S6 Table**). 2μM of Biotin-11-CTP was added into the reaction mix for later detection. This reaction mix was incubated at 37°C for 2h and the RNA was purified by Phenol/Chloroform extraction. The RNA concentration was estimated by UV absorption at 260nm. For 10μl interaction assays, 200nM of RNA was combined with varying concentrations of purified CsrA-His (0–5μM) and incubated in buffer A in presence of 250ng tRNA yeast (Invitrogen) for 30min at RT. Subsequently, samples were fractionated under non-denaturing conditions on Blue-Native PAGE and blotted to BrightStar-Plus transfer membranes (Ambion). Membranes were blocked in PBS buffer containing 0.1% Tween-20 and 1% ECL blocking agent (GE Healthcare) for 1h at RT and, afterwards, incubated for 1h in the same buffer including mouse anti-biotin antibodies (Invitrogen). After washing and binding of secondary antibodies (anti-mouse Ig-HRP, Dako), the RNA-bands were visualized with ECL Plus Western blotting solutions (GE Healthcare) and detected with a G-box (Syngene).

### *In vitro* RNA transcription termination assays

CsrA was over-expressed as described above. Full-length cDNA of the (transcription termination factor Rho (*lpp3002*) and NusG (*lpp0382*) were amplified by PCR using Rho-F ggatccATGAATCTTAGTGAACTTAAGCAATTAC/Rho-R ctcgagTTATTCCTGACGCTT CATTGCATC and NusG-F ccATGGTCGAGGAAAACAAAGCAAAACAG/NusG-R ctcgagTGTTTTTTCTACTTGACTGAACTC, respectively. Fragments were cloned in frame into the expression vector pET-28a (Novagen), which adds a hexa-histidine tag to the N-terminus of the protein. The Rho and NusG proteins were over-expressed and purified as described for CsrA, except buffer R was used instead– 50 mM Tris/HCl (pH 7.5), 50mM KCl, 2 mM DTT, 10% (v/v) glycerol and a cocktail of protease inhibitors (Sigma) at the concentration recommended by the manufacturer. Template DNA for the T7 RNA polymerase was amplified from wt *L*. *pneumophila* Paris using primer pairs GapTer-F TGTAATACGACTCACTATAGGATCTGGCATCGATGTGACCG and GapTer- R TATGACCCATTGCCGCATCTC. Transcription termination assays were performed as follows: 20μl reaction mixtures containing 70nM template DNA, 20U T7 RNA polymerase + transcription buffer (Thermo Scientific), 1μM NusG, 0–2μM Rho, 0–5μM CsrA, 0.5μl RNaseOut (Life Technologies), 100μM ATP, GTP, UTP; 25μM CTP + 25μM CTP-11-Biotin (Roche) were incubated for 2h at 37°C. The reaction was stopped with 115μl 5mM EDTA before extraction in phenol:chloroforme:isoamyl alcohol and precipitation with ethanol and Na-acetate. Pellets were resolved in 1x RNA loading dye (Thermo Scientific), incubated for 10 min at 65°C and analyzed by 10% urea-polyacrylamide electrophoresis. Samples were blotted to BrightStar-Plus transfer membranes (Ambion) and visualized as described for EMSA assays.

### Northern blot analysis

Ten micrograms of total RNA isolated from wt, *csrA*^-^ and the complemented *csrA*^-^ strain were size-separated on a 1.5% denaturing agarose gel and transferred onto a positively charged nylon membrane by capillarity. The gel was photographed under ultraviolet light to capture ethidium bromide staining of ribosomal RNA bands for loading controls. RNA was cross- linked to membranes by exposure to UV light for two minutes and membranes were prehybridized in Ultrahyb buffer (Ambion, AM8670) for 1h. RNA probes radioactively labelled with α-P^33^-UTP (Perkin-Elmer, BLU007X500UC) were generated using the T7 Maxiscript kit (Ambion, AM1314) and PCR templates were amplified from genomic DNA using primers T7-gapNB_F, gapNB_R, T7-tktNB_F and tktNB_R listed in **S7 Table**. The membrane was hybridized at 68°C by adding the radiolabeled probes overnight. Blots were washed twice at the hybridization temperature in 2X SSC, 0.1% SDS followed by two washes in in 0.1X SSC, 0.1% SDS. Membranes were wrapped in saran wrap and subsequently used to expose to films.

### RNA isolation, labeling and microarray analyses

Total RNA was extracted as previously described [77]. Paris wt and mutant strains were grown in AYE medium, and harvested for RNA isolation at the E (OD 2.5) and PE growth phase (OD 4.3). RNA was prepared in triplicates (three independent cultures) and each RNA sample was hybridized twice to the microarrays (dye swap). RNA was reverse-transcribed with Superscript indirect cDNA kit (Invitrogen) and labeled with Cy5 or Cy3 (Amersham Biosciences) according to the supplier’s instructions. The design of microarrays containing gene-specific 70mer oligonucleotides based on all predicted genes of the genome of *L*. *pneumophila* strain Paris (CR628336) and its plasmid (CR628338) was previously described [9]. Hybridization was performed following the manufacturers’ recommendations (Corning) using 250 pmol of Cy3 and Cy5 labeled cDNA. Slides were scanned on a GenePix 4000A scanner (Axon Instruments). Laser power and/or PMT were adjusted to balance the two channels and the resulting files were analyzed using Genepix Pro 4.0 software. Spots were excluded from analysis in case of high local background fluorescence, slide abnormalities, or weak intensity.

Data normalization and differential analysis were conducted using the R software (http://www.R-project.org). No background subtraction was performed, but a careful graphical examination of all the slides was conducted to ensure a homogeneous, low-level background in both channels. A loess normalization [78] was performed on a slide-by-slide basis (BioConductor package marray; http://www.bioconductor.org/packages/bioc/stable/src/contrib/html/marray.html). Differential analysis was carried out separately for each comparison between two time points, using the VM method (VarMixt package [79]), together with the Benjamini and Yekutieli [80] p-value adjustment method. If not stated otherwise, only differently expressed genes with 1.5-fold-changes were taken into consideration. Empty and flagged spots were excluded from the data set, and only genes with no missing values for the comparison of interest were analyzed.

### LC-MS/MS and data analysis

*Legionella* was grown in triplicates to E phase (OD2.5) and cells were lysed in 20 mM HEPES pH 8.0, 8 M urea, 1 mM sodium orthovanadate, 2.5 mM sodium pyrophosphate and 1 mM glycerophosphate by sonication. The protein concentration in the supernatants of each replicate was measured using a Bradford assay (Biorad) and equal protein amounts, each containing 1 mg total protein, were used for further analysis. Proteins in each sample were reduced with 5 mM DTT and incubation for 30 minutes at 55°C and then alkylated by addition of 100 mM iodoacetamide and incubation for 15 minutes at room temperature in the dark. Both samples were further diluted with 20 mM HEPES pH 8.0 to a final urea concentration of 2 M and proteins were digested with 10 μg trypsin (Promega) (1/100, w/w) overnight at 37°C. Peptides were then purified on a Sep-Pak C18 cartridge (Waters) and 50 μg peptides of each sample was re-dissolved in 50 μl solvent A (0.1% formic acid in water/acetonitrile (98:2, v/v)) of which 2 μl was injected for LC-MS/MS analysis on an EASY-nLC 1000 system (Proxeon, Thermo Fisher Scientific) in line connected to a Q Exactive HF mass spectrometer with a Nanospray Flex Ion source (Thermo Fisher Scientific). Peptides were loaded in solvent A (0.1% formic acid in water) on a reverse-phase column (made in-house, 75 μm I.D. x 250 mm, 1.9 μm beads C18 Reprosil-Pur, Dr. Maisch) and eluted by an increase in solvent B (0.1% formic acid in acetonitrile) in linear gradients from 5% to 23% in 100 minutes, then from 23% to 40% in 40 minutes and finally from 40% to 55% in 20 minutes, all at a constant flow rate of 250 nl/min. The mass spectrometer was operated in data-dependent mode, automatically switching between MS and MS/MS acquisition for the 15 most abundant ion peaks per MS spectrum. Full-scan MS spectra (300–1700 m/z) were acquired at a resolution of 60,000 after accumulation to a target value of 3,000,000 with a maximum fill time of 20 ms. The 15 most intense ions above a threshold value of 400,000 were isolated (window of 1.4 Th) for fragmentation by HCD at a normalized collision energy of 28% after filling the trap at a target value of 100,000 for maximum 25 ms with an underfill ratio of 10%. The S-lens RF level was set at 60 and we excluded precursor ions with single, unassigned and charge states above six from fragmentation selection.

Data analysis was performed with MaxQuant (version 1.5.3.8) [81] using the Andromeda search engine [82] with default search settings including a false discovery rate set at 1% on both the peptide and protein level. Spectra were searched against two databases with *L*. *pneumophila* Paris proteins encoded by the chromosome (database containing 3142 protein sequences) and the plasmid (database containing 142 protein sequences) (http://genolist.pasteur.fr/LegioList/) with a mass tolerance for precursor and fragment ions of 4.5 and 20 ppm, respectively, during the main search. Enzyme specificity was set as C-terminal to arginine and lysine, also allowing cleavage at proline bonds and a maximum of two missed cleavages. Oxidation of methionine residues was set as variable modification and carbamidomethyl formation of cysteine residues was set as a fixed modification. Only proteins with at least one unique or razor peptide were retained leading to the identification of 1662 *L*. *pneumophila* proteins (**S8 Table**). Proteins were quantified by the MaxLFQ algorithm integrated in the MaxQuant software [83]. A minimum ratio count of two unique or razor peptides was required for quantification. Further data analysis was performed with the Perseus software (version 1.5.3.0) after loading the protein group file from MaxQuant. Proteins only identified by site, reverse database hits and contaminants were removed and replicate samples were grouped. Proteins with less than three valid values in at least one group were removed and missing values were imputed from a normal distribution around the detection limit. After log_2_ transformation of the LFQ intensity values, a t-test was performed (FDR = 0.05 and S0 = 1) to compare both strains and reveal significantly up- and downregulated proteins (**S2 Table**). After Z-scoring, the intensity values of each protein were also visualized on a heat map after non-supervised hierarchical clustering (**Fig 2**). The mass spectrometry proteomics data have been deposited to the ProteomeXchange Consortium via the PRIDE partner repository [84] with the dataset identifier PXD004730.

## Supporting information

S1 Fig **CsrA knock down mutant in *Legionella pneumophila* Paris and phenotypic analysis of a transmissive phenotype features A**) Schematic representation of the *lpp0845* gene encoding CsrA. An apramycin antibiotic resistance cassette was introduced after the aminoacid Tyr 48 leading to a truncated CsrA protein. **B**) Confirmation of a significant reduced expression of the CsrA protein after disruption of its gene: 50μg of total protein of wt and *csrA*^-^ grown until E (exponential) and PE (post-exponential) phase were separated on a 16% Tris/Tricine PAGE and western-blot analysis was performed with anti-CsrA antibodies. Lower panel, total protein as loading control; Numbers indicate the intensity x 1000. **C**) Western-blot detection of 1 μg of total protein against FlaA: Induction of the most abundant flagellar protein, FlaA is expressed in the csrA^-^-mutant already during exponential growth but not in wild-type *Legionella*. Lower panel, total protein as loading control; Numbers indicate the intensity x 1000. **D)** The production of the secreted *Legionella*-pigment Pyomelanin is elevated in the *csrA*^-^ mutant and is reversed by complementation with full-length *csrA* (csrApCsrA). **E**) The *csrA*^-^ mutant in E phase (csrA-E) is sensitive to 100mM NaCl while the wt strain is highly resistant during exponential growth. When reaching PE phase wt and mutant show similar sensitivity to salt stress. **F**) The E-phase *csrA*^-^ strain has higher survival rates over time under oxidative stress induced by 5mM paraquat than the wt strain. **G**) During E phase, *csrA*^-^ (csrA-E) is more resistant to mild acidification (pH4.8) than the wt (wt-E) whereas in PE phase, no difference was observed. Each value represents the mean +/- SD of at least three independent experiments.
(TIF)

S2 Fig Analysis of the expression of the FLAG-tagged CsrA compared to wt expression. **A)** Separation of the crude protein extract of wt and the wt expressing the FLAG-tagged CsrA under the promoter of the *Legionella mip* gene on a 16% Tris/Tricine SDS-PAGE gel. Western blot analysis was performed with anti-CsrA antibodies. Numbers in brackets indicate the total band intensities (mean pixel values) as determined by GeneTools (SynGene). **B**) Western blot analysis showing the input of protein used for the RIPseq experiments using anti-Flag antibodies (Sigma).
(TIF)

S3 Fig Beta-lactamase reporter assay to analyse the activity of the mRNA CsrA-binding regions of *fleQ*, *lqsR*, *relA* and *rpoS*. Beta-lactamase (BlaM) activity was measured from wt and *csrA*^-^ *L*. *pneumophila* strains that contained the pXDC61 plasmid carrying the potential CsrA-binding region identified by RIPseq upstream of the BlaM gene (grown in minimal medium). The predicted A(N)GGA motifs were mutated (m) or not (p). **A)** *fleQ*, **B)** *lqsR*, **C)** *relA***, D)** *rpoS*. BlaM activity of 10μg total protein from the wt or the *csrA*^-^ strain was measured. Each value represents the mean +/- SD of three independent experiments. These data confirm the transcript and/or proteome results wherein FleQ is negatively regulated and RelA is positively affected by the presence of CsrA. LqsR and RpoS expression are negatively affected by CsrA according to the BlaM activity.
(TIF)

S4 Fig CsrA interacts with the RNA of the 5' region of the *letE* gene downstream of the RpoD-transcription start site (TSS), but not within the region between the two TSSs. **A**) Schematic representation of the transcriptional unit of the *lpp0602 letE* gene of *L*. *pneumophila* depicts two independent TSS, the first depending on the RpoD, the other on the RpoN sigma factor. The green arrow indicates the identified CsrA-binding site, the red arrow symbolizes the region used as negative control in the EMSA with 200nM of biotinylated *letE* mRNA below: Lane 1: no CsrA, lane 2: 2.0 μM CsrA, lane 3: 5.0 μM CsrA.
(TIF)

S5 Fig No interaction between CsrA and *hfq* mRNA can be observed confirming RIPseq data. In agreement with our RIPseq data, EMSA with 200nM of biotinylated *hfq* RNA confirms that indeed no interaction of purified CsrA with *hfq* mRNA occurs *in vitro* even though two potential A(N)GGA motifs are present upstream and downstream of the translation start site, respectively. 200nM of biotinylated *hfq* mRNA and increasing concentrations of recombinant CsrA in 6% Native Tris-PAGE were used. Lane 1: no CsrA, lane 2: 1.0 μM CsrA, lane 3: 2.0 μM CsrA, lane 4: 5.0 μM CsrA, lane 5: 10 μM CsrA
(TIF)

S6 Fig CsrA interacts with transcripts of various Dot/Icm-effector proteins like the eukaryotic NTPDase (*lpp1033*), LidA (*lpp1002*) and YlfA (*lpp2246*). Electromobility shift assays (EMSA) with 200nM of biotinylated RNA combined with varying concentrations of purified CsrA-His in 6% Native Tris-PAGE. Lane 1: no CsrA, lane 2: 0.5 μM CsrA, lane 3: 1.0 μM CsrA, lane 4: 2.0 μM CsrA, lane 5: 5.0 μM CsrA, lane 6: 5.0 μM CsrA + 2.0 μM unlabled RsmZ.
(TIF)

S7 Fig Northern blot analyses of the *tkt/gap* operon in the wt, *csrA*^*-*^ and the complemented strain shows a higher amount of tkt transcript in the *csrA*^*-*^ background. Northern Blot analysis of bacterial lysates from *L*. *pneumophila* Paris strain wt, *csrA*^-^ and the complemented strains grown at the exponential phase using a *gapdh* probe (upper panel) for the operon transcript and a *tkt* probe (lower panel) for the *tkt* transcript. 23S and 16S RNAs signals are shown as loading controls (right panel).
(TIF)

S8 Fig Double mutation of the CsrA-binding/transcriptional termination site within the *gap* gene leads to premature termination even in absence of Rho and independent of CsrA. **A**) Mfold-prediction of the RNA secondary structure of the CsrA-binding region within the *gap* mRNA showing the preferred conformational status due to the double mutation. **B**) EMSA shift assay of the *in vitro* transcribed double-mutated RNA at different concentrations of CsrA, lane 1: no CsrA, lane 2: 2μM CsrA, lane 3: 5μM CsrA. **C**) *In vitro* transcription termination assay without additional purified Rho protein (left) or with 1μM Rho protein (right) at 0μM CsrA (-), 0.5μM CsrA (+) and at 1μM CsrA (++). Resulting transcripts are separated on a 10% urea-PAGE gel.
(TIF)

S9 Fig Effect of CsrA on the production and usage of the intracellular carbon/ energy storage polyester, poly-3-hydroxybutyrate (PHB). **A**) Schematic representation of the CsrA interaction region with the PhbC (Lpp2038)-transcript located in the 5'UTR/RBS. Above, coverage of the reads gained from a representative RIPseq experiment compared to the negative control**. B**) EMSA with 200nM of biotinylated *phbC* mRNA, lane 1: no CsrA, lane 2: 2.0 μM CsrA, lane 3: 5.0 μM CsrA. **C**) Staining and quantification of the PHB positive cells with Bodipy 493/503 by flow cytometry during exponential (E), post-exponential (PE) and stationary phase comparing wt and CsrA-mutant. Each value represents the mean +/- SD of three independent experiments. In absence of CsrA, the amount of PHB is significantly increased during E and S phase indicating a higher synthesis or reduced usage of PHB in the mutant bacteria than the wt. **D**) Gating strategy for flow cytometry analysis of *Legionella*. Representative scatter plot distribution of PHB fluorescence for Bodipy 493/503 stained wt and a *csrA*^*-*^ and complemented strain during different growth phases based on FSC-A vs SSC-A and SSC-A vs SSC-H subsets to discriminate single bacteria. The fluorescence data was collected using a 530± 30 nm band pass filter; the threshold of PHB positive cells was determined by unstained *Legionella* cells (negative control).
(TIF)

S10 Fig Effect of mutations on beta-lactamase activity of the thiamine-binding site or the CsrA-binding site of the predicted TPP riboswitch element. **A)** Schematic representation of the *thi*-operon in *L*. *pneumophila* indicating the region that was mutated for the BlaM activity assay. **B)** Normalized BlaM activity at no, 1mM and 2mM of extracellular TPP concentration in *L*. *pneumophila* grown in a minimal medium. BlaM activity is significantly reduced at 1mM TPP when the CsrA-binding site was mutated (mCsrA). Similarly, the activity dropped significantly for the non-mutated *thi*-element at 2mM TPP, but the mutation of the thiamine-binding site (mTPP) abolished the dependency on TPP completely.
(TIF)

S11 Fig *thi*-operon mRNA-levels are significantly reduced dependent on extracellular TPP concentrations and in absence of CsrA. **A)** Schematic representation of the *thi*-operon in *L*. *pneumophila* and the region used for qPCR amplification. **B)** The transcript level of the TPP riboswitch region is not affected in presence of 2 mM TPP and CsrA (left) whereas downstream of the predicted terminator region the transcript level is lower in presence of 2 mM TPP and in the *csrA*^-^ background, in presence and absence of TPP compared to the wt (right). Complementation of *csrA*^-^ strain with the *csrA* gene restored the transcript levels to wt level.
(TIF)

S12 Fig Effect of CsrA on *fur* mRNA stability. **A**) Schematic representation of the *fur* gene organisation including the potential CsrA-binding site illustrated by the coverage of the reads obtained from a representative RIPseq experiment. **B**) To analyse the interaction, two independent EMSAs with 200nM of biotinylated RNA of region Fur1 mRNA and Fur2 mRNA together with purified CsrA were performed. Only the position inside the CDS (Fur2), but not the region around the RBS (Fur1) reacted with CsrA *in vitro*.: Lane 1: no CsrA, lane 2: 0.5 μM CsrA, lane 3: 1.0 μM CsrA, lane 4: 2.0 μM CsrA, lane 5: 5.0 μM CsrA. C) RNA stability assay of the Fur-transcript in absence of CsrA (red) and during over-expression of CsrA (green) compared to wt. qRT-PCR was performed from BYE cultures after addition of 100μM rifampicine for 0, 5, 10 and 20 min. Each value represents the mean +/- SD of at least two independent experiments. RNA half life was calculated from the average of the time points compared to the value at t0 according to t1/2 = t*ln(2)/ln(N0/N(t)).
(TIF)

S13 Fig *fur* transcription, growth in different concentrations of extracellular iron and siderophore secretion are dependent on CsrA. **A)** qRT-PCR results of the *fur* transcript at different growth stages (OD) of the wt and *csrA*^*-*^ strain showed lower expression levels of the *fur* gene in E-phase (OD1-3) in absence of CsrA. No differences were noticed during the transition (OD4) and in the PE-phase. Complementation restored the phenotype. **B)** A growth defect of the *csrA*- and the Δ*fur* mutant compared to the wt was observed at very low (0 μM) and high (>500 μM) iron concentration added to the minimal medium. Similarly, the addition of the iron chelator, DFX, abolished the growth of all strains. **C) A** CAS assay was performed to monitor siderophore secretion. Reduction of free iron was measured as described in M&M. Our results showed that in the *csrA*^-^ strain the amount of free iron was stable over time indicating that no iron siderophore complexes were present. In contrast, a clear reduction of free iron was found for the wt and the complemented strain suggesting siderophore secretion in both.
(TIF)

S1 Table Differentially expressed genes according to transcriptome analyses of wt and *csrA*. (DOCX)

S2 Table Proteins identified as differentially expressed by MS analyses (FDR < 0.05). (DOCX)

S3 Table RNA targets identified using Co-immunoprecipitation with anti-CsrA antibodies followed by deep sequencing (RIPseq) analyses. (DOCX)

S4 Table Enrichment of GGA motifs in the peaks of the co-IP as compared to the control IP. (DOCX)

S5 Table Targets identified in all three approaches (RIPseq, transcriptome and proteome data). (DOCX)

S6 Table *In vitro* RNA used in the Electrophoretic Mobility Shift Assays with CsrA (potential CsrA-binding sides are highlighted in yellow, start codon in red). (DOCX)

S7 Table Additional primers used in this study. (DOCX)

S8 Table Complete proteomics data set. (XLSX)
