# Supplementary figures and images for: The Legionella pneumophila genome evolved to accommodate multiple regulatory mechanisms controlled by the CsrA-system
Source: PLoS Genet. 2017 Feb 17;13(2):e1006629. doi: 10.1371/journal.pgen.1006629 (PMC5338858; doi:10.1371/journal.pgen.1006629)

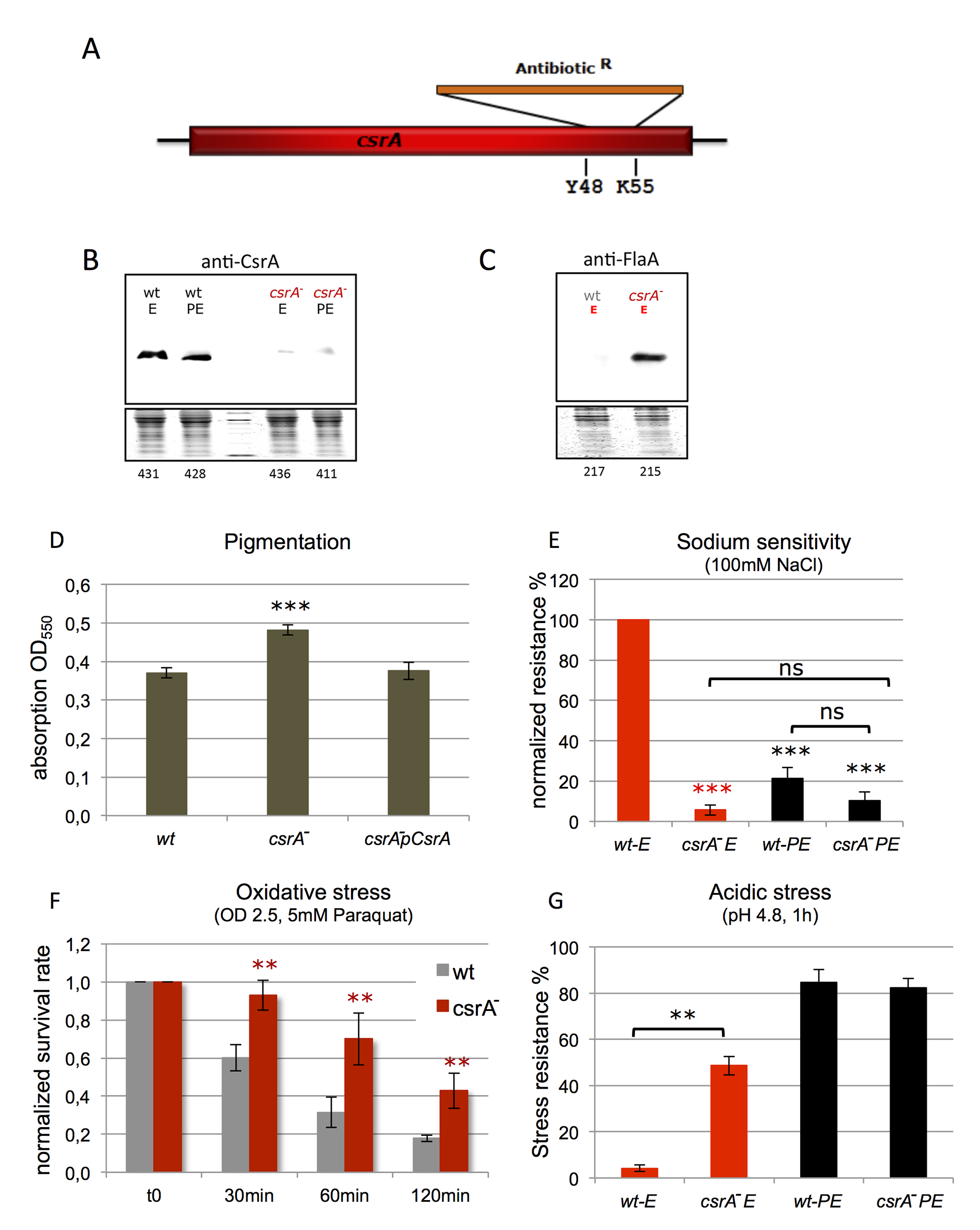

Supplement: S1 Fig — CsrA knock down mutant in Legionella pneumophila Paris and phenotypic analysis of a transmissive phenotype features A) Schematic representation of the lpp0845 gene encoding CsrA. An apramycin antibiotic resistance cassette was introduced after the aminoacid Tyr 48 leading to a truncated CsrA protein. B) Confirmation of a significant reduced expression of the CsrA protein after disruption of its gene: 50μg of total protein of wt and csrA- grown until E (exponential) and PE (post-exponential) phase were separated on a 16% Tris/Tricine PAGE and western-blot analysis was performed with anti-CsrA antibodies. Lower panel, total protein as loading control; Numbers indicate the intensity x 1000. C) Western-blot detection of 1 μg of total protein against FlaA: Induction of the most abundant flagellar protein, FlaA is expressed in the csrA--mutant already during exponential growth but not in wild-type Legionella. Lower panel, total protein as loading control; Numbers indicate the intensity x 1000. D) The production of the secreted Legionella-pigment Pyomelanin is elevated in the csrA- mutant and is reversed by complementation with full-length csrA (csrApCsrA). E) The csrA- mutant in E phase (csrA-E) is sensitive to 100mM NaCl while the wt strain is highly resistant during exponential growth. When reaching PE phase wt and mutant show similar sensitivity to salt stress. F) The E-phase csrA- strain has higher survival rates over time under oxidative stress induced by 5mM paraquat than the wt strain. G) During E phase, csrA- (csrA-E) is more resistant to mild acidification (pH4.8) than the wt (wt-E) whereas in PE phase, no difference was observed. Each value represents the mean +/- SD of at least three independent experiments. (TIF) [file pgen.1006629.s001.tif]

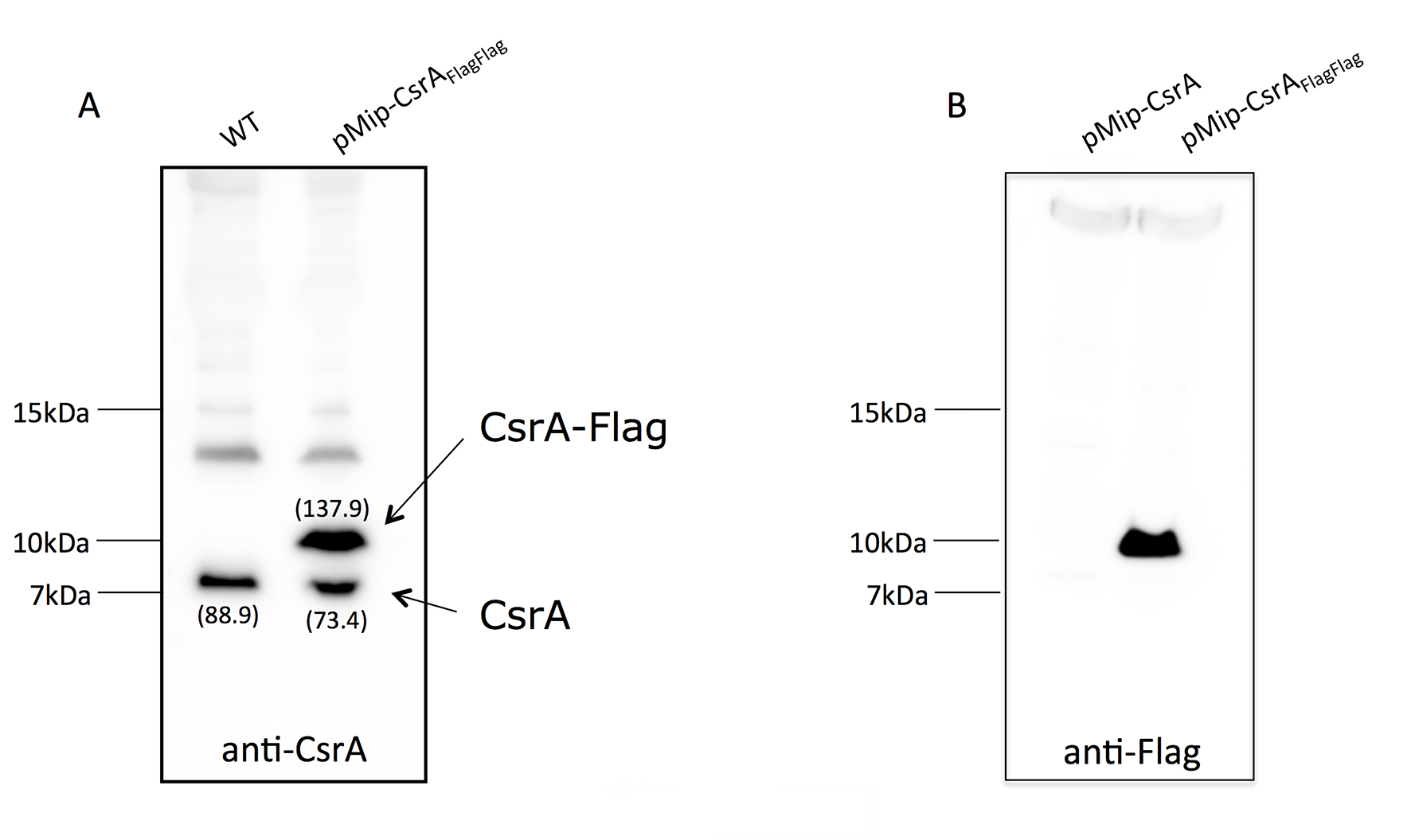

Supplement: S2 Fig — A) Separation of the crude protein extract of wt and the wt expressing the FLAG-tagged CsrA under the promoter of the Legionella mip gene on a 16% Tris/Tricine SDS-PAGE gel. Western blot analysis was performed with anti-CsrA antibodies. Numbers in brackets indicate the total band intensities (mean pixel values) as determined by GeneTools (SynGene). B) Western blot analysis showing the input of protein used for the RIPseq experiments using anti-Flag antibodies (Sigma). (TIF) [file pgen.1006629.s002.tif]

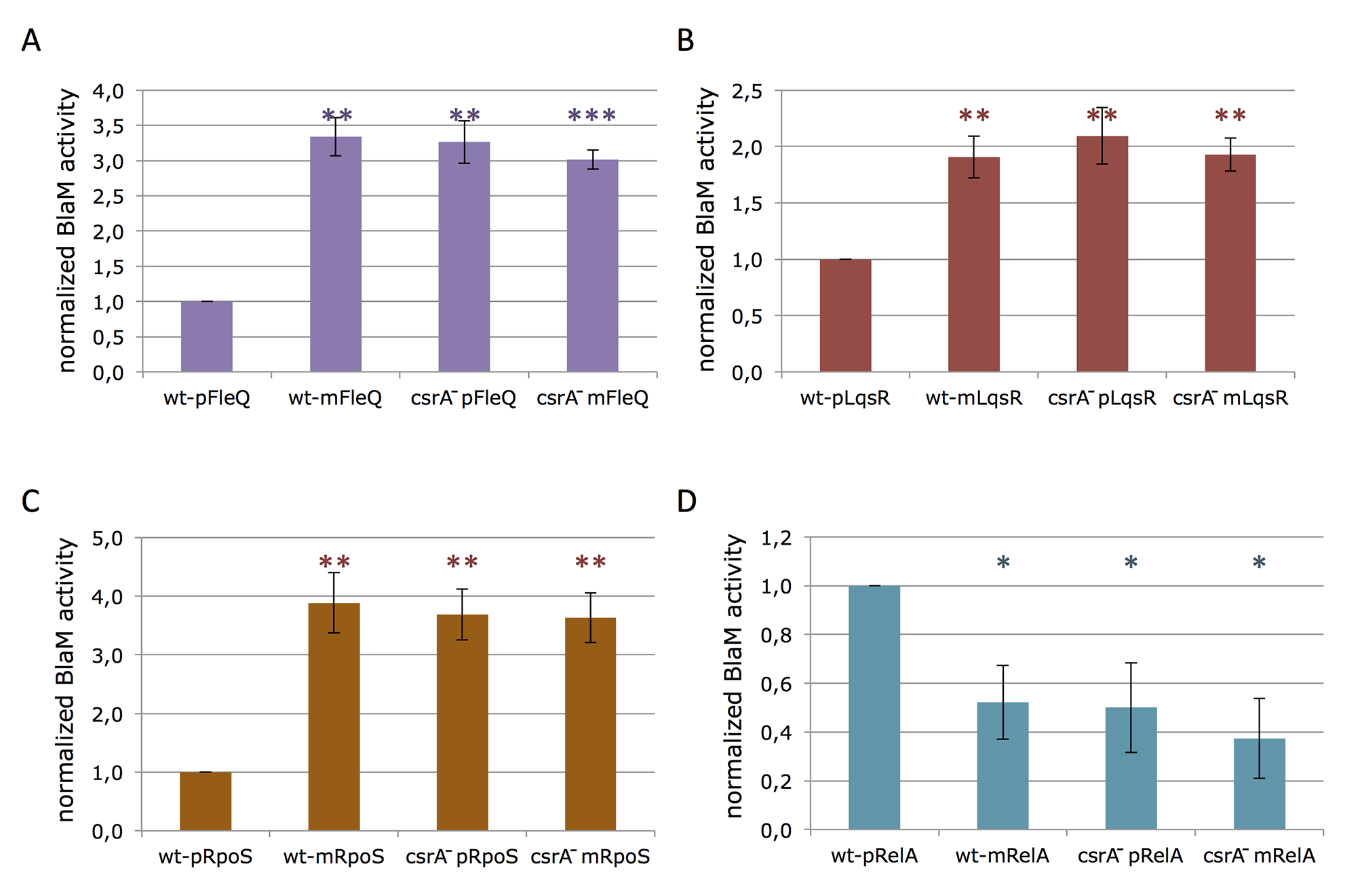

Supplement: S3 Fig — Beta-lactamase (BlaM) activity was measured from wt and csrA- L. pneumophila strains that contained the pXDC61 plasmid carrying the potential CsrA-binding region identified by RIPseq upstream of the BlaM gene (grown in minimal medium). The predicted A(N)GGA motifs were mutated (m) or not (p). A) fleQ, B) lqsR, C) relA, D) rpoS. BlaM activity of 10μg total protein from the wt or the csrA- strain was measured. Each value represents the mean +/- SD of three independent experiments. These data confirm the transcript and/or proteome results wherein FleQ is negatively regulated and RelA is positively affected by the presence of CsrA. LqsR and RpoS expression are negatively affected by CsrA according to the BlaM activity. (TIF) [file pgen.1006629.s003.tif]

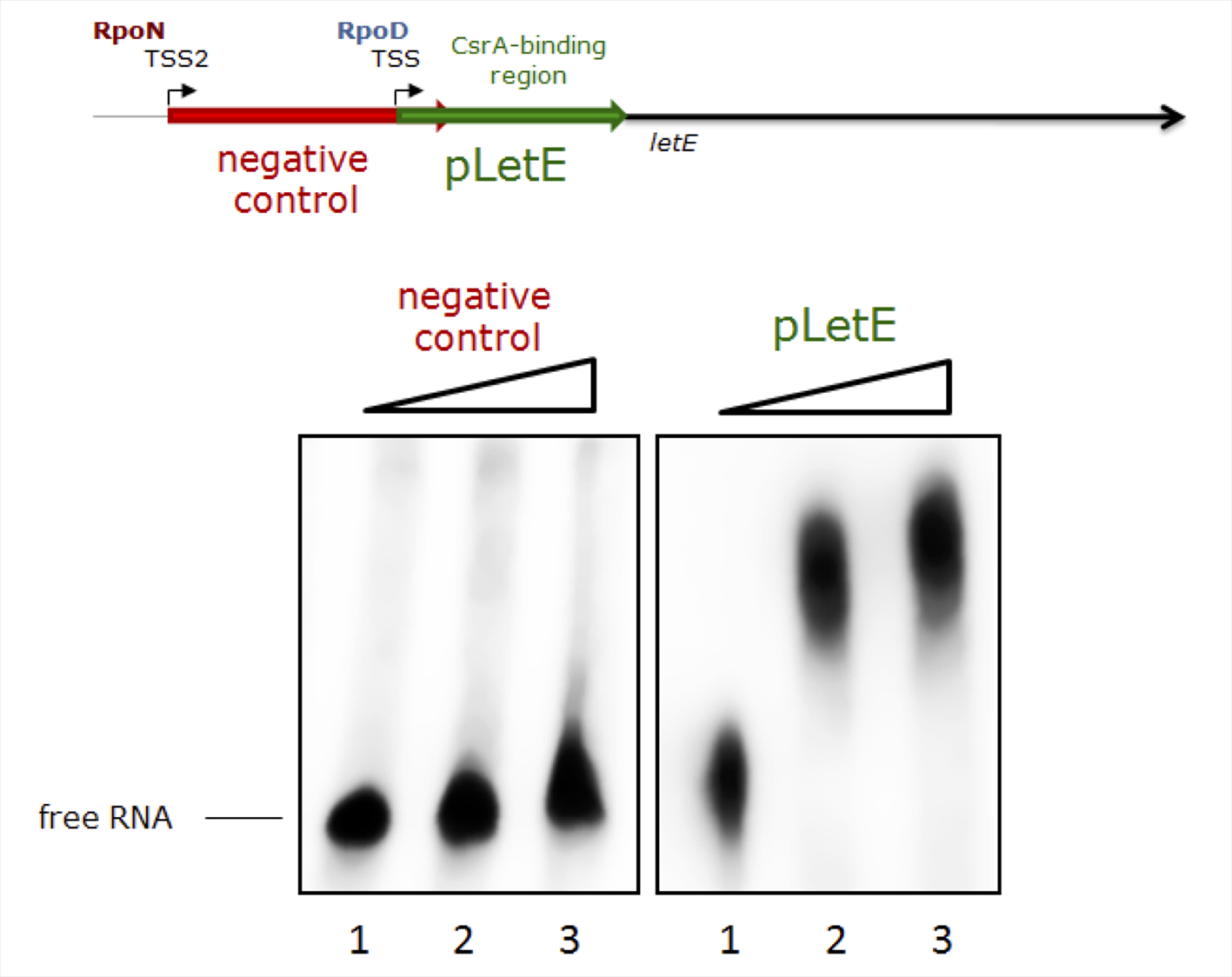

Supplement: S4 Fig — A) Schematic representation of the transcriptional unit of the lpp0602 letE gene of L. pneumophila depicts two independent TSS, the first depending on the RpoD, the other on the RpoN sigma factor. The green arrow indicates the identified CsrA-binding site, the red arrow symbolizes the region used as negative control in the EMSA with 200nM of biotinylated letE mRNA below: Lane 1: no CsrA, lane 2: 2.0 μM CsrA, lane 3: 5.0 μM CsrA. (TIF) [file pgen.1006629.s004.tif]

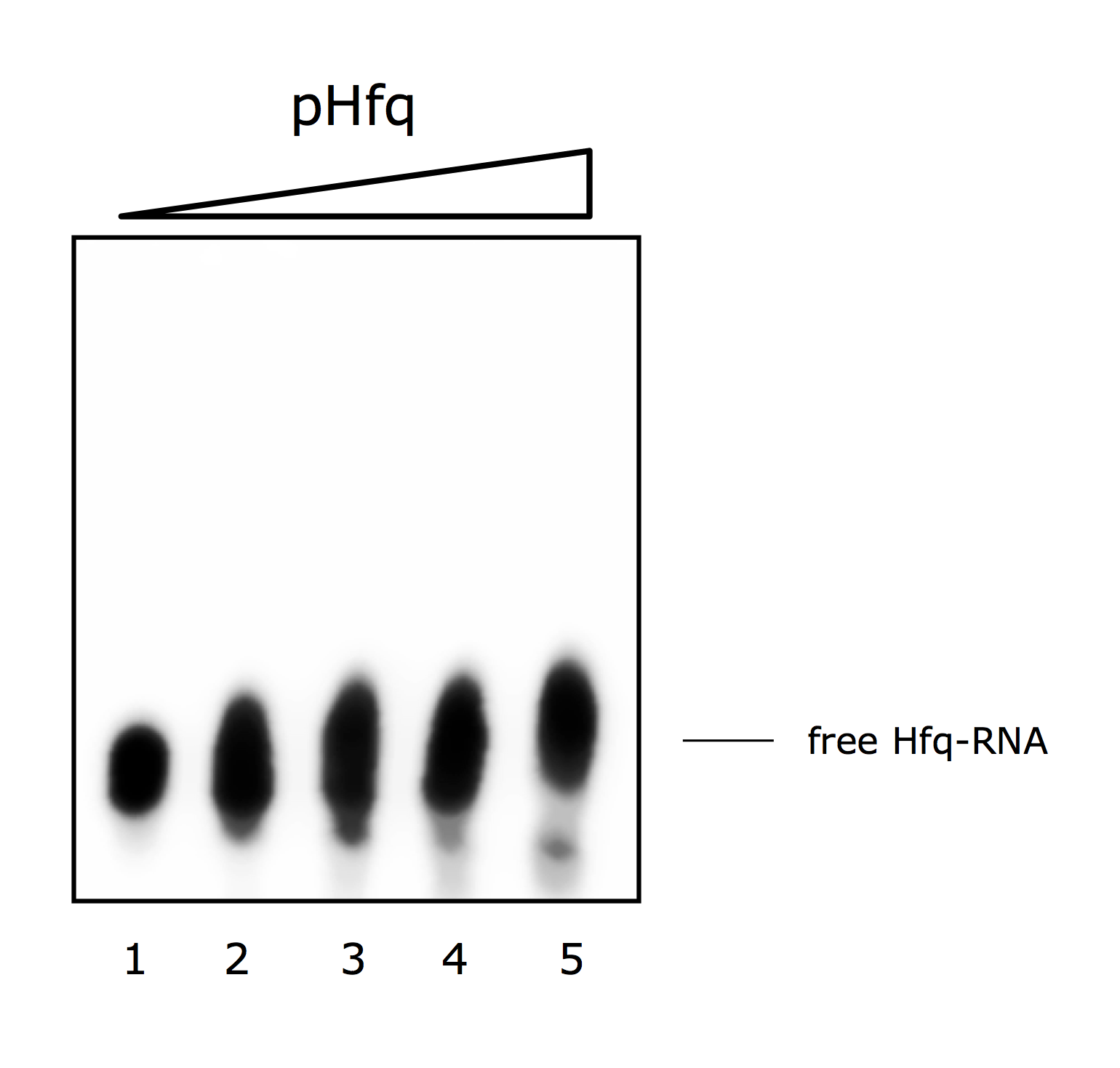

Supplement: S5 Fig — In agreement with our RIPseq data, EMSA with 200nM of biotinylated hfq RNA confirms that indeed no interaction of purified CsrA with hfq mRNA occurs in vitro even though two potential A(N)GGA motifs are present upstream and downstream of the translation start site, respectively. 200nM of biotinylated hfq mRNA and increasing concentrations of recombinant CsrA in 6% Native Tris-PAGE were used. Lane 1: no CsrA, lane 2: 1.0 μM CsrA, lane 3: 2.0 μM CsrA, lane 4: 5.0 μM CsrA, lane 5: 10 μM CsrA (TIF) [file pgen.1006629.s005.tif]

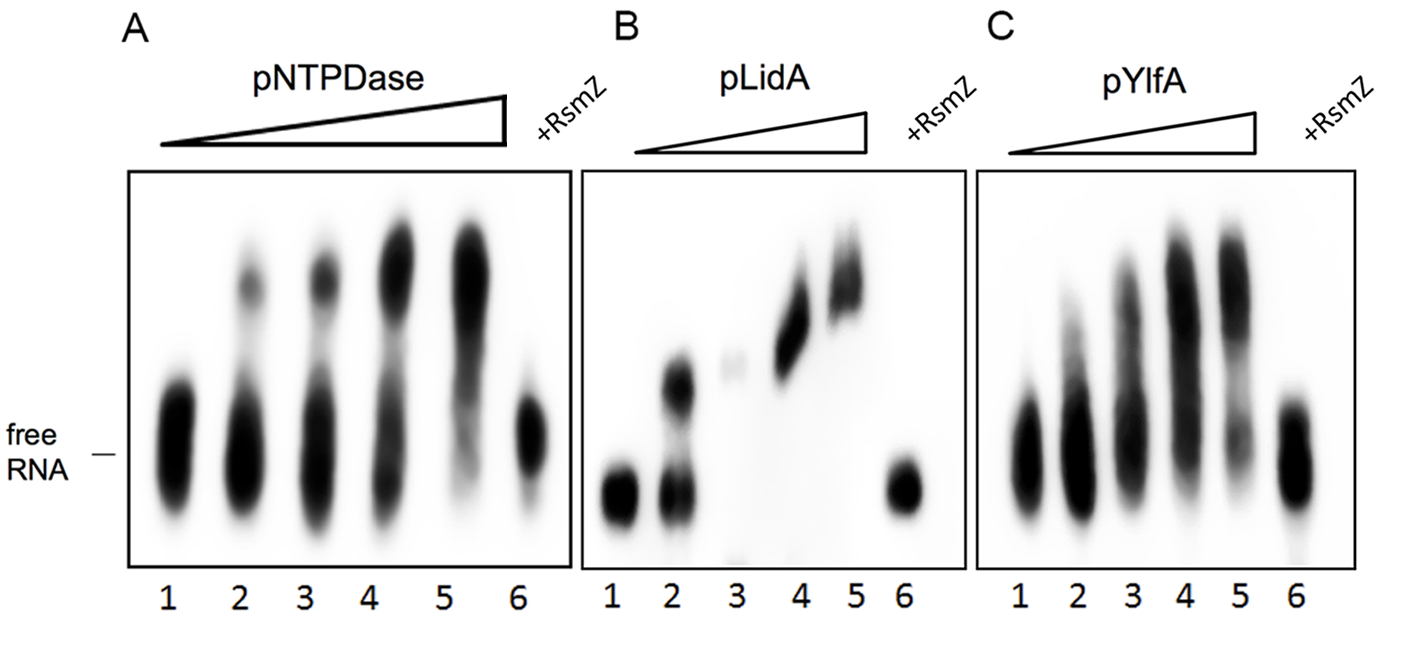

Supplement: S6 Fig — Electromobility shift assays (EMSA) with 200nM of biotinylated RNA combined with varying concentrations of purified CsrA-His in 6% Native Tris-PAGE. Lane 1: no CsrA, lane 2: 0.5 μM CsrA, lane 3: 1.0 μM CsrA, lane 4: 2.0 μM CsrA, lane 5: 5.0 μM CsrA, lane 6: 5.0 μM CsrA + 2.0 μM unlabled RsmZ. (TIF) [file pgen.1006629.s006.tif]

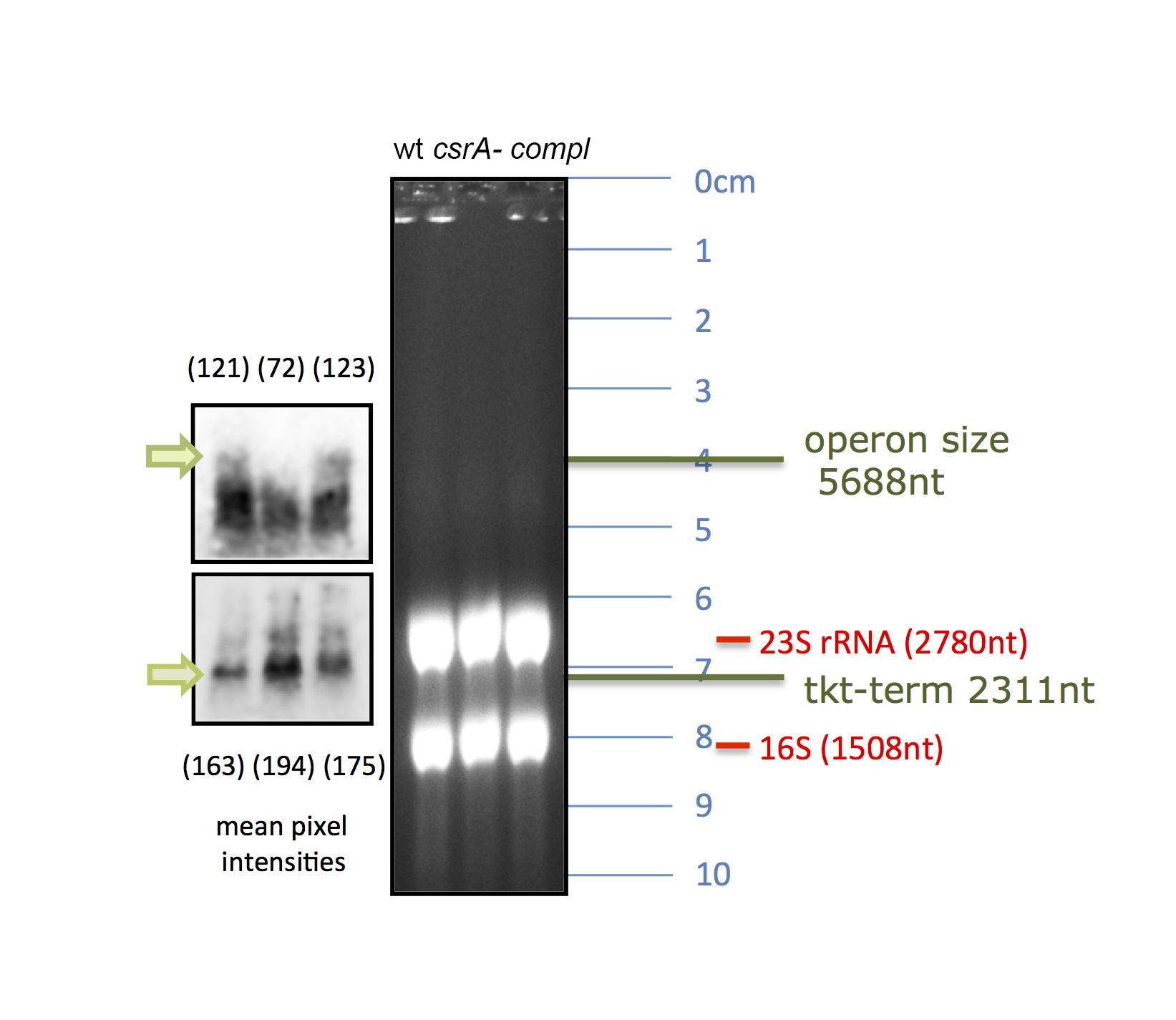

Supplement: S7 Fig — Northern Blot analysis of bacterial lysates from L. pneumophila Paris strain wt, csrA- and the complemented strains grown at the exponential phase using a gapdh probe (upper panel) for the operon transcript and a tkt probe (lower panel) for the tkt transcript. 23S and 16S RNAs signals are shown as loading controls (right panel). (TIF) [file pgen.1006629.s007.tif]

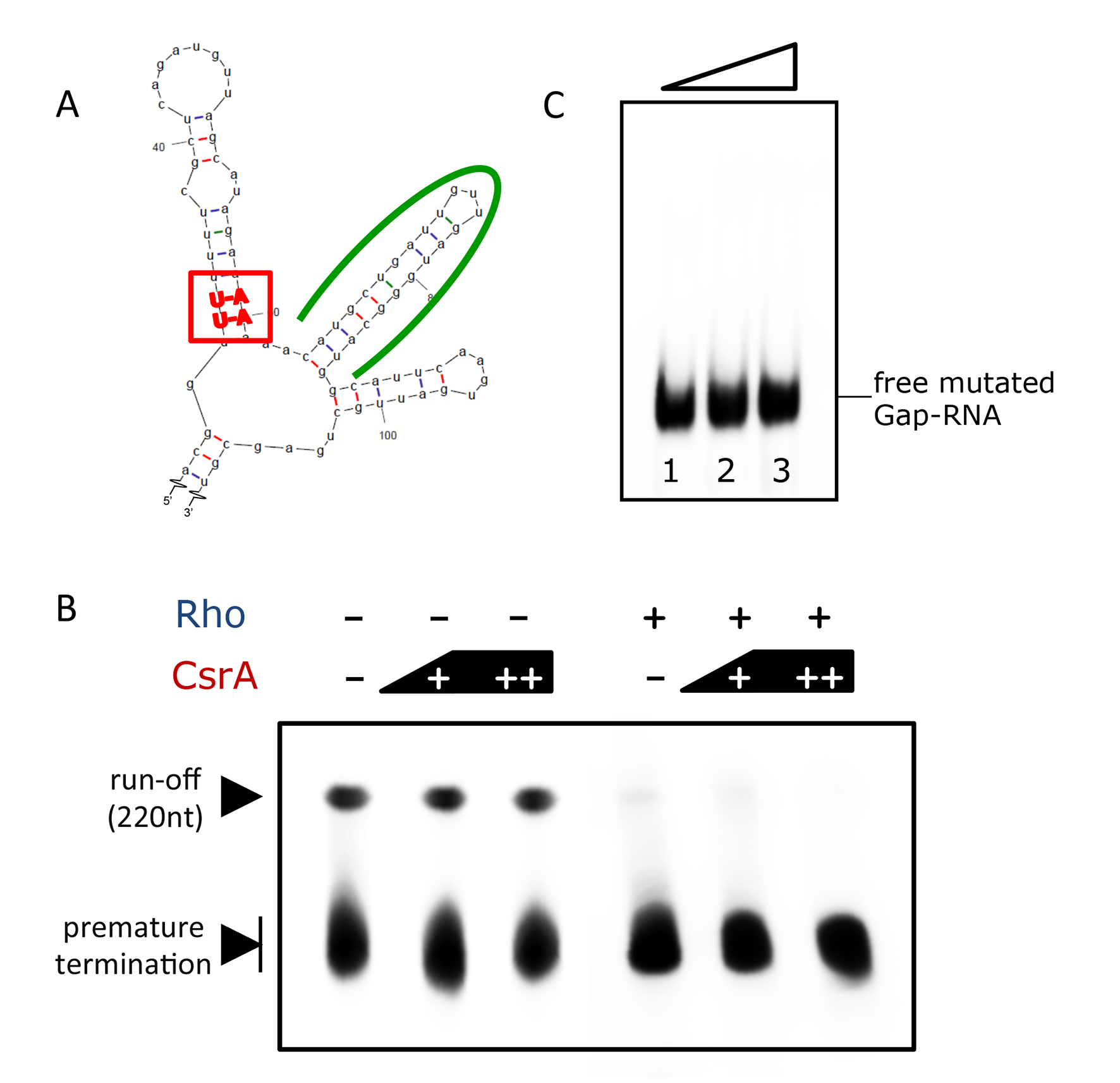

Supplement: S8 Fig — A) Mfold-prediction of the RNA secondary structure of the CsrA-binding region within the gap mRNA showing the preferred conformational status due to the double mutation. B) EMSA shift assay of the in vitro transcribed double-mutated RNA at different concentrations of CsrA, lane 1: no CsrA, lane 2: 2μM CsrA, lane 3: 5μM CsrA. C) In vitro transcription termination assay without additional purified Rho protein (left) or with 1μM Rho protein (right) at 0μM CsrA (-), 0.5μM CsrA (+) and at 1μM CsrA (++). Resulting transcripts are separated on a 10% urea-PAGE gel. (TIF) [file pgen.1006629.s008.tif]

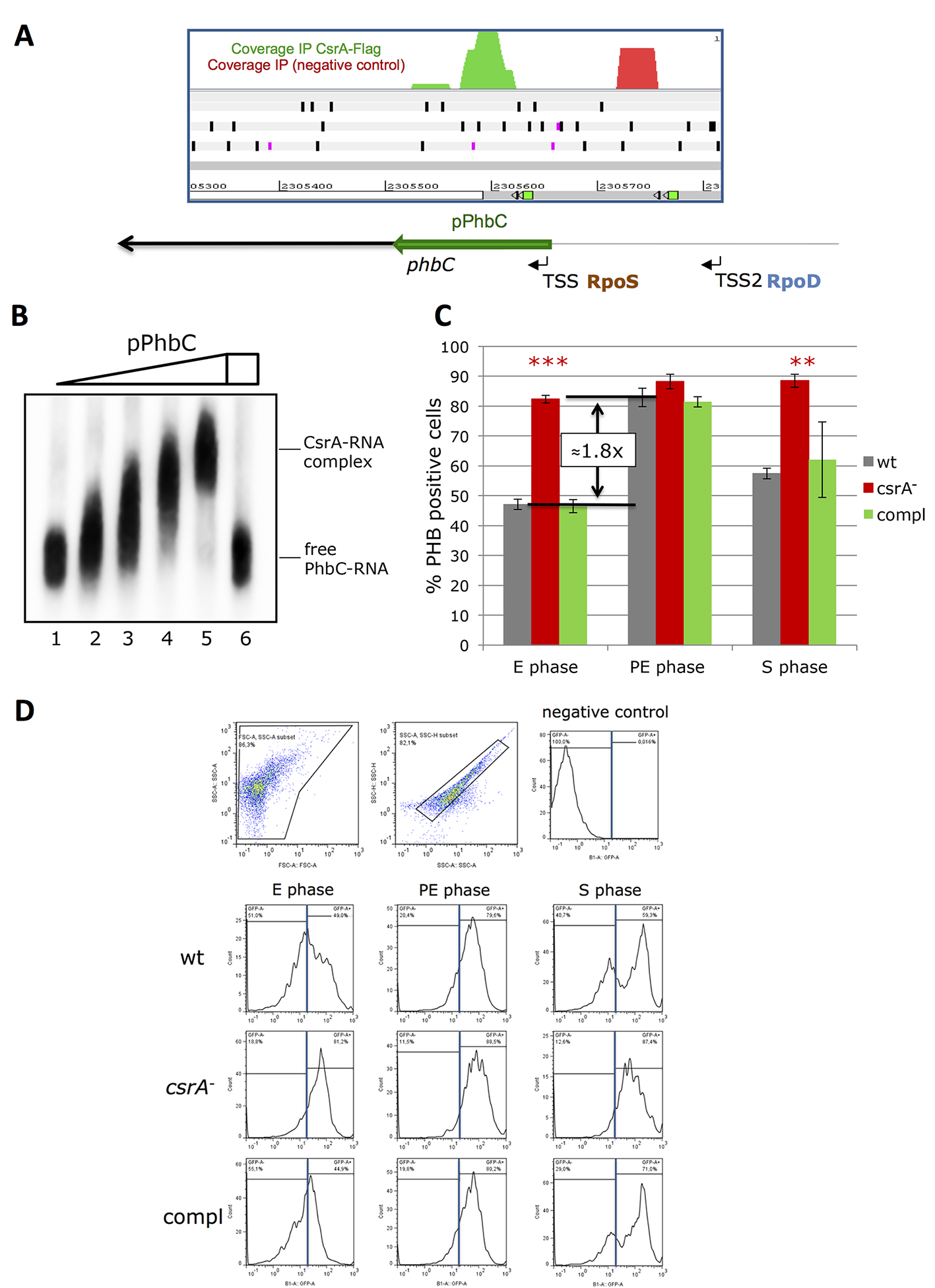

Supplement: S9 Fig — A) Schematic representation of the CsrA interaction region with the PhbC (Lpp2038)-transcript located in the 5'UTR/RBS. Above, coverage of the reads gained from a representative RIPseq experiment compared to the negative control. B) EMSA with 200nM of biotinylated phbC mRNA, lane 1: no CsrA, lane 2: 2.0 μM CsrA, lane 3: 5.0 μM CsrA. C) Staining and quantification of the PHB positive cells with Bodipy 493/503 by flow cytometry during exponential (E), post-exponential (PE) and stationary phase comparing wt and CsrA-mutant. Each value represents the mean +/- SD of three independent experiments. In absence of CsrA, the amount of PHB is significantly increased during E and S phase indicating a higher synthesis or reduced usage of PHB in the mutant bacteria than the wt. D) Gating strategy for flow cytometry analysis of Legionella. Representative scatter plot distribution of PHB fluorescence for Bodipy 493/503 stained wt and a csrA- and complemented strain during different growth phases based on FSC-A vs SSC-A and SSC-A vs SSC-H subsets to discriminate single bacteria. The fluorescence data was collected using a 530± 30 nm band pass filter; the threshold of PHB positive cells was determined by unstained Legionella cells (negative control). (TIF) [file pgen.1006629.s009.tif]

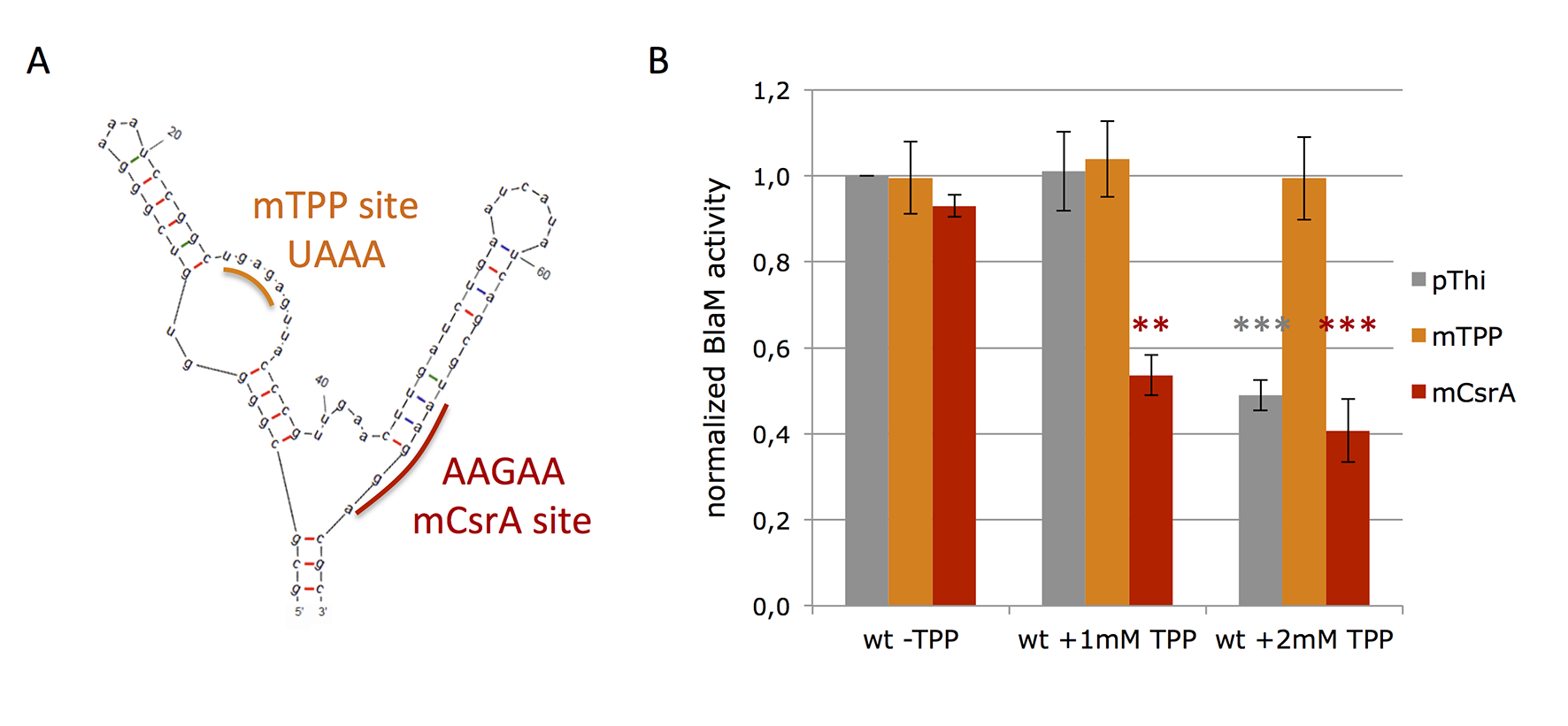

Supplement: S10 Fig — A) Schematic representation of the thi-operon in L. pneumophila indicating the region that was mutated for the BlaM activity assay. B) Normalized BlaM activity at no, 1mM and 2mM of extracellular TPP concentration in L. pneumophila grown in a minimal medium. BlaM activity is significantly reduced at 1mM TPP when the CsrA-binding site was mutated (mCsrA). Similarly, the activity dropped significantly for the non-mutated thi-element at 2mM TPP, but the mutation of the thiamine-binding site (mTPP) abolished the dependency on TPP completely. (TIF) [file pgen.1006629.s010.tif]

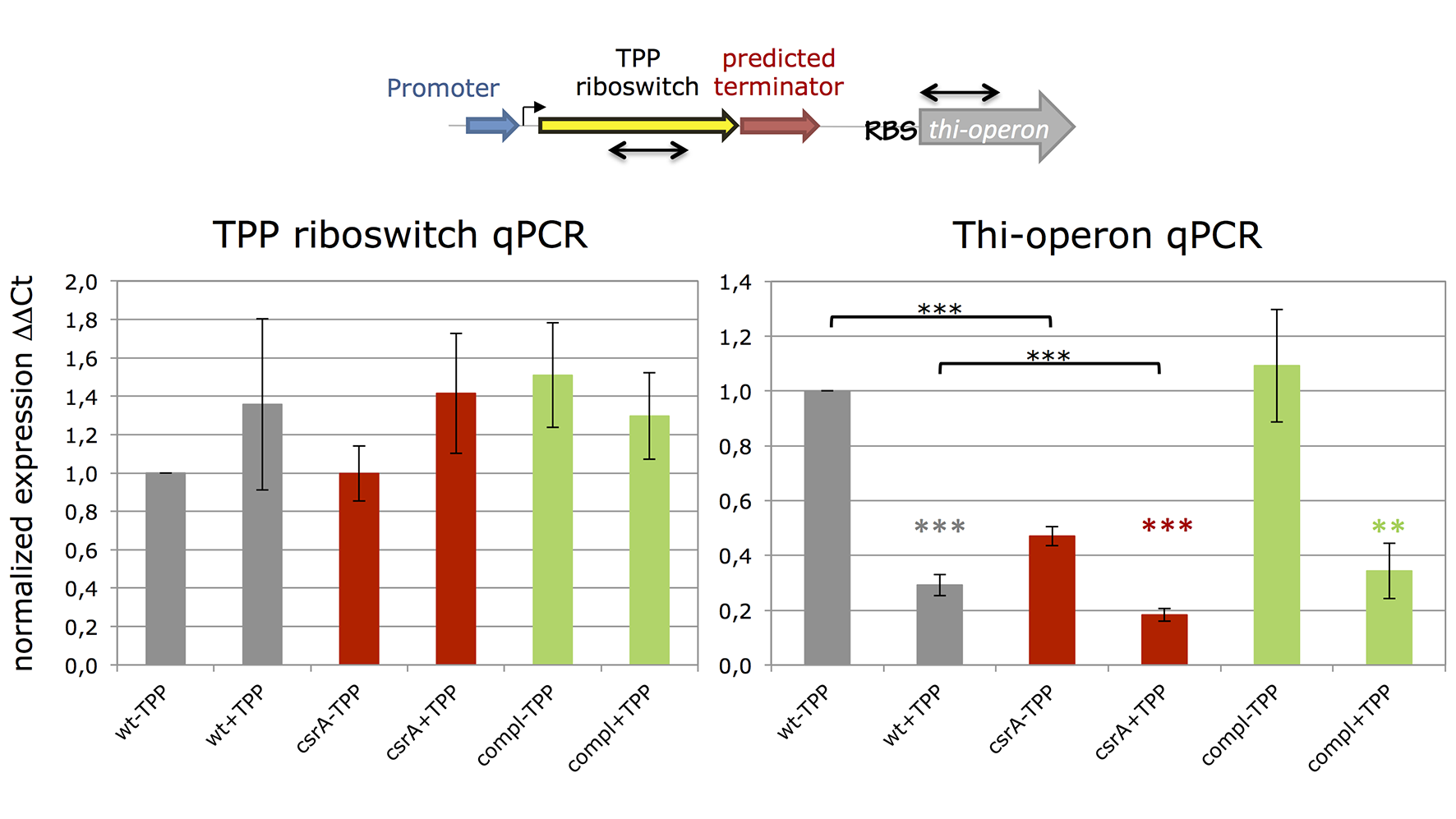

Supplement: S11 Fig — A) Schematic representation of the thi-operon in L. pneumophila and the region used for qPCR amplification. B) The transcript level of the TPP riboswitch region is not affected in presence of 2 mM TPP and CsrA (left) whereas downstream of the predicted terminator region the transcript level is lower in presence of 2 mM TPP and in the csrA- background, in presence and absence of TPP compared to the wt (right). Complementation of csrA- strain with the csrA gene restored the transcript levels to wt level. (TIF) [file pgen.1006629.s011.tif]

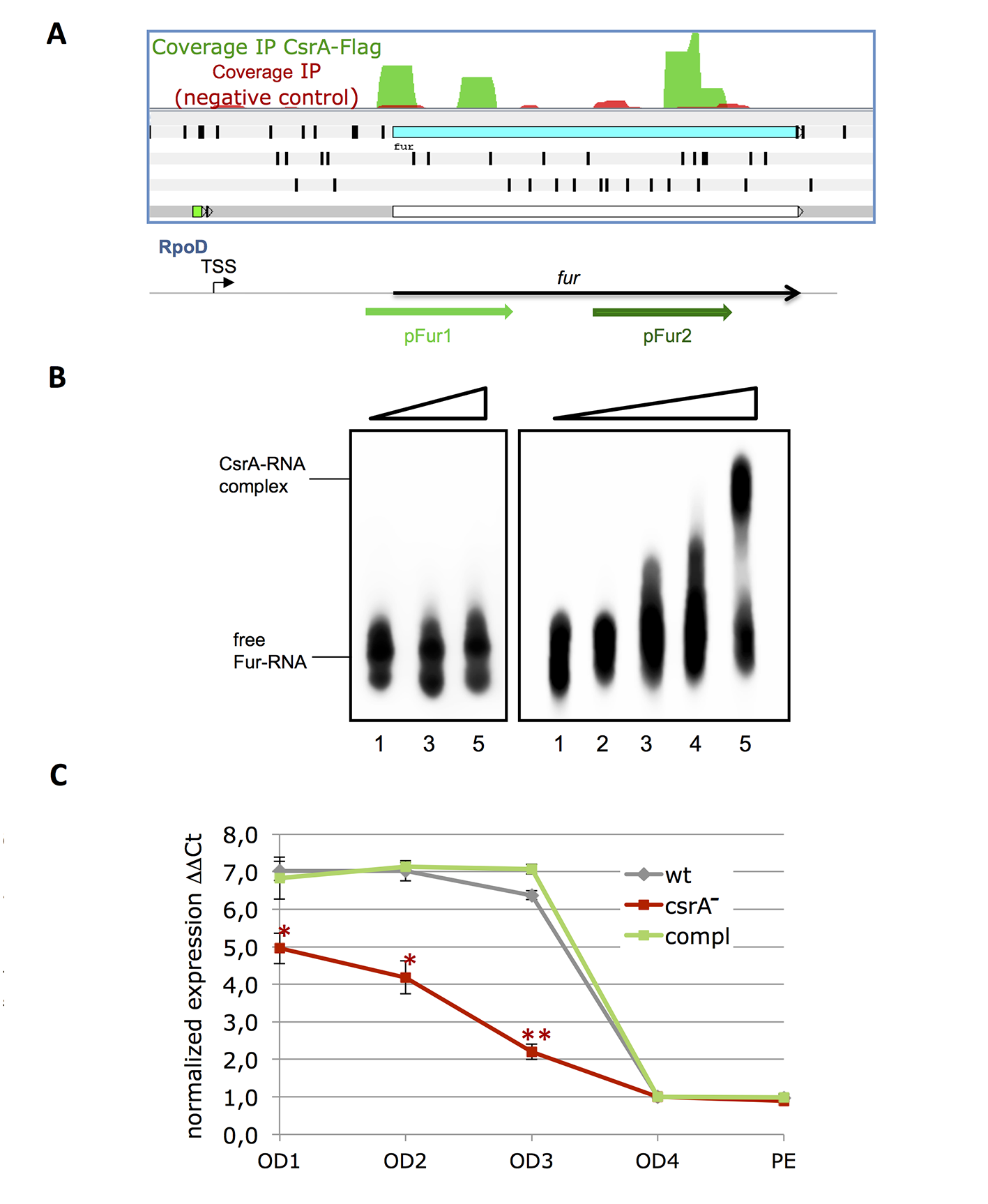

Supplement: S12 Fig — A) Schematic representation of the fur gene organisation including the potential CsrA-binding site illustrated by the coverage of the reads obtained from a representative RIPseq experiment. B) To analyse the interaction, two independent EMSAs with 200nM of biotinylated RNA of region Fur1 mRNA and Fur2 mRNA together with purified CsrA were performed. Only the position inside the CDS (Fur2), but not the region around the RBS (Fur1) reacted with CsrA in vitro.: Lane 1: no CsrA, lane 2: 0.5 μM CsrA, lane 3: 1.0 μM CsrA, lane 4: 2.0 μM CsrA, lane 5: 5.0 μM CsrA. C) RNA stability assay of the Fur-transcript in absence of CsrA (red) and during over-expression of CsrA (green) compared to wt. qRT-PCR was performed from BYE cultures after addition of 100μM rifampicine for 0, 5, 10 and 20 min. Each value represents the mean +/- SD of at least two independent experiments. RNA half life was calculated from the average of the time points compared to the value at t0 according to t1/2 = t*ln(2)/ln(N0/N(t)). (TIF) [file pgen.1006629.s012.tif]

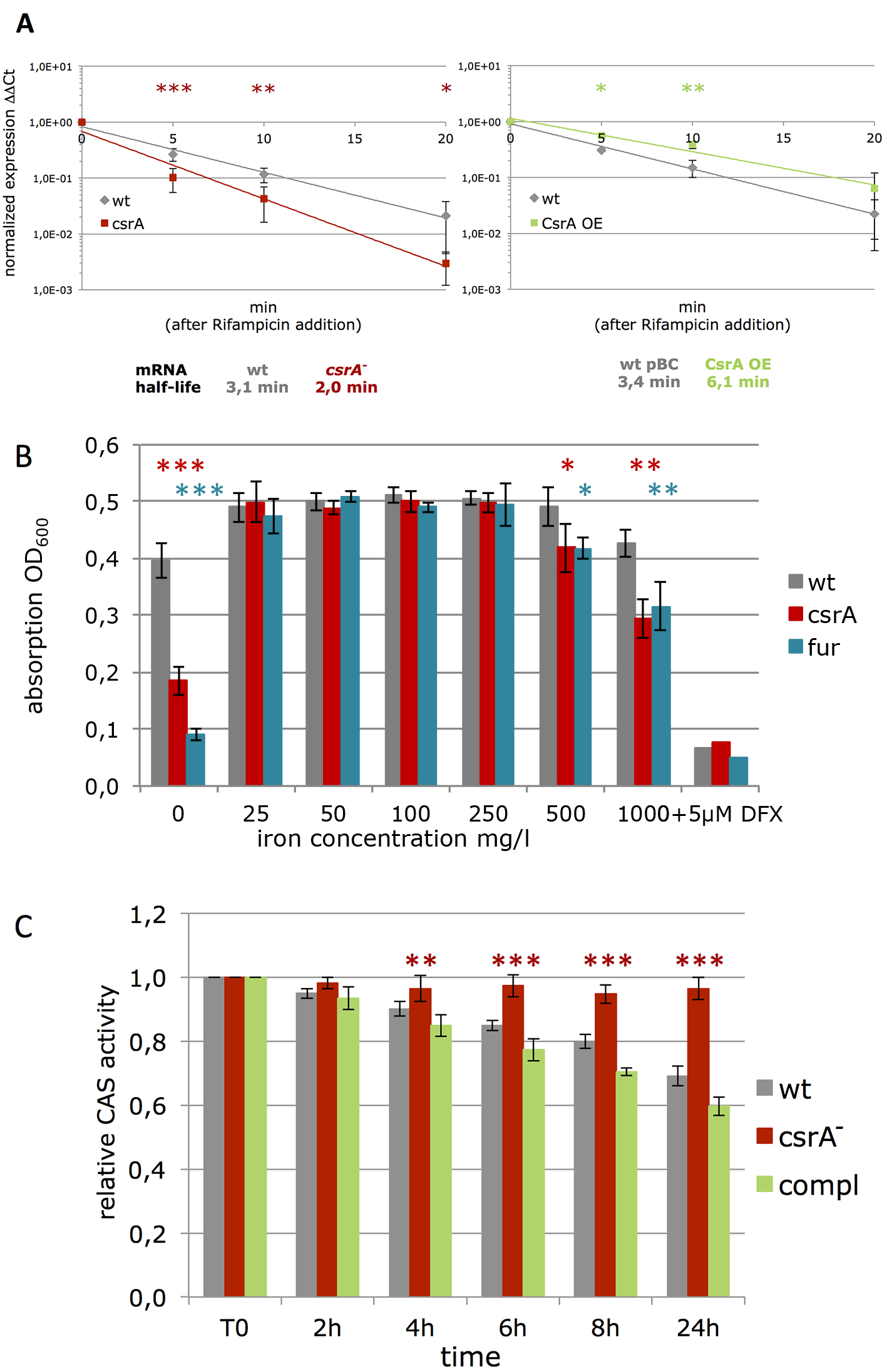

Supplement: S13 Fig — A) qRT-PCR results of the fur transcript at different growth stages (OD) of the wt and csrA- strain showed lower expression levels of the fur gene in E-phase (OD1-3) in absence of CsrA. No differences were noticed during the transition (OD4) and in the PE-phase. Complementation restored the phenotype. B) A growth defect of the csrA- and the Δfur mutant compared to the wt was observed at very low (0 μM) and high (>500 μM) iron concentration added to the minimal medium. Similarly, the addition of the iron chelator, DFX, abolished the growth of all strains. C) A CAS assay was performed to monitor siderophore secretion. Reduction of free iron was measured as described in M&M. Our results showed that in the csrA- strain the amount of free iron was stable over time indicating that no iron siderophore complexes were present. In contrast, a clear reduction of free iron was found for the wt and the complemented strain suggesting siderophore secretion in both. (TIF) [file pgen.1006629.s013.tif]
