# Supplementary material for: The Legionella pneumophila genome evolved to accommodate multiple regulatory mechanisms controlled by the CsrA-system
Source: PLoS Genet. 2017 Feb 17;13(2):e1006629. doi: 10.1371/journal.pgen.1006629 (PMC5338858; doi:10.1371/journal.pgen.1006629)
Supplement: S1 Table — (DOCX) [file pgen.1006629.s014.docx]

**Table S1: Differentially expressed genes according to transcriptome analyses of wt and *csrA*^-^**

A) Up-regulated during exponential growth phase in the *csrA***^-^**  strain (p<0.05, 65 up-regulated also in proteome analyses)

| **gene.ID** | **description** | **FC** |
| --- | --- | --- |
| *lpp0001* | Chromosomal replication initiator protein DnaA | 1,69 |
| *lpp0010* | GTP-binding protein Hflx | 1,53 |
| *lpp0012* | Hypothetical protein conserved within Legionellae | 2,96 |
| *lpp0013* | Pirin-related protein | 1,88 |
| *lpp0023* | Membrane protein of unknown function | 1,96 |
| *lpp0026* | Amino acid permease family protein | 1,99 |
| *lpp0033* | Hypothetical protein | 1,52 |
| *lpp0052* | Hypothetical protein | 3,72 |
| *lpp0094* | Dot/Icm T4SS effector | 1,96 |
| *lpp0112* | Two component response regulator protein | 3,12 |
| *lpp0138* | Cytochrome c553 | 1,54 |
| *lpp0202* | Ankyrin repeat protein | 1,63 |
| *lpp0263* | Putative dihydroxyacetone kinase containing a DAK2 and EDD domain | 1,70 |
| *lpp0266* | Predicted membrane protein (DUF2177) | 1,56 |
| *lpp0291* | 3-ketoacyl-(acyl-carrier-protein) reductase | 1,60 |
| *lpp0307* | Hydrolase, alpha/beta family | 1,71 |
| *lpp0308* | Betaine-aldehyde dehydrogenase | 1,99 |
| *lpp0309* | 4-aminobutyrate aminotransferase | 1,86 |
| *lpp0314* | Pyridine nucleotide-disulfide oxidoreductase | 1,78 |
| *lpp0317* | Hypothetical protein | 1,57 |
| *lpp0318* | Arsenate reductase, arsc family | 1,75 |
| *lpp0341* | CorA-like Mg2+ and Co2+ transport protein | 1,77 |
| *lpp0351* | Regulatory protein (EAL domain) | 2,11 |
| *lpp0359* | NAD-dependent formate dehydrogenase | 3,18 |
| *lpp0446* | Ribosomal protein S6 modification enzyme | 1,62 |
| *lpp0452* | LemA-family protein | 1,80 |
| *lpp0453* | Heat shock protein HtpX | 1,67 |
| *lpp0493* | Cold shock-like protein CspD | 6,57 |
| *lpp0506* | Hypothetical protein | 2,65 |
| *lpp0507* | IcmT | 1,77 |
| *lpp0508* | IcmS | 2,09 |
| *lpp0509* | IcmR | 1,53 |
| *lpp0514* | IcmM/DotJ | 1,50 |
| *lpp0515* | IcmL/DotI | 1,82 |
| *lpp0516* | IcmK/DotH | 1,63 |
| *lpp0517* | IcmE/DotG | 1,58 |
| *lpp0518* | IcmG/DotF | 1,63 |
| *lpp0519* | IcmC/DotE | 1,75 |
| *lpp0520* | IcmD/DotP | 1,70 |
| *lpp0521* | IcmJ/DotN | 1,51 |
| *lpp0560* | ABC-type amino acid transporter, bacterial periplasmic component | 1,96 |
| *lpp0564* | Hypothetical protein | 1,84 |
| *lpp0602* | Transmission trait enhancer protein LetE | 1,99 |
| *lpp0608* | Outer membrane lipoprotein LoLB | 1,85 |
| *lpp0616* | Formamidopyrimidine-DNA glycosylase | 1,59 |
| *lpp0621* | Acetoacetyl-CoA reductase | 2,86 |
| *lpp0622* | Polyhydroxyalkanoate synthesis regulator PhaR | 2,15 |
| *lpp0623* | Phasin PhaP-like protein | 1,93 |
| *lpp0634* | Protein of unknown function (DUF47) | 1,54 |
| *lpp0639* | Hypothetical protein | 1,60 |
| *lpp0641* | BMFP-like protein | 1,59 |
| *lpp0663* | Zn-dependent alcohol dehydrogenase | 1,63 |
| *lpp0711* | Outer membrane protein, OmpA family | 1,58 |
| *lpp0723* | Uncharacterized protein conserved in bacteria (DUF2063) | 1,61 |
| *lpp0724* | Protein of unknown function (DUF692) | 1,75 |
| *lpp0725* | Predicted integral membrane protein (DUF2282) | 4,84 |
| *lpp0727* | NADH dehydrogenase, FAD-containing subunit | 2,15 |
| *lpp0728* | Acetoacetate decarboxylase | 3,84 |
| *lpp0736* | Alpha/beta hydrolase family | 1,55 |
| *lpp0738* | ABC-type multidrug transport system, ATPase component | 1,53 |
| *lpp0802* | Uncharacterized protein conserved in bacteria (DUF2147) | 4,35 |
| *lpp0808* | Zinc finger-domain containing protein (CDGSH-type) | 1,53 |
| *lpp0809* | Signal transduction protein, GGDEF domain | 1,65 |
| *lpp0829a* | Hypothetical protein | 1,85 |
| *lpp0829b* | Hypothetical protein | 1,95 |
| *lpp0855* | Macrophage infectivity potentiator Mip | 1,54 |
| *lpp0865* | Acyl-CoA dehydrogenase | 1,97 |
| *lpp0866* | Choloylglycine hydrolase, Ntn hydrolase superfamily | 3,00 |
| *lpp0872* | Competence protein ComEA helix-hairpin-helix repeat region | 4,96 |
| *lpp0879* | ATP-dependent Clp protease adaptor protein ClpS | 1,93 |
| *lpp0880* | ATP-dependent Clp protease ATP-binding subunit ClpA | 1,69 |
| *lpp0904* | ABC-type transport system | 1,76 |
| *lpp0937* | NAD(P) transhydrogenase beta subunit PntB | 1,88 |
| *lpp0938* | NAD(P) transhydrogenase alpha2 subunit PntAB2 | 1,98 |
| *lpp0939* | NAD(P) transhydrogenase alpha subunit PntA | 1,98 |
| *lpp0953* | FAD-dependent oxidoreductase | 1,92 |
| *lpp0962* | Conserved hypothetical protein | 4,65 |
| *lpp0963* | Protein of unknown function | 4,18 |
| *lpp0964* | Conserved protein of unknown function | 2,58 |
| *lpp0969* | Flagellar biosynthesis anti-sigma factor FlgM | 2,21 |
| *lpp0970* | Flagellar basal body P-ring biosynthesis protein FlgA | 2,47 |
| *lpp0975* | AdoMet-dependent methyltransferase MraW | 1,59 |
| *lpp0982* | Substrate of the Dot/Icm secretion system MavT | 1,53 |
| *lpp0983* | Hypothetical gene | 2,03 |
| *lpp0989* | Tfp pilus assembly protein PilM | 1,50 |
| *lpp0990* | Tfp pilus assembly protein PilN | 1,57 |
| *lpp0991* | Tfp pilus assembly protein PilO | 1,65 |
| *lpp0992* | Tfp pilus assembly protein PilP | 1,60 |
| *lpp0997* | Universal stress protein UspA | 1,79 |
| *lpp1002* | LidA effector protein | 1,88 |
| *lpp1011* | Integral membrane protein YccA | 1,66 |
| *lpp1025* | Substrate of the Dot/Icm secretion system | 4,04 |
| *lpp1113* | Protein of unknown function | 1,93 |
| *lpp1137* | Protein of unknown function (DUF4349) | 1,54 |
| *lpp1146* | Hypothetical protein | 2,16 |
| *lpp1151* | Hypothetical protein | 1,73 |
| *lpp1168* | Hypothetical protein | 1,65 |
| *lpp1170* | Signal transduction protein, GGDEF and EAL domains | 2,04 |
| *lpp1177* | Bacterial transcription regulatory protein, AsnC family | 10,71 |
| *lpp1224* | Flagellar basal-body rod protein FlgB | 6,87 |
| *lpp1225* | Flagellar basal-body rod protein FlgC | 5,64 |
| *lpp1226* | Flagellar basal-body rod modification protein FlgD | 5,81 |
| *lpp1227* | Flagellar hook protein FlgE | 6,10 |
| *lpp1228* | Flagellar biosynthesis protein FlgF | 4,80 |
| *lpp1229* | Flagellar biosynthesis protein FlgG | 7,25 |
| *lpp1230* | Flagellar L-ring protein precursor FlgH | 7,12 |
| *lpp1232* | Flagellar biosynthesis protein FlgI | 4,06 |
| *lpp1233* | Flagellar hook-associated protein 1 | 3,16 |
| *lpp1234* | Flagellar hook-associated protein FlgI | 3,02 |
| *lpp1247* | RNA polymerase sigma factor RpoS | 1,80 |
| *lpp1294* | Flagellin FlaA | 2,88 |
| *lpp1310* | Enhanced entry protein EnhC | 1,88 |
| *lpp1324* | Global DNA-binding transcriptional regulator Fis2 | 3,42 |
| *lpp1340* | Protein of unknown function | 3,65 |
| *lpp1406* | Phosphatidylethanolamine-binding protein PEBP | 2,32 |
| *lpp1410* | Major facilitator superfamily (MFS) protein, putative emrb/qaca subfamily | 1,86 |
| *lpp1456* | Hypothetical protein | 1,55 |
| *lpp1471* | Legionella type I secretion system protein Z | 1,63 |
| *lpp1473* | Legionella type I secretion system protein B | 1,61 |
| *lpp1554* | Multifunctional fatty acid oxidation complex, alpha subunit FadJ | 1,96 |
| *lpp1605* | SnoaL-like polyketide cyclase | 1,51 |
| *lpp1640* | Hypothetical protein | 1,54 |
| *lpp1652* | Hypothetical protein | 2,71 |
| *lpp1689* | Septum formation inhibitor-activating Atpase MinD | 1,84 |
| *lpp1690* | Cell division topological specificity factor MinE | 1,79 |
| *lpp1691* | Acyl-CoA dehydrogenase FadE | 1,51 |
| *lpp1722* | Flagellar assembly protein FliH | 2,10 |
| *lpp1723* | Flagellar motor switch protein FliG | 1,75 |
| *lpp1724* | Flagellar MS-ring protein FliF | 2,51 |
| *lpp1725* | Flagellar hook-basal body protein FliE | 2,17 |
| *lpp1726* | Response regulator FleR | 2,27 |
| *lpp1727* | Sensor histidine kinase FleS | 1,68 |
| *lpp1738* | Ribonucleoside-diphosphate reductase, alpha subunit Rir1 | 1,59 |
| *lpp1739* | Ribonucleoside-diphosphate reductase, beta subunit Rir2 | 1,58 |
| *lpp1743* | Protein of unknown function (DUF2802) | 2,58 |
| *lpp1744* | Flagellar motor protein MotB | 2,05 |
| *lpp1745* | Flagellar motor protein MotA | 2,70 |
| *lpp1746* | Flagellar biosynthesis sigma factor FliA | 3,12 |
| *lpp1747* | Anti-activator of flagellar biosynthesis FleN | 3,60 |
| *lpp1748* | Flagellar biosynthesis regulator FlhF | 5,10 |
| *lpp1749* | Flagellar biosynthesis protein FlhA | 1,86 |
| *lpp1750* | Flagellar biosynthesis protein FlhB | 1,97 |
| *lpp1751* | Flagellar biosynthesis protein FliR | 2,90 |
| *lpp1753* | Flagellar biosynthesis protein FliP | 3,21 |
| *lpp1754* | Flagellar biosynthesis protein FliO | 2,77 |
| *lpp1755* | Flagellar motor switch protein FliN | 5,55 |
| *lpp1756* | Flagellar motor switch protein FliM | 3,97 |
| *lpp1788* | Acetyl-CoA acetyltransferase | 1,60 |
| *lpp1799* | Hypothetical protein | 1,62 |
| *lpp1800* | Conserved protein of unknown function | 1,54 |
| *lpp1826* | DNA-binding protein HU-beta | 1,78 |
| *lpp1856* | Alpha/beta hydrolase | 4,63 |
| *lpp1883* | Glutathionine S-transferase | 2,87 |
| *lpp1890* | Tfp pilus assembly protein PilA | 1,87 |
| *lpp1936* | Hypothetical protein | 3,27 |
| *lpp1957* | Hypothetical protein | 2,78 |
| *lpp1995* | Tfp pilus assembly protein, pilus retraction ATPase PilT | 1,57 |
| *lpp2010* | Uroporphyrinogen decarboxylase HemE | 1,59 |
| *lpp2067* | L,D-transpeptidase catalytic domain | 1,89 |
| *lpp2092* | Substrate of the Dot/Icm system, Sid-like protein SdeC | 1,95 |
| *lpp2150* | Hypothetical protein | 1,94 |
| *lpp2163* | Arginase/histone deacetylase-like superfamily | 1,77 |
| *lpp2195* | Protein of unknown function (DUF1688) | 1,50 |
| *lpp2209* | Membrane protein of unkown function | 4,80 |
| *lpp2243* | Hypothetical protein | 1,51 |
| *lpp2245a* | Transposase, IS4 family | 1,75 |
| *lpp2246* | YlfA effector protein | 2,11 |
| *lpp2259* | Hypothetical protein | 1,58 |
| *lpp2264* | 3-hydroxybutyrate dehydrogenase | 1,93 |
| *lpp2265* | Patatin-like phospholipase | 1,87 |
| *lpp2266* | Flagellar motor protein MotA | 1,51 |
| *lpp2272* | Eukaryotic-like sugar 1,4-lactone oxidase domain | 1,84 |
| *lpp2276* | Substrate of the Dot/Icm secretion system | 2,41 |
| *lpp2293* | Hypothetical protein | 1,73 |
| *lpp2297* | Cu/Zn superoxide dismutase | 1,93 |
| *lpp2302* | ADP-ribose diphosphatase NudE | 1,51 |
| *lpp2304* | Aspartyl/glutamyl-tRNA (Asn/Gln) amidotransferase and related amidases | 1,53 |
| *lpp2322* | Acetoacetyl-CA reductase | 3,66 |
| *lpp2327* | Hypothetical protein | 3,38 |
| *lpp2329* | F0F1-type ATP synthase, epsilon subunit | 1,96 |
| *lpp2332* | F0F1-type ATP synthase, subunit C | 1,67 |
| *lpp2333* | F0F1-type ATP synthase, subunit B | 1,95 |
| *lpp2336* | Hypothetical protein | 1,75 |
| *lpp2337* | Iron-sulfur binding ferredoxin reductase (FNR) protein | 1,54 |
| *lpp2354* | Domain of unknown function (DUF4156) | 1,68 |
| *lpp2355* | Signal transduction protein, GGDEF domain | 1,58 |
| *lpp2362* | Chemiosmotic efflux system B protein B | 1,84 |
| *lpp2363* | Chemiosmotic efflux system B protein C | 1,71 |
| *lpp2453* | Amino acid transporter | 1,59 |
| *lpp2465* | Bacterial protein of unknown function (DUF945), hypothetical virulence protein | 1,80 |
| *lpp2482* | Hypothetical protein | 2,77 |
| *lpp2494* | Malonate decarboxylase, delta and beta subunit MdcCD | 1,95 |
| *lpp2501* | Uncharacterized conserved protein, Cupin 2 superfamily | 2,21 |
| *lpp2502* | Protein of unknown function (DUF1311) | 2,00 |
| *lpp2506* | NADPH-dependent FMN reductase | 1,76 |
| *lpp2507* | Glutathione S-transferase | 1,63 |
| *lpp2509* | PhnB-like protein | 1,62 |
| *lpp2546* | Substrate of the Dot/Icm system, SdbB effector protein | 1,53 |
| *lpp2557* | CBS-domain protein | 1,65 |
| *lpp2559* | Small heat shock protein HspC2 | 1,66 |
| *lpp2567* | Hypothetical protein | 1,85 |
| *lpp2580* | Cation/multidrug efflux pump | 1,59 |
| *lpp2581* | Efflux transporter, membrane-fusion protein MFP subunit | 1,52 |
| *lpp2629* | Hypothetical protein | 1,77 |
| *lpp2637* | Substrate of the Dot/Icm system, SidF effector protein | 1,69 |
| *lpp2658* | Uncharacterized conserved protein | 1,82 |
| *lpp2661* | UDP-3-O-acyl N-acetylglycosamine deacetylase | 1,69 |
| *lpp2663* | Cell division ATPase FtsA | 1,52 |
| *lpp2664* | Cell division septal protein FtsQ | 1,52 |
| *lpp2666* | UDP-N-acetylenolpyruvoylglucosamine reductase MurB | 1,61 |
| *lpp2667* | UDP-N-acetylmuramate:L-alanine ligase MurC | 1,60 |
| *lpp2675* | Papain-like C1 peptidase | 3,43 |
| *lpp2679* | Hypothetical protein | 1,65 |
| *lpp2692* | Enhanced entry protein EnhC, Sel1-like repeats protein | 1,94 |
| *lpp2694* | Enhanced entry protein EnhA, L,D-transpeptidase catalytic domain | 1,89 |
| *lpp2715* | 3-methyl-2-oxobutanoate hydroxymethyltransferase PanB | 1,92 |
| *lpp2716* | Pantothenate synthetase PanC | 1,56 |
| *lpp2739* | Small basic protein SbpA | 1,64 |
| *lpp2740* | Dot/Icm secretion system protein DotA | 1,66 |
| *lpp2742* | Dot/Icm secretion system protein IcmW | 1,79 |
| *lpp2786* | Cytochrome c5 | 1,53 |
| *lpp2809* | Protein of unknown function | 3,32 |
| *lpp2849* | Hypothetical protein | 1,93 |
| *lpp2851* | Dipeptide/tripeptide permease, POT family | 1,76 |
| *lpp2869* | Protein of unknown function | 2,34 |
| *lpp2894* | GDSL-like hydrolase/fatty acyltransferase | 2,17 |
| *lpp2903* | Serine-rich hypothetical protein | 1,70 |
| *lpp2909* | Amine oxidase, flavin-containing superfamily | 2,43 |
| *lpp2920* | Hypothetical protein | 1,65 |
| *lpp3021* | Protein of unknown function | 2,52 |
| *lpp3023* | Hypothetical protein | 2,45 |
| *lpp3026* | Integration host factor IHF, beta subunit | 2,08 |
| *lpp3032* | Major outer membrane protein precursor | 1,55 |
| *lpp3033* | Major outer membrane protein precursor | 1,90 |
| *lpp3047* | Substrate of the Dot/Icm secretion system MavQ | 1,54 |
| *lpp3061* | Hypothetical protein | 2,33 |

Down-regulated during exponential growth phase in the *csrA***^-^** strain (p<0.05, 44 down-regulated also in proteome analyses)

| **gene.ID** | **description** | **FC** |
| --- | --- | --- |
| *lpp0020* | Putative integral membrane protein | 0,53 |
| *lpp0030* | Hypothetical protein | 0,63 |
| *lpp0100* | Predicted membrane protein of unknown function | 0,53 |
| *lpp0123* | Uncharacterized protein | 0,54 |
| *lpp0124* | Farnesyl-diphosphate farnesyltransferase (squalene synthetase) | 0,64 |
| *lpp0148* | ProQ/FinO family protein | 0,55 |
| *lpp0149* | Hypothetical protein containing an MltA-like domain | 0,48 |
| *lpp0186* | Hypothetical protein | 0,55 |
| *lpp0187* | Hypothetical protein | 0,53 |
| *lpp0188* | Nucleoside deaminase | 0,67 |
| *lpp0190* | Hypothetical protein | 0,63 |
| *lpp0234* | Substrate of the Dot/Icm secretion system | 0,45 |
| *lpp0288* | Heme oxygenase | 0,54 |
| *lpp0332* | Putative protein conserved in bacteria | 0,31 |
| *lpp0366* | Lysine-2,3-aminomutase-like protein | 0,60 |
| *lpp0437* | Outer membrane lipoprotein SmpA | 0,55 |
| *lpp0441* | Hypothetical protein | 0,28 |
| *lpp0483* | Glucose-6-phosphate 1-dehydrogenase | 0,46 |
| *lpp0484* | 6-phosphogluconolactonase | 0,55 |
| *lpp0485* | 6-phosphogluconate dehydratase | 0,47 |
| *lpp0486* | Glucokinase | 0,57 |
| *lpp0487* | KDPG/KHG aldolase | 0,57 |
| *lpp0489* | Glycosyl hydrolase, similar to eukaryotic glucan 1-4-alpha-glucosidase | 0,60 |
| *lpp0552* | Arginine repressor ArgR | 0,66 |
| *lpp0559* | Adenosine deaminase | 0,46 |
| *lpp0569* | Outer membrane protein assembly complex, YaeT protein | 0,55 |
| *lpp0581* | Substrate of the Dot/Icm secretion system | 0,26 |
| *lpp0604* | Major facilitator superfamily (MFS) transporter PhtA | 0,62 |
| *lpp0628* | Tyrosyl-tRNA synthetase | 0,66 |
| *lpp0644* | Hypothetical protein | 0,59 |
| *lpp0645* | Aminodeoxychorismate lyase PabC | 0,51 |
| *lpp0670* | 7-cyano-7-deazaguanine reductase QueF | 0,60 |
| *lpp0671* | Major outer membrane protein | 0,40 |
| *lpp0695* | ATP-dependent protease, ATP-binding subunit HslU | 0,55 |
| *lpp0700* | WrbA-like trp repressor binding protein | 0,67 |
| *lpp0706* | Major facilitator superfamily (MFS) transporter PhtE | 0,58 |
| *lpp0733* | Hypothetical protein | 0,51 |
| *lpp0740* | Radical SAM methylthiotransferase, miab/rimo family | 0,63 |
| *lpp0781* | Transcriptional regulatory protein | 0,64 |
| *lpp0788* | Hypothetical protein | 0,33 |
| *lpp0789* | Hypothetical protein | 0,22 |
| *lpp0830* | Tetratricopeptide repeat (TPR)-domain protein | 0,58 |
| *lpp0838* | O-antigenic polysaccharide transporter protein Wzt, kpst/Wzt ABC transporter | 0,65 |
| *lpp0839* | Hypothetical protein | 0,67 |
| *lpp0844* | Zn-dependent hydrolases, putative glyoxalase II family | 0,48 |
| *lpp0852* | Protein containing GDSL-like lipase/acylhydrolase domains | 0,61 |
| *lpp0861* | Quinolinate synthetase NadA | 0,63 |
| *lpp0917* | Signal peptide peptidase A SppA | 0,54 |
| *lpp0936* | Hypothetical protein | 0,59 |
| *lpp0940* | Uncharacterized protein with SCP domain | 0,46 |
| *lpp0944* | Hypothetical protein, predicted transmembrane protein | 0,50 |
| *lpp0959* | Substrate of the Dot/Icm secretion system | 0,46 |
| *lpp0961* | AsmA-like protein | 0,57 |
| *lpp0971* | Cytochrome c-type protein | 0,64 |
| *lpp0986* | alanine dehydrogenase | 0,63 |
| *lpp1042* | Hypothetical protein | 0,59 |
| *lpp1083* | Polypeptide deformylase | 0,50 |
| *lpp1084* | Hypothetical protein | 0,55 |
| *lpp1120* | Histidine acid phosphatase | 0,50 |
| *lpp1122* | Hypothetical protein | 0,31 |
| *lpp1138* | Hypothetical protein | 0,23 |
| *lpp1154* | Hypothetical protein | 0,60 |
| *lpp1155* | (Poly)amine oxidoreductase | 0,50 |
| *lpp1167* | Uridine monophosphate kinase | 0,53 |
| *lpp1173* | Substrate of the Dot/Icm secretion system | 0,64 |
| *lpp1180* | Riboflavin biosynthesis protein RibD | 0,54 |
| *lpp1181* | Riboflavin synthase, alpha subunit | 0,62 |
| *lpp1182* | Riboflavin biosynthesis protein RibA | 0,59 |
| *lpp1183* | Riboflavin synthase, beta subunit | 0,66 |
| *lpp1188* | Membrane assembly lipoprotein YfiO | 0,54 |
| *lpp1192* | AdoMet-dependent methyltransferase | 0,65 |
| *lpp1223* | Coproporphyrinogen III oxidase | 0,64 |
| *lpp1257* | Membrane bound lytic murein transglycosylase D | 0,64 |
| *lpp1303* | Apolipoprotein N-acyltransferase | 0,66 |
| *lpp1327* | Ribonuclease HII | 0,63 |
| *lpp1332* | Iojap-like ribosome-associated protein | 0,56 |
| *lpp1363* | Choline kinase ChoK | 0,64 |
| *lpp1376* | 30S ribosomal protein S1 | 0,57 |
| *lpp1388* | 2-deoxyribose-5-phosphate aldolase | 0,46 |
| *lpp1389* | Purine nucleoside phosphorylase | 0,36 |
| *lpp1390* | Cytidine deaminase | 0,41 |
| *lpp1402* | Pseudouridylate synthase | 0,66 |
| *lpp1409* | Hypothetical protein | 0,54 |
| *lpp1438* | Protein of unknown function | 0,51 |
| *lpp1445* | Hypothetical protein | 0,48 |
| *lpp1451* | Gamma-glutamyl cyclotransferase GGCT-like protein | 0,64 |
| *lpp1452* | Hypothetical protein | 0,58 |
| *lpp1454* | Aminopeptidase N | 0,64 |
| *lpp1476* | Hypoxanthine-guanine phosphoribosyltransferase | 0,65 |
| *lpp1491* | Glutamate-1-semialdehyde aminotransferase | 0,61 |
| *lpp1492* | Rubredoxin | 0,57 |
| *lpp1515* | Pyruvate dehydrogenase E1 component, alpha subunit | 0,57 |
| *lpp1516* | Pyruvate dehydrogenase E1 component, beta subunit | 0,62 |
| *lpp1543* | Conserved protein, similar to enhanced entry protein EnhB | 0,63 |
| *lpp1546* | Substrate of the Dot/Icm secretion system | 0,49 |
| *lpp1547* | 50S ribosomal protein L9 | 0,55 |
| *lpp1620* | Inosose dehydratase IolE | 0,61 |
| *lpp1621* | 3D-(3,5/4)-trihydroxycyclohexane-1,2-dione hydrolase IolD | 0,63 |
| *lpp1622* | 5-dehydro-2-deoxygluconokinase/5-deoxy-glucuronate isomerase IolCB | 0,65 |
| *lpp1628* | N-dimethylarginine dimethylaminohydrolase | 0,67 |
| *lpp1634* | Hypothetical protein | 0,43 |
| *lpp1667* | Substrate of the Dot/Icm secretion system | 0,66 |
| *lpp1669* | 30S ribosomal protein S2 | 0,65 |
| *lpp1681* | Hypothetical protein | 0,63 |
| *lpp1707* | Global DNA-binding transcriptional regulator Fis3 | 0,35 |
| *lpp1740* | Hypothetical protein | 0,29 |
| *lpp1766* | Substrate of the Dot/Icm secretion system | 0,35 |
| *lpp1768* | DNA mismatch repair protein MutS | 0,66 |
| *lpp1771* | Porphobilinogen synthase | 0,62 |
| *lpp1819* | Type I phosphodiesterase/nucleotide pyrophosphatase | 0,43 |
| *lpp1860* | Major facilitator superfamily (MFS) transporter PhtJ | 0,60 |
| *lpp1891* | BolA-like protein | 0,48 |
| *lpp1899* | Hypothetical protein | 0,52 |
| *lpp1927* | Transcriptional regulator, luxr family | 0,60 |
| *lpp1935* | Hypothetical protein, putative coiled-coil domain | 0,62 |
| *lpp1948* | Protein of unknown function | 0,22 |
| *lpp1981* | Preprotein translocase, subunit SecF | 0,59 |
| *lpp1982* | Preprotein translocase, subunit SecD | 0,57 |
| *lpp1983* | Preprotein translocase, subunit YajC | 0,60 |
| *lpp1992* | Guanosine monophosphate kinase | 0,57 |
| *lpp1993* | YicC-like protein | 0,65 |
| *lpp2000* | Hypothetical protein | 0,58 |
| *lpp2097* | AdoMet-dependent methyltransferase | 0,64 |
| *lpp2101* | Hypothetical protein | 0,65 |
| *lpp2128* | Eukaryotic-like sphingosine-1-phosphate lyase 1 | 0,29 |
| *lpp2164* | Heme-binding protein Hbp | 0,24 |
| *lpp2225* | Substrate of the Dot/Icm secretion system | 0,62 |
| *lpp2230* | Amino acid (Glu/Leu/Phe/Val) dehydrogenase | 0,60 |
| *lpp2236* | Asparaginyl-tRNA synthetase | 0,57 |
| *lpp2247* | ATP-dependent DNA/RNA helicase HepA | 0,63 |
| *lpp2269* | Hydroxy/aromatic amino acid permease (HAAAP), ser/thr subfamily | 0,38 |
| *lpp2275* | Substrate of the Dot/Icm secretion system | 0,35 |
| *lpp2278* | Trans-isoprenyl diphosphate synthase | 0,48 |
| *lpp2294* | ATP-dependent RNA helicase DeaD | 0,63 |
| *lpp2308* | Yqey-like protein, core Dot/Icm substrate | 0,55 |
| *lpp2309* | DNA primase DnaG | 0,66 |
| *lpp2313* | N-acyltransferase | 0,37 |
| *lpp2314* | Metabolite-proton symporter | 0,57 |
| *lpp2315* | N-acetyltransferase, GNAT family | 0,32 |
| *lpp2316* | Alpha/beta hydrolase | 0,34 |
| *lpp2400* | Hypothetical protein | 0,45 |
| *lpp2430* | Hypothetical protein | 0,20 |
| *lpp2431* | Cupin domain protein | 0,27 |
| *lpp2432* | Protein of unknown function, ATP-grasp domain | 0,15 |
| *lpp2433* | H+ antiporter protein, putative | 0,11 |
| *lpp2434* | Hypothetical protein | 0,13 |
| *lpp2435* | Flavin reductase-like protein | 0,12 |
| *lpp2436* | Hypothetical protein | 0,57 |
| *lpp2437* | Tetrapyrrole (corrin/porphyrin) methylase | 0,61 |
| *lpp2438* | Hypothetical protein | 0,59 |
| *lpp2439* | Major facilitator superfamily (MFS) protein | 0,48 |
| *lpp2458* | Substrate of the Dot/Icm system, SdbC effector protein | 0,57 |
| *lpp2476* | Domain of unknown function (DUF4116) | 0,61 |
| *lpp2480* | Substrate of the Dot/Icm secretion system | 0,50 |
| *lpp2483* | Eukaryotic RasGap-like domain | 0,52 |
| *lpp2486* | Eukaryotic-like protein, F-box domain | 0,45 |
| *lpp2504* | Hypothetical protein | 0,51 |
| *lpp2519* | Hypothetical protein | 0,59 |
| *lpp2521* | Protein of unknown function | 0,45 |
| *lpp2570* | Multidrug resistance transporter, MFS superfamily | 0,63 |
| *lpp2586* | Hypothetical membrane protein | 0,64 |
| *lpp2587* | Protein of unknown function | 0,34 |
| *lpp2590* | Low specificity D-threonine aldolase | 0,67 |
| *lpp2591* | Substrate of the Dot/Icm secretion system MavL | 0,61 |
| *lpp2594* | Substrate of the Dot/Icm secretion system | 0,40 |
| *lpp2612* | Hypothetical protein | 0,43 |
| *lpp2622* | Hypothetical membrane protein | 0,38 |
| *lpp2623* | Sugar phosphate permease/sensor protein UhpC | 0,44 |
| *lpp2690* | Substrate of the Dot/Icm secretion system | 0,29 |
| *lpp2698* | Excinuclease ABC subunit C | 0,63 |
| *lpp2707* | Gtpase ychf, Obg family | 0,61 |
| *lpp2726* | Peptidase M16 | 0,65 |
| *lpp2783* | Peptidyl-prolyl cis-trans isomerase, cyclophilin family | 0,46 |
| *lpp2784* | Queuine tRNA-ribosyltransferase | 0,66 |
| *lpp2838* | Triosephosphate isomerase | 0,48 |
| *lpp2839* | LepA effector protein | 0,61 |
| *lpp2943* | Hypothetical protein | 0,34 |
| *lpp2986* | N-acyltransferase | 0,60 |
| *lpp2987* | Hypothetical protein | 0,36 |
| *lpp3012* | Bacterial lipid A biosynthesis acyltransferase | 0,45 |
| *lpp3013* | Bacterial lipid A biosynthesis acyltransferase | 0,56 |
| *lpp3014* | Lipid A-disaccharide synthase LpxB | 0,59 |
| *lpp3015* | UDP-3-O-[3-hydroxymyristoyl] glucosamine N-acyltransferase LpxD | 0,61 |
| *lpp3016* | UDP-N-acetylglucosamine acetyltransferase LpxA | 0,56 |
| *lpp3020* | Glycosyltransferase, GT1 family | 0,61 |
| *lpp3027* | Deoxycytidine triphosphate deaminase | 0,54 |
| *lpp3030* | Protein of unknown function (DUF3450) | 0,20 |
| *lpp3072* | Membrane protein of unknown function | 0,42 |
| *lpp3073* | tRNA modification GTPase TrmE | 0,62 |
| *sRNA* | Lppnc0121 | 0,39 |
| *sRNA* | Lppnc0248 | 0,40 |
| *sRNA* | Lppnc0278 | 0,52 |
| *sRNA* | RsmY | 0,06 |
| *sRNA* | RsmX | 0,13 |
| *sRNA* | RsmZ | 0,19 |

B) Up-regulated during post-exponential phase in the *csrA*^-^ strain (p<0.05)

| **gene.ID** | **description** | **FC** |
| --- | --- | --- |
| *lpp0733* | Hypothetical protein | 2,11 |
| *lpp0845* | Global regulator CsrA | 3,37 |
| *lpp1454* | Aminopeptidase N | 2,01 |
|  |  |  |

Down-regulated during post- exponential phase in the *csrA*^-^ strain (p<0.05)

| **gene.ID** | **description** | **FC** |
| --- | --- | --- |
| *sRNA* | RsmX | 0,11 |
| *sRNA* | RsmY | 0,13 |
| *sRNA* | RsmZ | 0,16 |
|  |  |  |
