# Supplementary material for: The Legionella pneumophila genome evolved to accommodate multiple regulatory mechanisms controlled by the CsrA-system
Source: PLoS Genet. 2017 Feb 17;13(2):e1006629. doi: 10.1371/journal.pgen.1006629 (PMC5338858; doi:10.1371/journal.pgen.1006629)
Supplement: S2 Table — (DOCX) [file pgen.1006629.s015.docx]

**Table S2: Proteins identified as differentially expressed by MS analyses (FDR < 0.05)**

Up-regulated in the *csrA^-^* strain (FDR < 0.05, 65 up-regulated also in the transcriptome analyses)

| **Protein IDs** | **Protein name** | **FC** |
| --- | --- | --- |
| Lpp0008 | RavA, substrate of the Dot/Icm secretion system | 1,57 |
| Lpp0014 | Patatin-like phospholipase, esterase of the alpha-beta hydrolase superfamily | 2,51 |
| Lpp0055 | Protein of unknown function | 1,81 |
| Lpp0056 | Protein of unknown function | 2,16 |
| Lpp0122 | Protein of unknown function | 3,81 |
| Lpp0145 | Similarity with eukaryotic proteins, substrate of the Dot/Icm secretion system | 1,57 |
| Lpp0165 | Peptidase S24 LexA-like | 1,78 |
| Lpp0177 | Legionella *vir*-like protein LvhB9 | 2,77 |
| Lpp0188 | Nucleoside deaminase | 1,97 |
| Lpp0247 | NAD/FAD-binding protein, putative amine oxidase | 2,93 |
| Lpp0315 | NAD-glutamate dehydrogenase | 1,63 |
| Lpp0358 | AdoMet-dependent methyltransferase | 1,69 |
| Lpp0359 | NAD-dependent formate dehydrogenase | 6,48 |
| Lpp0488 | Sugar transporter, MFS superfamily | 2,89 |
| Lpp0493 | Cold shock-like protein CspD | 17,33 |
| Lpp0546 | Peptidase M42 family protein | 2,61 |
| Lpp0547 | AnkC, substrate of the Dot/Icm secretion system | 1,86 |
| Lpp0560 | ABC-type amino acid transporter, bacterial periplasmic component | 1,73 |
| Lpp0608 | LolB, outer membrane lipoprotein | 1,62 |
| Lpp0620 | Acetoacetyl-CoA reductase | 3,52 |
| Lpp0621 | Acetoacetyl-CoA reductase | 2,76 |
| Lpp0622 | PhaR ,Polyhydroxyalkanoate synthesis regulator | 2,72 |
| Lpp0640 | Mg^2+^ chelatase-related protein, predicted ATPase with chaperone activity | 2,94 |
| Lpp0663 | Zn-dependent alcohol dehydrogenase | 2,69 |
| Lpp0679 | StaR-like protein, TPR-domain - eukaryotic-like protein | 2,04 |
| Lpp0725 | Predicted integral membrane protein (DUF2282) | 4,14 |
| Lpp0727 | NADH dehydrogenase, FAD-containing subunit | 2,74 |
| Lpp0728 | Acetoacetate decarboxylase | 2,60 |
| Lpp0799 | RavH, substrate of the Dot/Icm secretion system | 4,40 |
| Lpp0809 | Signal transduction protein, GGDEF domain | 1,78 |
| Lpp0841 | O-acetyltransferase Lag-1 | 2,27 |
| Lpp0865 | Acyl-CoA dehydrogenase | 6,49 |
| Lpp0872 | ComEA , competence protein | 7,26 |
| Lpp0915 | FleQ , transcriptional regulator | 2,66 |
| Lpp0931 | Acyl-CoA dehydrogenase | 1,73 |
| Lpp0937 | PntB, NAD(P) transhydrogenase beta subunit | 1,63 |
| Lpp0939 | PntA, NAD(P) transhydrogenase alpha subunit | 2,60 |
| Lpp0962 | Protein of unknown function | 9,43 |
| Lpp0963 | Protein of unknown function | 6,22 |
| Lpp0964 | Protein of unknown function | 6,07 |
| Lpp0970 | FlgA, flagellar basal body P-ring biosynthesis protein | 1,73 |
| Lpp0982 | MavT, substrate of the Dot/Icm secretion system | 2,68 |
| Lpp0991 | PilO, Tfp pilus assembly protein | 1,77 |
| Lpp0997 | UspA, universal stress protein | 3,01 |
| Lpp1025 | Substrate of the Dot/Icm secretion system | 3,57 |
| Lpp1030 | SidK, substrate of the Dot/Icm secretion system | 1,62 |
| Lpp1085 | Helix-turn-helix, XRE-family like protein | 3,11 |
| Lpp1113 | Protein of unknown function | 2,92 |
| Lpp1157 | Eukaryotic-like pyruvate decarboxylase | 1,62 |
| Lpp1177 | Transcription regulator, AsnC family | 5,38 |
| Lpp1226 | FlgD, flagellar basal-body rod modification protein | 3,13 |
| Lpp1227 | FlgE, flagellar hook protein | 6,53 |
| Lpp1229 | FlgG, flagellar biosynthesis protein | 2,33 |
| Lpp1236 | Substrate of the Dot/Icm secretion system | 1,89 |
| Lpp1237 | Protein of unknown function | 3,13 |
| Lpp1291 | FliS, flagellar protein | 5,11 |
| Lpp1292 | FliD, flagellar hook-associated protein | 3,15 |
| Lpp1294 | FlaA, flagellin | 14,17 |
| Lpp1310 | Sel1 repeat protein, substrate of the Dot/Icm secretion system | 1,72 |
| Lpp1324 | Fis2, global DNA-binding transcriptional regulator | 4,52 |
| Lpp1336 | NAD-dependent aldehyde dehydrogenase | 1,62 |
| Lpp1340 | Protein of unknown function | 4,46 |
| Lpp1369 | GlpK, glycerol kinase | 1,59 |
| Lpp1618 | YceI-like protein | 2,50 |
| Lpp1619 | Predicted integral membrane protein (DUF2282) | 1,96 |
| Lpp1723 | FliG, flagellar motor switch protein | 1,60 |
| Lpp1747 | FleN, anti-activator of flagellar biosynthesis | 2,72 |
| Lpp1748 | FlhF, flagellar biosynthesis regulator | 2,63 |
| Lpp1756 | FliM, flagellar motor switch protein | 2,63 |
| Lpp1800 | Protein of unknown function | 5,57 |
| Lpp1826 | DNA-binding protein HU-beta | 2,84 |
| Lpp1835 | Transmembrane protein | 1,75 |
| Lpp1846 | Glyoxylase I | 2,14 |
| Lpp1848 | YlfB, substrate of the Dot/Icm secretion system | 2,09 |
| Lpp1856 | Alpha/beta hydrolase | 2,32 |
| Lpp1883 | Glutathionine S-transferase | 2,90 |
| Lpp1963 | Eukaryotic-like tetratricopeptide repeat (TPR)-domain protein | 1,67 |
| Lpp1995 | PilT, Tfp pilus assembly protein, pilus retraction ATPase | 1,86 |
| Lpp2015 | RecG, ATP-dependent DNA helicase | 4,47 |
| Lpp2039 | Protein of unknown function | 2,68 |
| Lpp2140 | IcmL-like protein | 2,81 |
| Lpp2146 | Ketosteroid isomerase, NTF2-like superfamily | 1,94 |
| Lpp2163 | Arginase/histone deacetylase-like superfamily | 3,70 |
| Lpp2177 | Acyl-CoA dehydrogenase | 3,78 |
| Lpp2194 | GTP cyclohydrolase II | 1,77 |
| Lpp2195 | Protein of unknown function (DUF1688) | 1,70 |
| Lpp2206 | Glutamine synthetase | 2,06 |
| Lpp2209 | Membrane protein of unkown function | 2,17 |
| Lpp2218 | CbpM, chaperone modulator protein | 2,53 |
| Lpp2264 | 3-hydroxybutyrate dehydrogenase | 3,16 |
| Lpp2266 | MotA, flagellar motor protein | 1,92 |
| Lpp2272 | Eukaryotic-like sugar 1,4-lactone oxidase domain | 8,57 |
| Lpp2276 | Substrate of the Dot/Icm secretion system | 2,09 |
| Lpp2304 | Aspartyl/glutamyl-tRNA (Asn/Gln) amidotransferase and related amidases | 2,43 |
| Lpp2320 | Protein of unknown function | 3,97 |
| Lpp2321 | Cold shock protein | 5,80 |
| Lpp2322 | Acetoacetyl-CoA reductase | 11,67 |
| Lpp2358 | Phosphoribosylpyrophosphate synthetase | 2,14 |
| Lpp2359 | Thymidine/pyrimidine nucleoside phosphorylase | 3,01 |
| Lpp2360 | Metal-dependent ribonuclease, beta-lactamase fold | 1,93 |
| Lpp2362 | Chemiosmotic efflux system B protein B | 3,05 |
| Lpp2368 | Domain of unknown function (DUF302) | 2,82 |
| Lpp2370 | Cation transport ATPase | 1,65 |
| Lpp2375 | Cupin domain protein | 2,95 |
| Lpp2435 | Flavin reductase-like protein | 2,45 |
| Lpp2461 | Protein of unknown function | 3,21 |
| Lpp2491 | MavH, substrate of the Dot/Icm secretion system | 2,05 |
| Lpp2501 | Cupin 2 superfamily protein, substrate of the Dot/Icm secretion system | 2,82 |
| Lpp2502 | Protein of unknown function (DUF1311) | 2,28 |
| Lpp2506 | NADPH-dependent FMN reductase | 2,20 |
| Lpp2553 | RadC-like DNA repair protein | 21,07 |
| Lpp2555 | LepB, substrate of the Dot/Icm secretion system | 4,84 |
| Lpp2559 | HspC2, small heat shock protein | 3,37 |
| Lpp2569 | Carbonic anhydrase | 2,29 |
| Lpp2659 | Glutamine amidotransferase | 1,81 |
| Lpp2675 | Papain-like C1 peptidase | 2,67 |
| Lpp2715 | PanB, 3-methyl-2-oxobutanoate hydroxymethyltransferase | 1,63 |
| Lpp2732 | AdoMet-dependent methyltransferase | 4,36 |
| Lpp2739 | SbpA, small basic protein | 1,63 |
| Lpp2764 | IHF, Integration host factor, alpha subunit | 2,09 |
| Lpp2773 | Helix-turn-helix protein, XRE family-like | 2,16 |
| Lpp2809 | Protein of unknown function | 2,46 |
| Lpp2865 | VipE substrate of the Dot/Icm secretion system | 1,76 |
| Lpp2869 | Protein of unknown function | 1,70 |
| Lpp2894 | GDSL-like hydrolase/fatty acyltransferase | 1,74 |
| Lpp2896 | Excinuclease ABC C subunit domain protein | 5,99 |
| Lpp2909 | Amine oxidase, flavin-containing superfamily | 2,63 |
| Lpp3021 | Protein of unknown function | 3,10 |
| Lpp3026 | IHF, Integration host factor, beta subunit | 2,05 |
| Lpp3043 | NADP-dependent malic enzyme | 2,00 |
| Lpp3047 | MavQ, substrate of the Dot/Icm secretion system | 1,67 |

Down-regulated in the *csrA*^-^ strain (FDR < 0.05, 44 down-regulated also in the transcriptome analyses)

| **Protein IDs** | **Protein name** | **FC** |
| --- | --- | --- |
| Lpp0022 | 23S rRNA m(2)G2445 methyltransferase | 0,49 |
| Lpp0108 | Ribose-5-phosphate isomerase A | 0,62 |
| Lpp0231 | Mce (mammalian cell entry)-related protein | 0,27 |
| Lpp0234 | Substrate of the Dot/Icm secretion system | 0,61 |
| Lpp0280 | Protein of unknown function (DUF2309) | 0,31 |
| Lpp0304 | SidE, substrate of the Dot/Icm secretion system | 0,36 |
| Lpp0332 | Substrate of the Dot/Icm secretion system | 0,36 |
| Lpp0366 | Lysine-2,3-aminomutase-like protein | 0,57 |
| Lpp0559 | Adenosine deaminase | 0,44 |
| Lpp0597 | SucA, 2-oxoglutarate dehydrogenase, E1 subunit | 0,65 |
| Lpp0670 | QueF, 7-cyano-7-deazaguanine reductase | 0,36 |
| Lpp0706 | PhtE, major facilitator superfamily (MFS) transporter | 0,40 |
| Lpp0720 | Lytic murein transglycosylase | 0,56 |
| Lpp0793 | NusB, transcription termination factor | 0,61 |
| Lpp0844 | Zn-dependent hydrolases, putative glyoxalase II family | 0,58 |
| Lpp0845 | CsrA, Global regulator | 0,56 |
| Lpp0908 | BolA-like protein | 0,32 |
| Lpp0928 | Putative Lipoprotein, domain of unknown function (DUF4156), | 0,53 |
| Lpp0940 | Uncharacterized protein with SCP domain | 0,48 |
| Lpp0950 | N-acyltransferase superfamily | 0,59 |
| Lpp0959 | Substrate of the Dot/Icm secretion system | 0,33 |
| Lpp0961 | AsmA-like protein | 0,53 |
| Lpp1018 | Protein of unknown function | 0,59 |
| Lpp1132 | Protein of unknown function | 0,46 |
| Lpp1173 | Substrate of the Dot/Icm secretion system | 0,15 |
| Lpp1181 | Riboflavin synthase, alpha subunit | 0,61 |
| Lpp1182 | RibA, Riboflavin biosynthesis protein | 0,62 |
| Lpp1192 | AdoMet-dependent methyltransferase | 0,56 |
| Lpp1356 | PilZ, Tfp pilus assembly protein | 0,60 |
| Lpp1363 | LicA, putative choline kinase, substrate of the Dot/Icm secretion system | 0,32 |
| Lpp1387 | FAD/FMN-containing dehydrogenase | 0,12 |
| Lpp1388 | 2-deoxyribose-5-phosphate aldolase | 0,57 |
| Lpp1389 | Purine nucleoside phosphorylase | 0,57 |
| Lpp1397 | Metal-dependent phosphoesterase, PHP family | 0,52 |
| Lpp1402 | Pseudouridylate synthase | 0,64 |
| Lpp1412 | RumA, 23S rRNA (Uracil-5)-methyltransferase | 0,18 |
| Lpp1413 | RelA, GTP pyrophosphokinase | 0,62 |
| Lpp1438 | Protein of unknown function | 0,57 |
| Lpp1454 | Aminopeptidase N | 0,58 |
| Lpp1461 | Pyruvate dehydrogenase complex, decarboxylase component E1 | 0,65 |
| Lpp1481 | Protein of unknown function (DUF1820) | 0,32 |
| Lpp1546 | Substrate of the Dot/Icm secretion system | 0,30 |
| Lpp1574 | ProA, Gamma-glutamyl phosphate reductase | 0,45 |
| Lpp1658 | Substrate of the Dot/Icm secretion system | 0,55 |
| Lpp1665 | Uracil-DNA glycosylase | 0,62 |
| Lpp1680 | CpxP-like protein, envelope stress induced periplasmic protein | 0,50 |
| Lpp1692 | Alpha/beta hydrolase superfamily protein | 0,39 |
| Lpp1707 | Global DNA-binding transcriptional regulator Fis3 | 0,47 |
| Lpp1715 | Substrate of the Dot/Icm secretion system | 0,54 |
| Lpp1763 | Alanyl-tRNA synthetase | 0,51 |
| Lpp1766 | Substrate of the Dot/Icm secretion system | 0,44 |
| Lpp1771 | HemB, Porphobilinogen synthase | 0,57 |
| Lpp1819 | Type I phosphodiesterase/nucleotide pyrophosphatase | 0,60 |
| Lpp1948 | Protein of unknown function | 0,17 |
| Lpp1950 | Protein of unknown function | 0,48 |
| Lpp1955 | PieF, Substrate of the Dot/Icm secretion system | 0,62 |
| Lpp1994 | Ribonuclease PH | 0,64 |
| Lpp2093 | SdeD, substrate of the Dot/Icm secretion system | 0,56 |
| Lpp2094 | SidJ, substrate of the Dot/Icm secretion system | 0,51 |
| Lpp2128 | Spl, sphingosine-1-phosphate lyase 1, substrate of the Dot/Icm secretion system | 0,45 |
| Lpp2202 | Substrate of the Dot/Icm secretion system | 0,63 |
| Lpp2230 | Amino acid (Glu/Leu/Phe/Val) dehydrogenase | 0,52 |
| Lpp2247 | HepA, ATP-dependent DNA/RNA helicase | 0,52 |
| Lpp2269 | Hydroxy/aromatic amino acid permease (HAAAP), serine/threonine subfamily | 0,20 |
| Lpp2275 | Substrate of the Dot/Icm secretion system | 0,27 |
| Lpp2432 | Protein of unknown function, ATP-grasp domain | 0,53 |
| Lpp2433 | H+ antiporter protein, putative | 0,39 |
| Lpp2458 | SdbC, substrate of the Dot/Icm system | 0,32 |
| Lpp2480 | Substrate of the Dot/Icm secretion system | 0,50 |
| Lpp2483 | Eukaryotic Ras Gap-like domain | 0,58 |
| Lpp2513 | Protein of unknown function | 0,20 |
| Lpp2521 | Protein of unknown function | 0,35 |
| Lpp2583 | Uncharacterized protein conserved in bacteria | 0,60 |
| Lpp2587 | Protein of unknown function | 0,21 |
| Lpp2591 | MavL, substrate of the Dot/Icm secretion system | 0,50 |
| Lpp2594 | Substrate of the Dot/Icm secretion system | 0,33 |
| Lpp2674 | Acid phosphatase, HAD superfamily | 0,47 |
| Lpp2690 | Substrate of the Dot/Icm secretion system | 0,20 |
| Lpp2783 | Peptidyl-prolyl cis-trans isomerase, cyclophilin family | 0,39 |
| Lpp2839 | LepA, substrate of the Dot/Icm secretion system | 0,32 |
| Lpp2931 | RNA pyrophosphohydrolase | 0,53 |
| Lpp2938 | Substrate of the Dot/Icm secretion system | 0,52 |
| Lpp3004 | Substrate of the Dot/Icm secretion system, RNA methyltransferase RsmE family | 0,56 |
| Lpp3030 | Protein of unknown function (DUF3450) | 0,17 |
| Lpp4274 | Protein of unknown function | 0,15 |
