# Supplementary material for: The Legionella pneumophila genome evolved to accommodate multiple regulatory mechanisms controlled by the CsrA-system
Source: PLoS Genet. 2017 Feb 17;13(2):e1006629. doi: 10.1371/journal.pgen.1006629 (PMC5338858; doi:10.1371/journal.pgen.1006629)
Supplement: S3 Table — (DOCX) [file pgen.1006629.s016.docx]

**Table S3:** RNA targets identified using Co-immunoprecipitation with anti-CsrA antibodies followed by deep sequencing (RIPseq) analyses

| Target | **Carboydrate metabolism, Energy (38 targets identified)** | FC | N°of IP |
| --- | --- | --- | --- |
| Lpp0054 | Protein of the ICL/PEPM enzyme family, 2-methylisocitrate lyase | 8.02 | 2 |
| Lpp0108 | Ribose-5-phosphate isomerase A | 18.09 | 2 |
| Lpp0151 | PykA, pyruvate kinase II - glucose stimulated | 21.08 | 3 |
| Lpp0152 | 3-phosphoglycerate kinase | 39.79 | 4 |
| Lpp0153 | Glyceraldehyde 3-phosphate dehydrogenase | 37.41 | 3 |
| Lpp0294 | CyoA, cytochrome o ubiquinol oxidase subunit II | 22.44 | 3 |
| Lpp0359 | NAD-dependent formate dehydrogenase | 14.50 | 3 |
| Lpp0483 | Glucose-6-phosphate 1-dehydrogenase | 12.91 | 3 |
| Lpp0484 | 6-phosphogluconolactonase | 26.21 | 2 |
| Lpp0485 | 6-phosphogluconate dehydratase | 23.41 | 5 |
| Lpp0531 | Pyruvate carboxylase subunit B | 26.95 | 2 |
| Lpp0535 | Fructose-bisphosphate aldolase | 42.92 | 3 |
| Lpp0593 | SdhC subunit, Succinate dehydrogenase, | 5.10 | 1 |
| Lpp0595 | SdhA subunit, succinate dehydrogenase, flavoprotein | 19.55 | 3 |
| Lpp0597 | 2-oxoglutarate dehydrogenase, E1 subunit SucA | 38.36 | 5 |
| Lpp0607 | PrsA, ribose-phosphate pyrophosphokinase | 21.87 | 3 |
| Lpp0663 | Zn-dependent alcohol dehydrogenase | 6.02 | 2 |
| Lpp0705 | Malic enzymes (malate oxidoreductase) | 42.45 | 4 |
| Lpp0727 | NADH dehydrogenase, FAD-containing subunit | 20.27 | 3 |
| Lpp0867 | Phosphoenolpyruvate synthase | 154.93 | 2 |
| Lpp0878 | isocitrate dehydrogenase, NADP-dependent | 19.05 | 4 |
| Lpp0985 | Electron transfer flavoprotein alpha subunit | 33.22 | 3 |
| Lpp1009 | 2-oxoglutarate ferredoxin oxidoreductase alpha subunit | 13.23 | 3 |
| Lpp1459 | Pyruvate dehydrogenase complex, component E3 | 46.58 | 3 |
| Lpp1460 | Pyruvate dehydrogenase complex, component E2 | 37.08 | 3 |
| Lpp1461 | Pyruvate dehydrogenase complex, component E1 | 46.10 | 3 |
| Lpp1516 | Pyruvate/2-oxoglutarate dehydrogenase complex, component E1 | 18.93 | 3 |
| Lpp1517 | Pyruvate/2-oxoglutarate dehydrogenase complex, component E2 | 27.95 | 3 |
| Lpp1659 | Aconitate hydratase | 23.05 | 3 |
| Lpp2020 | Enolase | 12.31 | 3 |
| Lpp2204 | Iron-containing alcohol dehydrogenase | 19.15 | 4 |
| Lpp2301 | Malate dehydrogenase | 17.13 | 4 |
| Lpp2328 | F0F1-type ATP synthase, beta subunit | 11.48 | 3 |
| Lpp2569 | Carbonic anhydrase | 58.78 | 2 |
| Lpp2838 | Triosephosphate isomerase | 9.46 | 2 |
| Lpp2960 | CtaG, cytochrome c oxidase assembly protein | 11.67 | 3 |
| Lpp3053 | F0F1-type ATP synthase, beta chain | 54.95 | 2 |
| Lpp3055 | F0F1-type ATP synthase, alpha chain | 83.32 | 4 |
|  |  |  |  |
| **Aminoacid metabolism, other aminoacids (28 targets identified)** | |  |  |
| Lpp0085 | Aspartate aminotransferase | 28.84 | 2 |
| Lpp0128 | Glycine dehydrogenase subunit 2 (glycine cleavage system protein P) | 52.38 | 5 |
| Lpp0130 | Glycine dehydrogenase subunit 1 (glycine cleavage system protein P) | 47.05 | 4 |
| Lpp0131 | Glycine cleavage system protein H | 58.22 | 3 |
| Lpp0610 | Gamma-glutamyltranspeptidase | 13.18 | 3 |
| Lpp0756 | 2-amino-3-ketobutyrate coenzyme A ligase | 20.68 | 3 |
| Lpp0764 | HutG, formiminoglutamase | 15.50 | 2 |
| Lpp0791 | Glycine hydroxymethyltransferase | 123.84 | 3 |
| Lpp0815 | HisF, imidazole glycerol phosphate synthase subunit | 30.65 | 3 |
| Lpp0854 | L-serine dehydratase, iron-sulfur-dependent | 23.57 | 3 |
| Lpp0871 | M20 family dipeptidase, putative ArgE/DapE | 28.17 | 3 |
| Lpp0933 | 3-hydroxyisobutyryl-CoA hydrolase | 31.93 | 3 |
| Lpp0986 | Alanine dehydrogenase | 49.89 | 4 |
| Lpp0994 | AroK, shikimate kinase | 14.57 | 1 |
| Lpp1268 | Tryptophan synthase beta subunit | 21.05 | 4 |
| Lpp1304 | Saccharopine dehydrogenase and related proteins | 26.24 | 4 |
| Lpp1318 | Glutamine synthetase | 24.51 | 3 |
| Lpp1334 | HutU, urocanate hydratase | 23.44 | 4 |
| Lpp1415 | Aspartate aminotransferase | 22.41 | 2 |
| Lpp1539 | NAD-glutamate dehydrogenase | 23.57 | 3 |
| Lpp1670 | Peptidase M20 family, glutamate carboxypeptidase | 18.02 | 2 |
| Lpp1672 | Succinylglutamic semialdehyde dehydrogenase AstD | 28.08 | 2 |
| Lpp1787 | Isovaleryl-CoA dehydrogenase | 28.96 | 2 |
| Lpp2206 | Glutamine synthetase | 43.40 | 3 |
| Lpp2230 | Amino acid (Glu/Leu/Phe/Val) dehydrogenase | 45.68 | 3 |
| Lpp2232 | 4-hydroxyphenylpyruvate dioxygenase (legiolysin) | 17.92 | 3 |
| Lpp2233 | Fumarylacetoacetate hydrolase | 19.73 | 4 |
| Lpp3040 | ArgD, acetylornithine aminotransferase | 65.96 | 4 |
|  |  |  |  |
| **Nucleotide metabolism (17 targets identified)** | |  |  |
| Lpp0277 | PurE, phosphoribosylaminoimidazole carboxylase catalytic subunit | 30.49 | 2 |
| Lpp0550 | PurA, adenylosuccinate synthetase | 16.12 | 5 |
| Lpp0559 | Adenosine deaminase | 21.00 | 1 |
| Lpp1184 | PyrG, CTP synthase | 17.44 | 1 |
| Lpp1366 | Adenylate kinase | 18.90 | 3 |
| Lpp1380 | PyrF, orotidine 5`-phosphate decarboxylase | 13.57 | 2 |
| Lpp1389 | Purine nucleoside phosphorylase | 38.71 | 2 |
| Lpp1646 | PurF, amidophosphoribosyltransferase | 74.48 | 2 |
| Lpp1647 | PurC, phosphoribosylamidoimidazole-succinocarboxamide synthase | 11.50 | 2 |
| Lpp1649 | PurM, phosphoribosylaminoimidazole synthetase | 31.68 | 3 |
| Lpp1650 | PurL, phosphoribosylformylglycinamidine synthase II | 13.50 | 4 |
| Lpp1677 | PyrH, uridine monophosphate kinase | 31.22 | 2 |
| Lpp1686 | Nucleoside deaminase | 5.00 | 1 |
| Lpp1738 | Ribonucleoside-diphosphate reductase, alpha subunit Rir1 | 16.99 | 3 |
| Lpp1992 | Guanosine monophosphate kinase | 44.23 | 3 |
| Lpp2005 | Carbamoyl-phosphate synthetase, small subunit | 48.71 | 3 |
| Lpp2902 | GMP reductase GuaC, IMPDH domain | 102.95 | 3 |
|  |  |  |  |
| **Fatty acid /Lipid metabolism, Butanoate/Propanoate (34 targets identified)** | |  |  |
| Lpp0014 | Patatin-like phospholipase, esterase of the alpha-beta hydrolase superfamily | 36.57 | 2 |
| Lpp0116 | 3-oxoacyl-[acyl-carrier-protein] synthase | 20.92 | 2 |
| Lpp0368 | Patatin-like phospholipase | 19.25 | 1 |
| Lpp0371 | Acyl-CoA dehydrogenase | 47.57 | 3 |
| Lpp0423 | Enoyl-(acyl carrier protein) reductase | 27.76 | 4 |
| Lpp0424 | Acyl carrier proteins acp | 12.18 | 1 |
| Lpp0425 | FabA/Z, 3-hydroxyacyl-acyl carrier protein (ACP)-dehydratase | 14.34 | 2 |
| Lpp0426 | FabF, 3-ketoacyl-acyl-carrier-protein (ACP)- synthase II | 24.47 | 5 |
| Lpp0528 | Acetyl-CoA carboxylase, biotin carboxylase subunit | 33.46 | 3 |
| Lpp0620 | Acetoacetyl-CoA reductase | 9.94 | 2 |
| Lpp0728 | Acetoacetate decarboxylase | 113.00 | 1 |
| Lpp0931 | Acyl-CoA dehydrogenase | 27.54 | 3 |
| Lpp1295 | Acetyl-CoA carboxylase, beta subunit | 32.64 | 3 |
| Lpp1347 | PlsX, fatty acid/phospholipid synthesis protein | 91.75 | 3 |
| Lpp1348 | FabH, 3-oxoacyl-[acyl-carrier-protein] synthase III | 152.79 | 2 |
| Lpp1349 | FabD, malonyl-CoA-[acyl-carrier-protein] transacylase | 43.54 | 3 |
| Lpp1351 | AcpP, acyl carrier protein | 7.14 | 4 |
| Lpp1352 | FabF, 3-oxoacyl-[acyl-carrier-protein] synthase II | 14.29 | 3 |
| Lpp1486 | PrpD, 2-methylcitrate dehydratase | 72.57 | 2 |
| Lpp1487 | PrpC, 2-methylcitrate synthase | 43.22 | 3 |
| Lpp1511 | long-chain fatty acid CoA synthetase | 50.18 | 2 |
| Lpp1554 | FadJ, multifunctional fatty acid oxidation complex, alpha subunit | 19.24 | 4 |
| Lpp1555 | FadI, multifunctional fatty acid oxidation complex, beta subunit | 14.49 | 3 |
| Lpp1691 | FadE, acyl-CoA dehydrogenase | 32.92 | 4 |
| Lpp1773 | FadL, long-chain fatty acid transport protein | 11.31 | 3 |
| Lpp1791 | Enoyl-CoA hydratase | 24.86 | 3 |
| Lpp1794 | Acetoacetyl-CoA synthetase | 59.71 | 3 |
| Lpp1925 | PatE, patatin-like phospholipase | 47.73 | 2 |
| Lpp2038 | PhbC polyhydroxyalkanoate synthase | 74.40 | 3 |
| Lpp2178 | Acetyl-CoA carboxylase, carboxyltransferase component | 11.38 | 1 |
| Lpp2264 | 3-hydroxybutyrate dehydrogenase | 14.34 | 3 |
| Lpp2296 | 2,4-dienoyl-CoA reductase | 24.07 | 3 |
| Lpp2305 | Glycerol-3-phosphate acyltransferase | 8.97 | 2 |
| Lpp2322 | Acetoacetyl-CoA reductase | 7.40 | 1 |
|  |  |  |  |
| **Transcription, RNA stability, Translation (46 targets identified)** | |  |  |
| Lpp0010 | HflX, GTP-binding protein | 17.21 | 2 |
| Lpp0022 | 23S rRNA m(2)G2445 methyltransferase | 8.38 | 2 |
| Lpp0106 | RNase R, exoribonuclease | 29.25 | 5 |
| Lpp0365 | Translation elongation factor P | 25.00 | 1 |
| Lpp0382 | NusG, transcription antitermination protein | 55.93 | 3 |
| Lpp0385 | 50S ribosomal subunit protein L1 | 16.33 | 2 |
| Lpp0416 | 30S ribosomal protein S13 | 17.09 | 4 |
| Lpp0465 | RimM, 16S rRNA processing protein | 8.70 | 1 |
| Lpp0481 | Ribosomal protein S6 modification enzyme, RimK-like ATP-grasp domain | 23.66 | 2 |
| Lpp0527 | PrmA, ribosomal protein L11 methyltransferase | 28.89 | 1 |
| Lpp0541 | Ribosome-associated sigma-54 modulation protein | 28.63 | 2 |
| Lpp0575 | Seryl-tRNA synthetase | 16.70 | 2 |
| Lpp0628 | Tyrosyl-tRNA synthetase | 23.74 | 2 |
| Lpp0651 | Fe-S cluster assembly SUF system transcriptional regulator | 5.55 | 1 |
| Lpp0749 | ProRS, prolyl-tRNA synthetase | 26.58 | 4 |
| Lpp0785 | Valyl-tRNA synthetase | 27.90 | 5 |
| Lpp0793 | NusB, transcription termination factor | 339.00 | 1 |
| Lpp0935 | Peptide chain release factor 3 | 14.63 | 3 |
| Lpp0999 | Isoleucyl-tRNA synthetase | 15.57 | 4 |
| Lpp1177 | Bacterial transcription regulatory protein, AsnC family | 10.86 | 1 |
| Lpp1249 | Transcriptional regulator, YebC/PmpR family | 17.18 | 2 |
| Lpp1266 | tRNA pseudouridine synthase A | 29.40 | 3 |
| Lpp1271 | Cysteinyl-tRNA synthetase | 40.70 | 3 |
| Lpp1288 | 2-methylthioadenine synthetase MiaB | 30.79 | 4 |
| Lpp1338 | Ribonuclease HI | 38.28 | 1 |
| Lpp1376 | 30S ribosomal protein S1 | 39.43 | 2 |
| Lpp1399 | TrpS, tryptophanyl-tRNA synthetase | 69.54 | 2 |
| Lpp1434 | Aspartyl-tRNA synthetase | 67.66 | 3 |
| Lpp1498 | EngA, ribosome-associated GTPase | 35.82 | 3 |
| Lpp1679 | 30S ribosomal protein S2 | 18.05 | 4 |
| Lpp1700 | Aspartyl/glutamyl-tRNA amidotransferase subunit C | 19.61 | 2 |
| Lpp1710 | IscS, cysteine desulfurase | 20.58 | 3 |
| Lpp1763 | Alanyl-tRNA synthetase | 33.30 | 4 |
| Lpp1803 | Glycyl-tRNA synthetase, beta subunit | 21.69 | 3 |
| Lpp1804 | Glycyl-tRNA synthetase, alpha subunit | 34.36 | 2 |
| Lpp1994 | Ribonuclease PH | 13.00 | 1 |
| Lpp2013 | Arginyl-tRNA synthetase | 26.14 | 3 |
| Lpp2236 | Asparaginyl-tRNA synthetase | 9.05 | 2 |
| Lpp2294 | DeaD, ATP-dependent RNA helicase | 20.90 | 4 |
| Lpp2247 | HepA, RNA polymerase-associated helicase protein | 35.16 | 2 |
| Lpp2326 | XRE family-like transcriptional regulator | 11.50 | 1 |
| Lpp2360 | Metal-dependent ribonuclease, beta-lactamase fold | 20.81 | 2 |
| Lpp2677 | GreA, transcription elongation factor | 46.17 | 2 |
| Lpp2705 | Ribosomal protein L25, Ctc-form | 52.83 | 1 |
| Lpp2891 | Tex-like transcriptional accessory protein | 45.34 | 3 |
| Lpp2931 | RNA pyrophosphohydrolase | 69.63 | 1 |
|  |  |  |  |
| **Regulation (16 targets identified)** | |  |  |
| Lpp0438 | Fur, ferric uptake regulation protein | 16.05 | 1 |
| Lpp0602 | LetE, transmission trait enhancer protein | 9.00 | 1 |
| Lpp0606 | Fis1, global DNA-binding transcriptional regulator | 51.24 | 2 |
| Lpp0809 | Signal transduction protein, GGDEF domain | 49.75 | 3 |
| Lpp0845 | CsrA, global regulator | 12.19 | 3 |
| Lpp0915 | FleQ, transcriptional regulator | 14.50 | 2 |
| Lpp1247 | RpoS, RNA polymerase sigma factor | 62.53 | 3 |
| Lpp1255 | PmrA, TCS response regulator | 25.69 | 3 |
| Lpp1324 | Fis2, DNA-binding protein | 50.83 | 1 |
| Lpp1413 | RelA, GTP pyrophosphokinase | 25.75 | 4 |
| Lpp1707 | Fis3, DNA-binding protein | 15.43 | 1 |
| Lpp1726 | FleR, response regulator | 49.19 | 2 |
| Lpp1826 | DNA-binding protein HU-beta | 5.52 | 1 |
| Lpp2084 | StuC, Sensor histidine kinase | 17.03 | 2 |
| Lpp2721 | RpoH, RNA polymerase sigma factor | 39.62 | 2 |
| Lpp2788 | LqsR, response regulator | 16.48 | 3 |
|  |  |  |  |
| **Cell envelope, Cell division, Motility (27)** | |  |  |
| Lpp0566 | UppS, undecaprenyl pyrophosphate synthase | 16.40 | 2 |
| Lpp0573 | LpxA, UDP-N-acetylglucosamine acyltransferase | 34.13 | 4 |
| Lpp0682 | Tfp pilus assembly protein, tip-associated adhesin PilY1-related protein | 9.57 | 1 |
| Lpp0689 | Glycosyltransferases, PMT family | 9.68 | 1 |
| Lpp0711 | Outer membrane protein, OmpA family | 27.17 | 4 |
| Lpp0814 | N-acetyl sugar amidotransferase, LPS biosynthesis protein | 39.83 | 4 |
| Lpp0820 | NeuD, sialic acid O-acetyltransferase | 27.91 | 3 |
| Lpp0827 | UDP-glucose 4-epimerase | 11.97 | 1 |
| Lpp0833 | Sialic acid synthase, NeuB family | 17.17 | 3 |
| Lpp0873 | MreB, rod shape-determining protein | 24.65 | 2 |
| Lpp0974 | MraZ, cell division protein | 58.19 | 3 |
| Lpp0978 | MurE, UDP-N-acetylmuramyl-tripeptide synthetase | 17.37 | 1 |
| Lpp0989 | PilM, type IV pilus assembly protein | 8.66 | 1 |
| Lpp1263 | FimV, transmembrane Tfp pilus assembly protein | 51.06 | 4 |
| Lpp1301 | LptE, lipopolysaccharide-assembly protein | 17.30 | 2 |
| Lpp1465 | RlpA, rare lipoprotein A | 18.25 | 2 |
| Lpp1503 | PilF, Tfp pilus assembly protein | 54.69 | 2 |
| Lpp1657 | FliK, flagellar hook-length control protein | 9.04 | 1 |
| Lpp1717 | UDP-N-acetylmuramate:L-alanyl-gamma-D-glutamyl-meso-diaminopimelate ligase | 47.50 | 4 |
| Lpp1730 | DNA segregation ATPase, FtsK/SpoIIIE family protein | 18.50 | 4 |
| Lpp1890 | PilA, Tfp pilus assembly protein | 26.71 | 1 |
| Lpp1995 | PilT, Tfp pilus assembly protein, pilus retraction ATPase | 11.00 | 1 |
| Lpp2026 | Peptidoglycan-associated lipoprotein, OmpA-like domain | 12.56 | 1 |
| Lpp2258 | Glutamate racemase | 19.15 | 3 |
| Lpp2624 | Putative rare lipoprotein A, (RlpA)-like double-psi beta-barrel domain | 7.50 | 2 |
| Lpp2662 | FtsZ, cell division protein | 16.69 | 3 |
| Lpp2840 | GlmM, phosphoglucosamine mutase | 15.22 | 2 |
|  |  |  |  |
| **Protein secretion/trafficking, Protein fate (41 targets identified)** | |  |  |
| Lpp0118 | MsrA, methionine sulfoxide reductase | 8.66 | 2 |
| Lpp0376 | SurA, peptidyl-prolyl cis-trans isomerase | 22.96 | 2 |
| Lpp0436 | RnfH family-related protein | 23.68 | 1 |
| Lpp0414 | SecY subunit, preprotein translocase | 25.46 | 3 |
| Lpp0467 | Ffh, signal recognition particle protein | 9.40 | 3 |
| Lpp0548 | HflK, protease subunit | 11.27 | 3 |
| Lpp0569 | YaeT, outer membrane protein assembly complex | 26.25 | 4 |
| Lpp0570 | Outer membrane protein, OmpH-like | 6.39 | 3 |
| Lpp0666 | Polypeptide deformylase | 10.70 | 2 |
| Lpp0694 | HslV, ATP-dependent protease, peptidase subunit | 21.22 | 2 |
| Lpp0695 | HslU, ATP-dependent protease, ATP-binding subunit | 21.16 | 2 |
| Lpp0742 | GroES or Cpn10, chaperonin 10 subunit | 9.68 | 2 |
| Lpp0743 | GroEL or Cpn60, chaperonin 60 | 9.39 | 2 |
| Lpp0755 | Protein-L-isoaspartate (D-aspartate) O-methyltransferase | 14.00 | 1 |
| Lpp0889 | Type I secretion outer membrane protein, TolC family | 14.44 | 4 |
| Lpp0509 | IcmR | 9.20 | 1 |
| Lpp0517 | IcmE/dotG | 48.35 | 4 |
| Lpp0522 | IcmH/dotU | 27.65 | 4 |
| Lpp0763 | IcmL-like protein | 24.34 | 1 |
| Lpp0956 | Type II secretion pathway protein C | 33.90 | 2 |
| Lpp1194 | Type 1 glutamine amidotransferase (GATase1), PfpI-like protein | 17.18 | 2 |
| Lpp1013 | TldD protease | 30.44 | 4 |
| Lpp1323 | Molecular chaperone, HSP90 family | 18.92 | 3 |
| Lpp1419 | SecA subunit, preprotein translocase | 26.88 | 4 |
| Lpp1626 | Zinc metalloprotease | 33.27 | 2 |
| Lpp1670 | Peptidase M20 family, glutamate carboxypeptidase | 18.02 | 2 |
| Lpp1684 | Methionine aminopeptidase 1 | 24.64 | 2 |
| Lpp1713 | SppA, signal peptide peptidase | 8.45 | 1 |
| Lpp1714 | ClpB, ATP-dependent chaperone | 13.45 | 3 |
| Lpp1830 | Trigger factor TF | 32.70 | 3 |
| Lpp1839 | Type II secretion system protein L | 7.53 | 1 |
| Lpp1840 | Type II secretion system protein M | 9.78 | 1 |
| Lpp1982 | SecD subunit, preprotein translocase, | 24.61 | 4 |
| Lpp2007 | DnaK, molecular chaperone | 24.49 | 4 |
| Lpp2008 | GrpE, molecular chaperone | 17.56 | 2 |
| Lpp2675 | Papain-like C1 peptidase | 21.70 | 3 |
| Lpp2684 | M17 family aminopeptidase | 22.58 | 3 |
| Lpp2730 | DotB, Dot/Icm secretion system ATPase | 107.32 | 3 |
| Lpp2742 | IcmW, Dot/Icm secretion system protein | 11.10 | 3 |
| Lpp2855 | Aminopeptidase N | 33.44 | 3 |
| Lpp2866 | Leucine aminopeptidase, Zn-peptidase M28 family | 54.08 | 3 |
|  |  |  |  |
| **Cofactors and Vitamins, Secondary Metabolite (25)** | |  |  |
| Lpp0092 | 2-octaprenyl-6-methoxyphenol hydroxylase | 16.52 | 4 |
| Lpp0377 | PdxA, 4-hydroxythreonine-4-phosphate dehydrogenase | 11.56 | 2 |
| Lpp0643 | 5,10-methenyltetrahydrofolate synthetase | 8.25 | 1 |
| Lpp0869 | NadC, nicotinate-nucleotide pyrophosphorylase | 12.71 | 2 |
| Lpp0939 | PntA, NAD(P) transhydrogenase alpha subunit | 57.47 | 3 |
| Lpp1008 | PdxJ, pyridoxine 5-phosphate synthase | 8.14 | 2 |
| Lpp1181 | Riboflavin synthase, alpha chain | 29.43 | 1 |
| Lpp1182 | RibA, riboflavin biosynthesis protein | 38.91 | 3 |
| Lpp1223 | HemF, coproporphyrinogen III oxidase | 43.00 | 4 |
| Lpp1522 | NMT1/THI5-like protein (thi operon) | 27.77 | 2 |
| Lpp1620 | IolE, inosose dehydratase | 82.50 | 1 |
| Lpp1622 | IolCB, bifunctional 5-dehydro-2-deoxygluconokinase/5-deoxy-glucuronate isomerase | 42.29 | 3 |
| Lpp1623 | IolG, myo-inositol 2-dehydrogenase | 32.50 | 3 |
| Lpp1712 | SuhB-like inositol monophosphatase | 10.41 | 4 |
| Lpp0927 | Dodecin | 46.21 | 2 |
| Lpp2003 | S-adenosyl-L-homocysteine hydrolase | 18.58 | 4 |
| Lpp2004 | S-adenosylmethionine synthetase | 15.83 | 1 |
| Lpp2146 | Ketosteroid isomerase, NTF2-like superfamily | 67.25 | 1 |
| Lpp2435 | Flavin reductase-like protein | 25.96 | 3 |
| Lpp2709 | IspB, octaprenyl-diphosphate synthase | 21.17 | 4 |
| Lpp2715 | PanB, 3-methyl-2-oxobutanoate hydroxymethyltransferase | 83.50 | 2 |
| Lpp2739 | Dienelactone hydrolase family protein | 5.96 | 2 |
| Lpp2890 | Acyl-CoA thioester hydrolase | 14.91 | 2 |
| Lpp2979 | Cu2+-containing amine oxidase | 46.83 | 3 |
| Lpp3001 | UbiD, 3-octaprenyl-4-hydroxybenzoate decarboxylase | 10.12 | 4 |
|  |  |  |  |
| **Transport, Uptake (21 targets identified)** | |  |  |
| Lpp0136 | ABC-type nitrate/sulfonate transport system, ATPase component | 35.86 | 4 |
| Lpp0236 | PvcA, pyoverdine biosynthesis protein | 8.76 | 1 |
| Lpp0288 | Heme oxygenase (iron acquisition) | 36.00 | 1 |
| Lpp0470 | Glutamate/gamma-aminobutyrate antiporter | 24.07 | 4 |
| Lpp0488 | Sugar transporter, MFS superfamily | 39.01 | 2 |
| Lpp0604 | PhtA, major facilitator superfamily (MFS) transporter | 18.11 | 2 |
| Lpp0613 | SMR family multidrug efflux pump, suppressor SugE of GroEL | 20.33 | 2 |
| Lpp0706 | PhtE, major facilitator superfamily (MFS) transporter | 26.67 | 4 |
| Lpp0710 | Tryptophan/tyrosine permease family protein | 61.71 | 1 |
| Lpp0713 | Efflux pump membrane fusion protein, HlyD family secretion protein | 19.29 | 3 |
| Lpp0714 | ABC-type multidrug transport system, ATPase component | 16.78 | 3 |
| Lpp0758 | ABC transporter, ATPase component | 18.10 | 3 |
| Lpp0838 | O-antigenic polysaccharide transporter, KpsT/Wzt ABC transporter family | 41.10 | 4 |
| Lpp1490 | Dipeptide/tripeptide permease | 7.00 | 1 |
| Lpp1624 | Sugar-proton symporter | 18.22 | 1 |
| Lpp1796 | MetN, DL-methionine transporter, ATP-binding subunit | 38.71 | 2 |
| Lpp1860 | PhtJ, major facilitator superfamily (MFS) transporter | 6.68 | 1 |
| Lpp2018 | Zinc/iron transport protein, ZIP family | 23.83 | 2 |
| Lpp2164 | Hbp, heme-binding protein | 11.07 | 2 |
| Lpp2372 | HelB, RND family efflux transporter | 24.90 | 4 |
| Lpp2606 | Large-conductance mechanosensitive channel | 22.66 | 2 |
|  |  |  |  |
| **DNA Replication, Recombination, Repair (17 targets identified)** | |  |  |
| Lpp0004 | DNA gyrase- subunit B (type II topoisomerase) | 12.10 | 4 |
| Lpp0113 | DNA polymerase I | 16.83 | 2 |
| Lpp0451 | Excinuclease ABC subunit A | 10.75 | 3 |
| Lpp0702 | Exonuclease III | 15.00 | 1 |
| Lpp0746 | DNA topoisomerase IV subunit B | 24.79 | 4 |
| Lpp0771 | Transposase, IS5 family | 58.36 | 1 |
| Lpp0803 | DnaB, replicative DNA helicase | 58.04 | 3 |
| Lpp0872 | ComEA, competence protein helix-hairpin-helix repeat region | 7.79 | 3 |
| Lpp1372 | DNA gyrase subunit A | 68.72 | 4 |
| Lpp1534 | RuvB, holliday junction DNA helicase | 45.44 | 3 |
| Lpp1765 | RecA, recombination protein | 41.32 | 5 |
| Lpp1897 | Rad3-related DNA helicase | 33.93 | 3 |
| Lpp2553 | RadC-like DNA repair protein | 105.67 | 3 |
| Lpp2803 | DNA-binding protein, YbaB/EbfC family | 9.80 | 3 |
| Lpp2952 | Very short patch repair (Vsr) endonuclease | 37.35 | 3 |
| Lpp2994 | Integrating conjugative element relaxase, PFL_4751 family | 8.43 | 2 |
| Lpp3051 | DNA topoisomerase IV- A subunit | 23.56 | 4 |
|  |  |  |  |
| **Virulence factors (48 targets identified)** | |  |  |
| Lpp0008 | RavA, substrate of the Dot/Icm secretion system | 33.63 | 2 |
| Lpp0012 | CegC1, substrate of the Dot/Icm secretion system | 14.81 | 4 |
| Lpp0047 | Substrate of the Dot/Icm secretion system | 69.39 | 2 |
| Lpp0234 | Substrate of the Dot/Icm secretion system | 7.00 | 2 |
| Lpp0332 | Substrate of the Dot/Icm secretion system | 11.09 | 2 |
| Lpp0469 | AnkG, substrate of the Dot/Icm secretion system, ankyrin repeat protein | 11.25 | 2 |
| Lpp0532 | Zinc metalloprotease, M4 family (major secreted protease) | 16.67 | 4 |
| Lpp0679 | StaR-like protein, TPR-domain - eukaryotic-like protein | 21.33 | 1 |
| Lpp0688 | Substrate of the Dot/Icm secretion system | 99.79 | 1 |
| Lpp0699 | RtxA, structural toxin protein | 44.76 | 5 |
| Lpp0751 | Substrate of the Dot/Icm secretion system | 18.90 | 2 |
| Lpp0799 | RavH, substrate of the Dot/Icm secretion system | 27.99 | 3 |
| Lpp0955 | Eukaryotic cytokinin dehydrogenase | 34.11 | 1 |
| Lpp0982 | MavT, substrate of the Dot/Icm secretion system | 34.37 | 3 |
| Lpp1002 | LidA protein - Rab1 sequestration, substrate of the Dot/Icm secretion system | 25.24 | 3 |
| Lpp1025 | Substrate of the Dot/Icm secretion system | 18.25 | 3 |
| Lpp1033 | Apyrase, eukaryotic ectonucleoside triphosphate diphosphohydrolase | 10.62 | 3 |
| Lpp1157 | Eukaryotic pyruvate decarboxylase | 61.48 | 2 |
| Lpp1168 | RavR, substrate of the Dot/Icm secretion system | 20.12 | 1 |
| Lpp1174 | LidL, Sel1-like TPR repeat protein (infection/manipulating vacuolar trafficking) | 38.82 | 1 |
| Lpp1236 | Substrate of the Dot/Icm secretion system | 9.00 | 1 |
| Lpp1283 | Substrate of the Dot/Icm secretion system | 38.90 | 5 |
| Lpp1445 | RavX, substrate of the Dot/Icm secretion system | 6.25 | 2 |
| Lpp1508 | RavY, substrate of the Dot/Icm secretion system | 15.53 | 2 |
| Lpp1546 | Substrate of the Dot/Icm secretion system | 31.75 | 1 |
| Lpp1632 | Substrate of the Dot/Icm secretion system | 9.28 | 1 |
| Lpp1667 | Substrate of the Dot/Icm secretion system | 25.21 | 3 |
| Lpp1740 | Substrate of the Dot/Icm secretion system | 13.06 | 1 |
| Lpp1766 | Substrate of the Dot/Icm secretion system | 80.62 | 1 |
| Lpp1805 | Com1-like membrane-associated immunoreactive protein, DsbA family | 5.00 | 1 |
| Lpp1848 | YlfB, substrate of the Dot/Icm secretion system | 59.93 | 1 |
| Lpp1942 | LirA, substrate of the Dot/Icm secretion system | 8.87 | 1 |
| Lpp2128 | Spl, substrate of the Dot/Icm secretion system, Sphingosine-1-phosphate lyase 1, | 21.70 | 4 |
| Lpp2150 | Substrate of the Dot/Icm secretion system | 14.57 | 2 |
| Lpp2163 | Arginase/histone deacetylase-like superfamily | 36.71 | 2 |
| Lpp2166 | Ankyrin repeat protein, substrate of the Dot/Icm secretion system | 21.21 | 3 |
| Lpp2237 | Conserved protein CetLp6 | 12.52 | 2 |
| Lpp2246 | YlfA, affect vesicle trafficking, substrate of the Dot/Icm secretion system | 27.47 | 4 |
| Lpp2272 | Eukaryotic-like sugar 1,4-lactone oxidase domain | 38.07 | 2 |
| Lpp2275 | Substrate of the Dot/Icm secretion system | 67.53 | 3 |
| Lpp2308 | Yqey-like protein, substrate of the Dot/Icm secretion system | 5.98 | 3 |
| Lpp2458 | SdbC effector protein - substrate of the Dot/Icm system | 19.85 | 3 |
| Lpp2486 | F-box domain, substrate of the Dot/Icm secretion system | 20.49 | 1 |
| Lpp2491 | MavH, substrate of the Dot/Icm secretion system | 13.74 | 1 |
| Lpp2594 | Substrate of the Dot/Icm secretion system | 33.42 | 3 |
| Lpp2943 | Substrate of the Dot/Icm secretion system | 7.70 | 1 |
| Lpp3047 | MavQ, substrate of the Dot/Icm secretion system | 19.77 | 2 |
| Lpp3071 | LegP, eukaryotic-like zinc metalloproteinase | 5.18 | 1 |
|  |  |  |  |
| **Stress response, Defense, Xenobiotica degradation (25 targets identified)** | |  |  |
| Lpp0148 | ProQ/FinO family protein | 45.00 | 1 |
| Lpp0165 | LexA-like protein, peptidase S24 - SOS-response transcriptional repressors | 19.69 | 4 |
| Lpp0191 | LexA-like protein, peptidase S24 - SOS-response transcriptional repressors | 8.84 | 3 |
| Lpp0252 | KatG, catalase/peroxidase | 19.68 | 5 |
| Lpp0472 | 4-carboxymuconolactone decarboxylase-family protein | 6.70 | 2 |
| Lpp0493 | CspD, cold shock-like protein | 14.80 | 3 |
| Lpp0654 | SufD, Fe-S cluster assembly protein | 30.86 | 2 |
| Lpp0657 | SufT, Fe-S cluster assembly protein | 54.21 | 2 |
| Lpp0744 | DNA starvation/stationary phase protection protein Dps | 7.94 | 1 |
| Lpp1207 | Cold-shock protein (CSP) | 21.26 | 2 |
| Lpp1219 | Thiocyanate hydrolase, gamma subunit | 22.98 | 2 |
| Lpp1731 | Thioredoxin reductase | 18.14 | 2 |
| Lpp1810 | Glutathione synthetase, prokaryotic | 27.43 | 4 |
| Lpp1883 | Glutathione S-transferase | 30.00 | 1 |
| Lpp2074 | Chemiosmotic efflux system protein B-like protein | 58.65 | 1 |
| Lpp2075 | Chemiosmotic efflux system protein C-like protein | 25.83 | 2 |
| Lpp2217 | Curved DNA-binding protein CbpA | 26.73 | 2 |
| Lpp2321 | Cold shock protein | 60.43 | 1 |
| Lpp2345 | CopA, copper-resistance protein | 22.63 | 3 |
| Lpp2353 | Chemiosmotic efflux system C protein C | 36.84 | 2 |
| Lpp2362 | Chemiosmotic efflux system B protein B | 8.56 | 2 |
| Lpp2375 | LexA-like SOS-response transcriptional repressors | 7.04 | 2 |
| Lpp2449 | Usp, universal stress protein | 41.31 | 2 |
| Lpp2454 | KatB, catalase-peroxidase | 17.47 | 4 |
| Lpp2901 | PhoH-like protein, phosphate starvation induced ATPase | 69.97 | 5 |
|  |  |  |  |
| **Unknown function, Hypothetical protein; Others (91 targets identified)** | |  |  |
| Lpp0073 | Phage derived protein of unknown function Gp49-like (DUF891) | 15.01 | 2 |
| Lpp0090 | Uncharacterized protein family (UPF0149) | 13.67 | 1 |
| Lpp0101 | Protein of unknown function (DUF4254) | 12.83 | 3 |
| Lpp0122 | Protein of unknown function | 17.71 | 1 |
| Lpp0187 | Hypothetical protein | 67.37 | 1 |
| Lpp0201 | Hypothetical protein with transposase-like domain | 27.28 | 2 |
| Lpp0202 | Ankyrin repeat protein | 43.15 | 4 |
| Lpp0203 | Hypothetical protein with transposase-like domain | 23.08 | 2 |
| Lpp0207 | Hypothetical protein with transposase-like domain | 35.71 | 3 |
| Lpp0226 | Hydrolase, alpha/beta family | 94.86 | 1 |
| Lpp0238 | NAD/FAD-dependent oxidoreductase | 52.15 | 4 |
| Lpp0255 | Hypothetical protein, putative heavy-metal-binding domain | 8.07 | 1 |
| Lpp0256 | Hypothetical protein | 5.50 | 2 |
| Lpp0257 | CbpD, chitin-binding protein | 16.33 | 1 |
| Lpp0303 | Hypothetical protein | 63.21 | 2 |
| Lpp0307 | Hydrolase, alpha/beta family | 125.15 | 1 |
| Lpp0317 | Hypothetical protein | 33.50 | 2 |
| Lpp0324 | Hypothetical protein | 22.69 | 2 |
| Lpp0343 | Protein of unknown function (DUF853) | 17.83 | 2 |
| Lpp0441 | Protein of unknown function | 12.28 | 3 |
| Lpp0506 | Hypothetical protein | 34.00 | 1 |
| Lpp0605 | Protein of unknown function (DUF945) | 37.55 | 3 |
| Lpp0640 | Mg2+ chelatase-related protein, predicted ATPase with chaperone activity | 8.51 | 1 |
| Lpp0649 | Hypothetical protein | 14.03 | 2 |
| Lpp0662 | Hemolysin-related protein, containing CBS domain | 14.84 | 1 |
| Lpp0671 | Major outer membrane protein | 115.43 | 2 |
| Lpp0712 | Hypothetical protein | 11.22 | 2 |
| Lpp0039 | Uncharacterized protein, GIY-YIG domain | 10.35 | 1 |
| Lpp0725 | Predicted integral membrane protein (DUF2282) | 13.23 | 1 |
| Lpp0740 | Radical SAM methylthiotransferase, MiaB/RimO family | 28.86 | 2 |
| Lpp0790 | Predicted periplasmic protein containing BON-like domain | 39.51 | 3 |
| Lpp0797 | Hypothetical protein | 76.31 | 2 |
| Lpp0802 | Uncharacterized protein conserved in bacteria (DUF2147) | 34.45 | 2 |
| Lpp0813 | IraA, small-molecule methyltransferase | 24.09 | 2 |
| Lpp0866 | Ntn hydrolase | 12.27 | 2 |
| Lpp0890 | Predicted transglutaminase-like cysteine proteinase | 14.28 | 3 |
| Lpp0893 | Flavin-binding monooxygenase-like protein | 29.42 | 3 |
| Lpp0964 | Conserved protein of unknown function | 9.01 | 2 |
| Lpp1019 | Hypothetical protein | 116.33 | 1 |
| Lpp1100 | Ankyrin repeat protein | 39.66 | 4 |
| Lpp1105 | Conserved protein of unknown function | 44.94 | 3 |
| Lpp1122 | Conserved protein of unknown function | 34.49 | 2 |
| Lpp1164 | Hypothetical membrane protein | 14.89 | 3 |
| Lpp1297 | DedD, uncharacterized protein, contains sporulation-related domain | 18.31 | 4 |
| Lpp1305 | Aldehyde dehydrogenase family protein | 23.28 | 4 |
| Lpp1326 | Oxidoreductase family protein | 30.08 | 3 |
| Lpp1342 | Hypothetical protein | 19.74 | 1 |
| Lpp1382 | Short-chain dehydrogenase SDR | 22.71 | 3 |
| Lpp1438 | Conserved protein of unknown function | 15.07 | 3 |
| Lpp1540 | Hypothetical protein, partial L,D-transpeptidase catalytic domain | 26.43 | 2 |
| Lpp1548 | Transmembrane protein of unknown function | 16.48 | 3 |
| Lpp1578 | Protein of unknown function (DUF559), predicted endonuclease | 31.14 | 2 |
| Lpp1618 | YceI-like protein | 33.84 | 1 |
| Lpp1619 | Predicted integral membrane protein (DUF2282) | 7.11 | 1 |
| Lpp1625 | Tetratricopeptide repeat TPR-domain containing protein | 9.17 | 1 |
| Lpp1634 | Hypothetical protein | 27.43 | 1 |
| Lpp1655 | Short chain dehydrogenase | 12.00 | 1 |
| Lpp1772 | Conserved protein of unknown function | 15.34 | 3 |
| Lpp1783 | N-acetyltransferase, GNAT family | 47.88 | 1 |
| Lpp1800 | Conserved protein of unknown function | 14.80 | 3 |
| Lpp1814 | Hypothetical protein | 11.01 | 2 |
| Lpp1856 | Alpha/beta hydrolase | 32.64 | 2 |
| Lpp1858 | Hypothetical protein | 11.00 | 2 |
| Lpp1869 | Protein of unknown function (DUF559), related to endonucleases | 42.36 | 2 |
| Lpp1898 | 4Fe-4S binding protein, ferredoxin-like | 19.45 | 4 |
| Lpp1958 | *Legionella* major outer membrane protein | 15.44 | 3 |
| Lpp2099 | Conserved protein of unknown function | 14.56 | 2 |
| Lpp2102 | Hypothetical protein | 71.56 | 2 |
| Lpp2159 | Medium chain dehydrogenases/reductase, MDR family | 14.52 | 3 |
| Lpp2179 | Hypothetical protein | 51.75 | 3 |
| Lpp2193 | Alpha/beta hydrolase | 25.62 | 2 |
| Lpp2209 | Membrane protein of unknown function | 60.15 | 2 |
| Lpp2231 | O-methyltransferase | 27.75 | 1 |
| Lpp2243 | Hypothetical protein | 44.83 | 2 |
| Lpp2478 | GAF-domain protein | 13.00 | 1 |
| Lpp2557 | CBS-domain protein | 38.57 | 2 |
| Lpp2612 | Hypothetical protein | 11.17 | 1 |
| Lpp2697 | Collagen triple helix repeats-containing protein | 20.90 | 3 |
| Lpp2713 | AFG1-like ATPase, AAA domain | 34.27 | 3 |
| Lpp2796 | Uncharacterized protein conserved in bacteria (DUF208) | 20.14 | 2 |
| Lpp2894 | GDSL-like hydrolase | 25.19 | 3 |
| Lpp2917 | MoxR-like ATPase | 19.35 | 4 |
| Lpp2925 | Thioesterase, hotdog fold superfamily | 9.55 | 2 |
| Lpp2929 | Pembrane protein, predicted sulfite exporter TauE/SafE | 43.96 | 2 |
| Lpp2968 | Hypothetical protein | 29.65 | 4 |
| Lpp2978 | Protein of unknown function (DUF4169) | 32.75 | 2 |
| Lpp3017 | Conserved protein of unknown function | 56.68 | 3 |
| Lpp3020 | Glycosyltransferase, GT1 family | 22.29 | 3 |
| Lpp3021 | Conserved protein of unknown function | 31.00 | 1 |
| Lpp3024 | Hypothetical protein | 25.39 | 2 |
| Lpp3063 | Outer membrane lipoprotein, BON domain | 16.09 | 3 |
|  |  |  |  |
|  |  |  |  |
| **Small RNAs (5 targets identified)** | |  |  |
| ncRNA | Lppnc0639 (ncRNA20) | 20.00 | 1 |
| ncRNA | rsmX | 10.30 | 3 |
| ncRNA | rsmY | 23.89 | 2 |
| ncRNA | rsmZ | 18.61 | 4 |
| ncRNA | Lppnc0013 | 12.17 | 2 |
| FC, Fold change indicating the enrichment of the target sequences as compared to the control sample. Numbers indicate the average value obtained form the positive RIPseq experiments. Enrichment was calculated with the program sliding_window_peak_calling_script.py [38]. N° of IP, number of experiments in which the target sequence was identified. Of the 479 target genes identified, 21% were detected in one, 31.5% in two, 29.9% in three, 14.8% in four and 2.5% in all five RIPseq libraries. | | | |
