# Supplementary material for: The Legionella pneumophila genome evolved to accommodate multiple regulatory mechanisms controlled by the CsrA-system
Source: PLoS Genet. 2017 Feb 17;13(2):e1006629. doi: 10.1371/journal.pgen.1006629 (PMC5338858; doi:10.1371/journal.pgen.1006629)
Supplement: S4 Table — (DOCX) [file pgen.1006629.s017.docx]

| Sample | Control ^a^ | CsrA co-IP ^b^ | Enrichment  (CsrA co-IP/control) ^c^ |
| --- | --- | --- | --- |
| IP-1 | 0.50 | 1.50 | 2.94 |
| IP-2 | 0.60 | 1.04 | 1.73 |
| IP-7 | 0.73 | 1.38 | 1.89 |
| IP-8 | 0.61 | 0.81 | 1.32 |
| IP-9 | 0.74 | 0.92 | 1.24 |
| ^a^ Average number of A(N)GGA motifs per peak calculated in "negative" control peaks  ^b^ Average number of A(N)GGA motifs per peak calculated in CsrA targets peaks  ^c^ Enrichment of A(N)GGA motifs found in CsrA target peaks defined in the co-IP as compared to those found in the control IP | | | |

**Table S4 Enrichment of GGA motifs in the peaks of the co-IP as compared to the control IP**
