# Supplementary material for: The Legionella pneumophila genome evolved to accommodate multiple regulatory mechanisms controlled by the CsrA-system
Source: PLoS Genet. 2017 Feb 17;13(2):e1006629. doi: 10.1371/journal.pgen.1006629 (PMC5338858; doi:10.1371/journal.pgen.1006629)
Supplement: S5 Table — (DOCX) [file pgen.1006629.s018.docx]

**Table S5: Targets identified in all three approaches (RIPseq, transcriptome and proteome data)**

| Gene | Description | RIPseq | Tran | Prot |
| --- | --- | --- | --- | --- |
| *lpp0234* | Substrate of the Dot/Icm secretion system | 7.00 | 0.45 | 0.61 |
| *lpp0332* | Substrate of the Dot/Icm secretion system | 11.09 | 0.31 | 0.36 |
| *lpp0359* | NAD-dependent formate dehydrogenase | 14.50 | 3.18 | 6.48 |
| *lpp0493* | CspD, Cold shock-like protein | 14.80 | 6.57 | 17.3 |
| *lpp0559* | Adenosine deaminase | 21.00 | 0.46 | 0.44 |
| *lpp0663* | Zn-dependent alcohol dehydrogenase | 6.01 | 1.63 | 2.69 |
| *lpp0706* | PhtE, Major facilitator superfamily (MFS) | 26.6 | 0.58 | 0.40 |
| *lpp0725* | Predicted integral membrane protein (DUF2282) | 13.23 | 4.84 | 4.14 |
| *lpp0727* | NADH dehydrogenase, FAD-containing subunit | 20.27 | 2.15 | 2.74 |
| *lpp0728* | Acetoacetate decarboxylase | 113.00 | 3.84 | 2.60 |
| *lpp0809* | Signal transduction protein, GGDEF domain | 49.75 | 1.65 | 1.78 |
| *lpp0872* | Competence protein comea helix-hairpin-helix repeat | 7.78 | 4.96 | 7.26 |
| *lpp0939* | NAD(P) transhydrogenase alpha subunit pnta | 57.46 | 1.98 | 2.60 |
| *lpp0964* | Conserved protein of unknown function | 9.00 | 2.58 | 6.07 |
| *lpp0982* | MavT, Substrate of the Dot/Icm secretion system | 27.90 | 1.53 | 2.68 |
| *lpp1025* | Substrate of the Dot/Icm secretion system | 18.25 | 4.04 | 3.57 |
| *lpp1177* | Bacterial transcription regulatory protein, AsnC family | 10.85 | 10.7 | 5.38 |
| *lpp1181* | Riboflavin synthase, alpha chain | 29.42 | 0.62 | 0.61 |
| *lpp1182* | RibA, Riboflavin biosynthesis protein | 38.91 | 0.59 | 0.62 |
| *lpp1324* | Fis2, Global DNA-binding transcriptional regulator | 50.83 | 3.42 | 4.52 |
| *lpp1389* | Purine nucleoside phosphorylase | 38.70 | 0.36 | 0.57 |
| *lpp1438* | Conserved protein of unknown function | 15.07 | 0.51 | 0.57 |
| *lpp1546* | Substrate of the Dot/Icm secretion system | 31.75 | 0.49 | 0.30 |
| *lpp1707* | Fis3, Global DNA-binding transcriptional regulator | 15.42 | 0.35 | 0.47 |
| *lpp1766* | Substrate of the Dot/Icm secretion system | 80.61 | 0.35 | 0.44 |
| *lpp1800* | Conserved protein of unknown function | 14.79 | 1.54 | 5.57 |
| *lpp1826* | HU-beta, DNA-binding protein | 5.52 | 1.78 | 2.84 |
| *lpp1856* | Alpha/beta hydrolase | 32.63 | 4.63 | 2.32 |
| *lpp1883* | Glutathione S-transferase | 30.00 | 2.87 | 2.90 |
| *lpp1995* | PilT, Tfp pilus assembly protein, pilus retraction atpase | 11.00 | 1.57 | 1.86 |
| *lpp2128* | *Lp*Spl, Eukaryotic-like sphingosine-1-phosphate lyase | 21.69 | 0.29 | 0.45 |
| *lpp2163* | Arginase/histone deacetylase-like superfamily | 36.71 | 1.77 | 3.70 |
| *lpp2209* | Membrane protein of unknown function | 60.15 | 4.80 | 2.17 |
| *lpp2230* | Amino acid (Glu/Leu/Phe/Val) dehydrogenase | 45.68 | 0.60 | 0.52 |
| *lpp2247* | HepA, RNA polymerase-associated helicase protein | 35.15 | 0.63 | 0.52 |
| *lpp2264* | 3-hydroxybutyrate dehydrogenase | 14.34 | 1.93 | 3.16 |
| *lpp2272* | Eukaryotic-like sugar 1,4-lactone oxidase domain | 38.07 | 1.84 | 8.57 |
| *lpp2275* | Substrate of the Dot/Icm secretion system | 67.52 | 0.35 | 0.27 |
| *lpp2322* | Acetoacetyl-CoA reductase | 7.40 | 3.66 | 11.6 |
| *lpp2362* | Chemiosmotic efflux system B protein B | 8.56 | 1.84 | 3.05 |
| *lpp2435* | Copper-resistance protein, copa family | 25.90 | 0.12 | 24.4 |
| *lpp2458* | Sdbc, Substrate of the Dot/Icm system | 19.80 | 0.57 | 0.32 |
| *lpp2675* | Papain-like C1 peptidase | 21.69 | 3.43 | 2.67 |
| *lpp2715* | 3-methyl-2-oxobutanoate hydroxymethyltransferase | 83.50 | 1.92 | 1.63 |
| *lpp2739* | SpbA, Small basic protein | 5.95 | 1.64 | 1.63 |
| *lpp2894* | GDSL-like hydrolase/fatty acyltransferase | 25.18 | 2.17 | 1.74 |
| *lpp3021* | Conserved protein of unknown function | 31.00 | 2.52 | 3.10 |
| *lpp3047* | MavQ, Substrate of the Dot/Icm secretion system | 19.77 | 1.54 | 1.67 |
