# Supplementary material for: The Legionella pneumophila genome evolved to accommodate multiple regulatory mechanisms controlled by the CsrA-system
Source: PLoS Genet. 2017 Feb 17;13(2):e1006629. doi: 10.1371/journal.pgen.1006629 (PMC5338858; doi:10.1371/journal.pgen.1006629)
Supplement: S6 Table — (DOCX) [file pgen.1006629.s019.docx]

**Table S6: *In vitro* RNA used in the Electrophoretic Mobility Shift Assays with CsrA (potential CsrA-binding sides are highlighted in yellow, start codon in red)**

>RsmX-RNA

cgaggaggcggaauuggagcaucaggcaagauggaagccgcauggauuguggcguucaauucccccucgaccaagcuuauuguauuguaaggcuuuguuuu

>Lppnc0639-RNA

ggaacguuauaacacguuucuuuaaguccaaauaucggacgcugauguuacuugaaggaacaaguaucgcagggcaguccaaucaacagaaaaagugguuuaaccauagcgguuaaccucgcauugcuuggcuugaaugaaccccuucauuugggccauuucuaaguuuuaaagggugauauguucuagauuuuu

>FleQ-RNA FleQ1 FleQ2

aaaugacucaaacuuaaggauagguuu**aug**aggagcaaugacaggauuuauauuaucgaugaugauaaggaacguugugacaa

>LqsR-RNA LqsR2

aaguuagccagguucugaugauggagccauagguuauuugaaguucauagaaaggauauaacuuuggaacguuuugcucaggaggagcc**aug**caacauuucucaauacc

LqsR1

>LetE-RNA

aagugugggaguuacaugcacuaaaaagcaguucugcaccaauugcguuaauaauuauagauuuaggaguaaaugaguugaaaagcggaaauauauucaggauacauaaaagaggcuaucauc**aug**aacgauacaacaucauuau

>LetE TSS2-RNA (not found in RIPseq, neg. control)

aaaaucaauaaguuaaugaaguaucguguuaaagaaccaaagguaaaaagauuuuugucaagauaauguaaauauuugauuuucgaaggcagggggcaaauuaaagucgu

>RelA-RNA

augucgagucuauugccuuguuucaaaaaggauaauaugu**aug**guaagaguaaaagauacgacuccguugaugccagauggcaguaucgacguagagaugugguuacaucaucuugguucaaaaggauacuuggauaaucucgaacucguaaga

>PhbC-RNA

auaucuuguugauuauugggcguaaugcccagg**aug**ucagccaugaaggggcuagaaaaacaaugcuguucuaaggaaaggauacucacucagagc

>Gap-RNA

gcucagauguuagcauagaaggaaacaugcugauuguugaugggcauggcauucaagugauugcugagcgugauccggccaaacugccauggaaacaauuggacauugauaucgucuuugaau

>Fur1-RNA

gcuggaauaaugaucaaagcucgaaauucguuacaguuaaggagcacaa**gug**gaagagagucaacaguuaaaagacgcuggauuaaaaaucacauuaccucguaucaagguauugcaaaua

>Fur2-RNA

guaucgaggcauaauuuugaaggaggacacucgguguuugaguugucucagggcgaacaucaugaccaucuuguuuguguuaagugugggcggguagaggaauuuguagaugaaauaauagaacaaaga

>lpp1033-RNA

guuacuugaggugaauuaaggaaagacu**aug**uugcgauggaucguguuaauuuguaucucuuuguuucugccuuuaucugcuuaugcggacaaauccgauu

>LidA-RNA

ggcgauugauuaaggauccuauuu**aug**gcaaaagauaacaaaucacaucaaguuaaaacaucagaagggagucuugaauccguaaaaaccaaggaaaaggaaccuguuguagaaaaaaugcguguugaggauaguaaa

>YlfA-RNA

cuaaggaagauuuuuaaucguaaggaguaaau**aug**gcuacuaaugaaacagagcuucaaguauugauccaacaugauagcaaaucaacaaucacuacuucuagcuuggacaguacugauaaggau

>Hfq start region (not found in RIPseq, neg. control)

uaguuagauacccguuuuugccuuuaaaaauuacaauaacaacaaggaguaggca**aug**ucuaaaaaucauuuacuacaagacccuuuccuaaaugaauugcgcaaggaaaagguaccuguuucaguguuccuggucaaugguauuaaauugca

>RpoS

gauacaaugcaag**aug**aagaagagccaauuaaggacaaggaauuuaaagaagaggaauggucugagccagaugaugacucucuguugucagaagaggauauugaaccugauauugaaaaaauggaagaagagcuugaugaacuuccagaguuuacugaugaugaagcu

>Thi (TPP-Riboswitch)

ggcgcggggugucgggaaauccggcugagaguuacccguugaacuugaucugaaucauaucagcguaaggacgccuucaaaaaaaccauggccuuuuuaaggcaugguucccuuuugccaucuccauaauauuuaaaaauaauaacauaaggaguuauggcg**aug**ucaucacuaaaauccaggguuacccuacugcuuaauugguacacuaacccuuaucauacc
