# Supplementary material for: The Legionella pneumophila genome evolved to accommodate multiple regulatory mechanisms controlled by the CsrA-system
Source: PLoS Genet. 2017 Feb 17;13(2):e1006629. doi: 10.1371/journal.pgen.1006629 (PMC5338858; doi:10.1371/journal.pgen.1006629)
Supplement: S7 Table — (DOCX) [file pgen.1006629.s020.docx]

**Table S7: Additional primers used in this study**

| gap-RT_F  gap-RT_R | TTGATACGACAGTGGTCTATGG  CATGGACAGTGTTGACTAAGCC |
| --- | --- |
| tkt-RT_F  tkt-RT_R | GTTATAGCCAGTTGAACCTCTG  TGTTACTCTCACGACAAAATCTGC |
| fur-RT_F  fur-RT_R | CATTACCTCGTATCAAGGTATTGC  GAACTCTATATACAGTAGCTAACC |
| thi-RT_F  thi-RT_R | CATACTTGTGGCACAACAATTGGG  GCTGCAACGGTATGAATCATTGC |
| tpp-RT_F  tpp-RT_R | ATGGCGCGGGGTGTCGGGAAATC  AGGGAACCATGCCTTAAAAAGGC |
| tldD-RT_F  tldD-RT_R | AATCGGAACGTCGATGATGCTG  ATCCCTACCCCCTTATCCAGAG |
| gyrB-RT_F  gyrB-RT_R | AGCGTAGACGCCAGTTATGATTC  TGATGCAAACCGGTTCCATCATC |
| pLqsR_F  pLqsR_R  m2LqsR_R | GAATTCAAGTTAGCCAGGTTCTGATGATGG  CATATGGCTCCTCCTGAGCAAAACG  CATATGGCCTCTCCAGAGCAAAACGTTCC |
| LqsR-Mut_F  LqsR-Mut_R | AGTTCATAGAAAAAATATAACTTTGGAACGTTTTGCTC  AGTTATATTTTTTCTATGAACTTCAAATAACCCTATGG |
| pRelA_F  pRelA_R  mRelA_R | GAATTCTAACAGCTGGGGTAATGGATATGTTTC  CATATGGAGAATTATCCAAGTATCCTTTTGAACC  CATATGGAGAATTATCCAAGTATTTTTTTGAACCAAGATG |
| m1RelA_F  m1RelA_R | TCGAGTCTATTGCCTTGTTTCAAAGAGAATAATATGTATGG  CTTTTACTCTTACCATACATATTATTCTCTTTGAAACAAGGC |
| pFleQ_F  pFleQ_R | GAATTCAAATGACTCAAACTTAAGGATAGGTTTATG  CATATGCTTATCATCATCGATAATATAAATCCTG |
| mFleQ_F  mFleQ_R | GAATTCAAATGACTCAAACTTAAGAGTAGGTTTATG  CATATGCTTATCATCATCGATAATATAAATTCTG |
| RpoS_F  pRpoS_R  mRpoS_R | GAATTCGTTAAGAAGTAAAAAACTATTTCAAGGC  CATATGAGACCATTCCTCTTCTTTAAATTCCTTGTCCTTAATTGG  CATATGAGACCATTTCTCTTCTTTAAATTTCTTGTCCTTAATTGG |
| pThi_F  pThi_R | GAATTCTAGATGGCGCGGGGTGTCGGG  GAATTCAAAAGGGAACCATGCCTTAAAAAGG |
| mTPP_F | GAATTCTAGATGGCGCGGGGTGTCGGGAAATCCGGCTAAAAGTTACCC |
| mCsrA_F  mCsrA_R | CTGTAAGAACGCCTTCAAAAAAACCATG  CGTTCTTACGCTGATATGATTCAGATC |
| mGap_F  mGap_R | CATGTTTTTTTCTATGCTAACATCTGAGCG  CATAGAAAAAAACATGCTGATTGTTGATGG |
| m2Gap_F  m2Gap_R | CGAAAAAAACCGTGAGTTGAATCGTAGC  GGTTTTTTTCGCTCAGATGTTAGCATAG |
| Fur-Mut_F  Fur-Mur_R | TTCGAGATTACCTTTTCCTCGTC  AGAATAGGAGGCTCTCACGAAG |
| Fur-Inv_F  Fur-Inv_R | AGCGGATCGGGGATTGTCTTATCGAGGCATAATTTTGAAGGAGG  GCTGATGGAGCTGCACATGAATTGACTCTCTTCCACTTGTGCTC |
| T7-gapNB_F  gapNB_R | TGTAATACGACTCACTATAGGGCACGTCGTAAATCTTTATGGC  GATGTCATTGTATCTAACGCTTCCTG |
| T7-tktNB_F  tktNB_R | TGTAATACGACTCACTATAGGGCAGGTCGCCATTGGCACG  AGCGCACGGTTCTCCATTGAGTGC |
